# A novel method for the mechanochemical synthesis of unsymmetrical disulfides using phosphorodithioic acid derivatives

**DOI:** 10.1038/s41598-025-29563-5

**Published:** 2025-12-05

**Authors:** Mikołaj Walter, Agata Grobelna, Janusz Rachoń, Dariusz Witt, Sebastian Demkowicz

**Affiliations:** Department of Organic Chemistry, Faculty of Chemistry, Gdańsk University of Technology, Narutowicza 11/12, Gdansk, 80-233 Poland

**Keywords:** Mechanochemistry, Vibrational ball mill, Solvent-free synthesis, Thiols, Disulfides, Chemistry, Materials science

## Abstract

In this study, we report the efficient synthesis of unsymmetrical disulfides using a mechanochemical ball-milling approach. This research successfully yielded alkyl-alkyl, alkyl-aryl, and aryl-aryl unsymmetrical disulfides without any detectable formation of symmetrical products. Additionally, we eliminated the use of solvent in the reaction mixture and replaced triethylamine with the more environmentally friendly cesium carbonate as a base. These results represent a significant advancement in the modern synthesis of disulfides, which have applications in pharmacology.

**Supplementary Information:**

The online version contains supplementary material available at 10.1038/s41598-025-29563-5.

## Introduction

In the pharmaceutical industry alone, solvents account for the majority of chemicals used per gram of product obtained^1^, which translates to substantial quantities at large production scales. Although solvents are often purified after the reaction and reused in subsequent processes, these operations are time- and energy-intensive and can result in the emission of solvents into the environment. On an industrial scale, this poses a significant threat to the environment. Given the growing emphasis on developing synthetic methods aligned with the principles of “green chemistry”—primarily driven by the depletion of fossil fuel reserves from which most industrial chemicals are derived—many researchers are increasingly turning to mechanochemical procedures. Mechanochemistry is one of the few techniques that enables synthesis in solid-state conditions, thereby completely eliminating solvents from reaction mixtures^2^. Mechanochemistry refers to any technique in which a chemical reaction is initiated by the mechanical energy applied to the reactants^3^. This energy can be generated by processes such as grinding, shearing, or compressing chemicals. While mechanochemical reactions are predominantly conducted by grinding reactants in ball mills, some examples in the literature describe reactions performed by grinding in a mortar However, to avoid operator dependence, these processes are often automated^4^. There is also growing interest in continuous flow processes using screw extruders^5^. In addition to environmental benefits, mechanochemical methods offer numerous advantages over conventional conditions in chemical synthesis. Reports in the literature frequently highlight reduced reaction times when using ball milling techniques^6–8^. Mechanochemical reactions often achieve higher yields than solvent-based approaches. In some cases, these methods alter the selectivity of a reaction^9,10^ or even enable the synthesis of completely different products^11,12^. Another notable advantage is that ball milling can have significantly lower energy requirements compared to heat-based procedures^13^. Combined with the absence of solvent use, this results in cost reduction for such syntheses. These advantages are likely attributed to the higher concentration of molecules during the reaction and the absence of solvation effects.

Although mechanochemistry allows for the elimination of liquid compounds from reaction mixtures, there are instances where the addition of a fluid is beneficial or even necessary due to the nature of the reagents. For such cases, a specialized synthetic technique known as Liquid-Assisted Grinding (LAG)^14^ was developed. When a small quantity of fluid—typically not exceeding 2 µg/mg of solid substances—is added to the system, reaction times can be rapidly shortened. This approach is commonly employed in the mechanochemical synthesis of many active pharmaceutical ingredients (API)^15,16^.

Organosulfur compounds, especially those containing S‒S bonds, play a crucial role in various fields, including nanomaterials synthesis, agriculture, polymer chemistry, inorganic and organic synthesis, and pharmaceutical chemistry. In medical applications, disulfides can function indirectly as drug carriers^17^ or directly as active pharmaceutical ingredients^18^. These sulfur-sulfur bond-containing compounds exhibit excellent biocompatibility and are readily cleaved by disulfide reductase in targeted areas of the body, enabling effective treatment. Recently, disulfides have gained increased attention as encapsulating agents^17^. When used directly as active pharmaceutical ingredients, disulfides are employed in the treatment of various diseases, notably for their antifungal and antimicrobial properties^18^. Additionally, they have applications in treating certain cancers^19^ and Acquired Immune Deficiency Syndrome^20^. The mechanism of action for these compounds involves deactivating biochemical pathways by interacting with enzyme active sites, thereby rendering the enzymes inactive in metabolic processes. Among disulfides, unsymmetrical ones are particularly valuable due to their electrophilic center localized on one of the sulfur atoms. This electronic arrangement enhances the cleavage of S‒S bonds when attacked by nucleophiles at the enzyme’s active site.

5,5-Dimethyl-2-sulfanyl-2-thioxo-1,3,2-dioxaphosphorinane derivatives have been utilized in the synthesis of various classes of chemical compounds, including unsymmetrical disulfides^21,22^ and α-sulfenylated carbonyl compounds^23^. These derivatives are of significant interest due to their exceptional properties. First, most of these derivatives are crystalline solids with excellent stability, making them well-suited for long-term storage and ideal for use in solid-state synthesis. Additionally, the phosphorodithioic acid anion serves as an outstanding leaving group, significantly enhancing the reactivity of the conjugated thiol. Finally, the use of phosphorodithioic acid derivatives facilitate distinguishing products from substrates via ^31^P NMR analysis, providing a convenient method for monitoring reaction progress.

The present study presents the development of a synthetic procedure for the mechanochemical synthesis of unsymmetrical disulfides using 5,5-dimethyl-2-sulfanyl-2-thioxo-1,3,2-dioxaphosphorinane derivatives.

## Materials and methods

5,5-Dimethyl-2-sulfanyl-2-thioxo-1,3,2-dioxaphosphorinane derivatives were synthesized following a previously described procedure^21^. The appropriate thiol derivatives and cesium carbonate were purchased from Sigma Aldrich. Reactions were conducted using an Anton Paar BM500 vibrational ball mill with stainless-steel milling beakers (5 mL) and stainless-steel milling balls. ^1^H NMR spectra were recorded on a **Varian Unity Inova 500 (500 MHz)** spectrometer, with chemical shifts δ reported in parts per million relative to the residual solvent peak (DMSO-d_6_ = 2.49 ppm for 1 H). Coupling constants are provided in Hertz. High-resolution mass spectra were obtained using an Agilent 6545 Q-TOF spectrometer. Thin-layer chromatography (TLC) was performed using Polygram SIL G/UV254 silica gel plates (Macherey-Nagel GmbH & Co. KG, Duren, Germany). Compounds were visualized under UV light and/or by treatment with iodine. Melting points (uncorrected) were determined using a Stuart Scientific SMP30 apparatus.

### General method for the mechanochemical synthesis of unsymmetrical disulfides

A 5 mL ball-mill vessel was charged with 5,5-dimethyl-2-sulfanyl-2-thioxo-1,3,2-dioxaphosphorinane derivative (0.5 mmol, 1 equiv), cesium carbonate (162.91 mg, 0.5 mmol, 1 equiv), and the thiol derivative (0.5 mmol, 1 equiv). A single stainless-steel ball (10 mm diameter) was added, and milling was performed at 30 Hz for 5 min. After milling, the reaction mixture was extracted with dichloromethane (5 mL) and washed with a minimal amount of water (5 mL). The solvent was then evaporated, yielding the pure product. The product’s identity was confirmed through spectroscopic analysis.

## Results and discussion

### Optimization of the process

To develop a new mechanochemical method for synthesizing unsymmetrical disulfides, we began by optimizing the milling and reaction conditions. A series of experiments were conducted to react dodecyl disulfide of phosphorodithioic acid with thiophenol, aiming to form asymmetric dodecyl-phenyl disulfide. During the optimization trials, we tested various parameters, including the number and diameter of grinding balls, reaction time, grinding frequency, and the base employed. The number and diameter of grinding balls were selected so that their total mass corresponded to the mass of one 10 millimeters diameter ball. Our experiments revealed that both the time and frequency of grinding were directly proportional to the reactivity of the substance, with quantitative yields achieved after 10 min of grinding when sodium carbonate was used. In addition, the best results were obtained with a single 10 millimeters diameter ball, and efficiency decreased as the ball diameter was reduced. Furthermore, substituting potassium carbonate for sodium carbonate did not significantly increase the reactivity of the reactants. However, using cesium carbonate reduced the reaction time to 5 min. The reaction was successfully scaled up to produce 5 g of product in a 25 mL vessel containing three 10 mm diameter stainless-steel balls. The product was washed with water and separated. The optimization results are presented in Table 1.

**Table 1:** Optimization of the milling parameters.


Number of balls [-]	Ball diameter [mm]	Time [min]	Frequency [Hz]	Base [-]	Mass of the product [mg]	Yield [%]
1	10	1	30	Na_2_CO_3_	54	35
1	10	3	30	Na_2_CO_3_	112	74
1	10	5	30	Na_2_CO_3_	136	88
1	10	10	30	Na_2_CO_3_	153	99
1	10	15	30	Na_2_CO_3_	152	98
3	7	10	30	Na_2_CO_3_	142	92
8	5	10	30	Na_2_CO_3_	104	67
31	3,2	10	30	Na_2_CO_3_	68	44
1	10	10	25	Na_2_CO_3_	132	85
1	10	10	20	Na_2_CO_3_	89	57
1	10	10	15	Na_2_CO_3_	64	41
1	10	10	30	K_2_CO_3_	153	99
1	10	5	30	K_2_CO_3_	145	93
1	10	10	30	Cs_2_CO_3_	153	99
1	10	5	30	Cs_2_CO_3_	154	99
1	10	3	30	Cs_2_CO_3_	139	90

### Synthesis of unsymmetrical disulfides

After optimizing the milling conditions, we proceeded with the mechanochemical synthesis of a series of asymmetric disulfides. The reactions were completed in a very short time, and no significant increase in vessel temperature was observed during grinding, as measured with an infrared pyrometer. Since all reaction products, except for the unsymmetrical disulfides, existed in salt form, the desired product could be easily isolated by washing the contents of the vessel with water. In cases where the products were oily, extraction with minimal amounts of dichloromethane was used. The results are summarized in Table 2.

**Table 2:** Synthesis of unsymmetrical disulfides.


Entry	*R* ^1^	*R* ^2^	Product	Yield [%]
**1**	C_12_H_25_-	HOC_11_H_23_-	3a	94
**2**	C_12_H_25_-	C_6_H_5_-	3b	99
**3**	C_12_H_25_-	4-MeC_6_H_5_-	3c	98
**4**	C_12_H_25_-	4-NCC_6_H_5_-	3d	92
**5**	C_12_H_25_-	(C_6_H_5_)_3_C-	3e	91
**6**	C_12_H_25_-	2-C_6_H_4_(NH)SC-	3f	89
**7**	C_12_H_25_-	4-MeOC_6_H_5_-	3 g	96
**8**	C_12_H_25_-	4-NO_2_C_6_H_5_-	3 h	94
**9**	C_12_H_25_-	3-NO_2_C_6_H_5_-	3i	95
**10**	C_12_H_25_-	2-NO_2_C_6_H_5_-	3j	92
**11**	C_12_H_25_-	EtO_2_C-CH(-NH_2_)-CH_2_-	3k	95
**12**	C_12_H_25_-	4-MeO_2_CC_6_H_5_-	3 L	96
**13**	C_12_H_25_-	C_6_H_5_CH_2_-	3 m	96
**14**	C_6_H_5_CH_2_-	HOC_11_H_23_-	3n	95
**15**	C_6_H_5_CH_2_-	4-MeC_6_H_5_-	3o	99
**16**	C_6_H_5_CH_2_-	(C_6_H_5_)_3_C-	3p	91
**17**	C_6_H_5_CH_2_-	2-C_6_H_4_(NH)SC-	3q	92
**18**	C_6_H_5_CH_2_-	4-MeOC_6_H_5_-	3r	98
**19**	C_6_H_5_CH_2_-	4-NO_2_C_6_H_5_-	3s	95
**20**	C_6_H_5_CH_2_-	3-NO_2_C_6_H_5_-	3t	92
**21**	C_6_H_5_CH_2_-	2-NO_2_C_6_H_5_-	3u	97
**22**	C_6_H_5_CH_2_-	EtO_2_C-CH(-NH_2_)-CH_2_-	3v	97
**23**	C_6_H_5_CH_2_-	4-NCC_6_H_5_-	3w	96
**24**	4-CH_3_C_6_H_5_-	4-MeOC_6_H_5_	3x	98
**25**	4-CH_3_C_6_H_5_-	(C_6_H_5_)_3_C-	3y	98
**26**	4-CH_3_C_6_H_5_-	2-C_6_H_4_(NH)SC-	3z	96
**27**	4-CH_3_C_6_H_5_-	EtO_2_C-CH(-NH_2_)-CH_2_-	3aa	96
**28**	4-CH_3_C_6_H_5_-	4-MeO_2_CC_6_H_5_-	3ab	98
**29**	C_6_H_5_-	C_6_H_5_CH_2_-	3ac	98
**30**	C_6_H_5_-	4-MeOC_6_H_5_-	3ad	96
**31**	C_6_H_5_-	4-NO_2_C_6_H_5_-	3ae	93
**32**	C_6_H_5_-	4-MeO_2_CC_6_H_5_-	3af	93

In this study, we report a new mechanochemical method for synthesizing unsymmetrical disulfides, including alkyl-alkyl, alkyl-aryl, or aryl-aryl disulfides, using readily accessible 5,5-dimethyl-2-sulfanyl-2-thioxo-1,3,2-dioxaphosphorinane derivatives. The presence of functional groups did not affect the yield of the reaction, except for the -COOH substituent, where even with the use of larger amounts of base and subsequent acidification of the post-reaction mixture, we observed the formation of multiple by-products. The success of the reaction was unaffected by the electron-donor or electron-acceptor effects of substituents in the aromatic ring, regardless of their position, or whether the substrates were aromatic derivatives, highlighting the versatility of the developed procedure. All reactions proceeded quantitatively, and the table shows the yields of product isolation from the reaction mixture. Notably, the formation of symmetric disulfides as by-products was not observed in any case. This is a significant achievement in modern disulfide synthesis, as symmetrization remains a major challenge in many synthetic procedures. Importantly, the synthesis of unsymmetrical disulfides was carried out without the need for any solvent, as even oily products could be easily washed with water and separated on larger scales. Additionally, we successfully replaced triethylamine, traditionally used in conventional synthesis, with the more environmentally friendly cesium carbonate, which could not be used in the conventional process due to solubility issues.

## Conclusions

In this work, we present a new mechanochemical procedure for synthesizing asymmetric disulfides from derivatives of 5,5-dimethyl-2-sulfanyl-2-thioxo-1,3,2-dioxaphosphorinane. We optimized milling parameters such as the number and diameter of milling balls, milling frequency, and reaction time to ensure the best process efficiency.

After optimizing the reaction conditions, we successfully synthesized over 30 unsymmetrical disulfide derivatives with quantitative yields, without the need for solvents. The reactions proceeded rapidly, with no undesired symmetrical products generated. The elimination of solvent usage makes this procedure an excellent adaptation to the principles of modern green chemistry.

The mechanochemical, solvent-free method we developed for synthesizing unsymmetrical disulfides represents a small but significant contribution to meeting the demands of modern green organic chemistry. It is a step toward improving and modernizing the production of substances that can be used to combat various pathogens and facilitate the synthesis of compounds involving the formation of the S-S bond. Our results, combined with recent reports in chemical journals, highlight the growing importance of mechanochemistry in the evolution of modern organic chemistry and highlight the need for continued development of novel synthetic procedures.

## Supplementary Information

Below is the link to the electronic supplementary material.

Supplementary Material 1

## Data Availability

All the data generated or analyzed during this study are included in this published article and its supplementary information files.
