# Supplementary material for: A novel method for the mechanochemical synthesis of unsymmetrical disulfides using phosphorodithioic acid derivatives
Source: Sci Rep. 2025 Dec 5;16:351. doi: 10.1038/s41598-025-29563-5 (PMC12770550; doi:10.1038/s41598-025-29563-5)
Supplement: Supplementary file 1 — Supplementary Material 1 [file 41598_2025_29563_MOESM1_ESM.pdf]

SUPPLEMENTARY INFORMATION

A novel method for the mechanochemical synthesis of unsymmetrical disulfides using phosphorodithioic acid derivatives

Mikołaj Walter, Agata Grobelna, Janusz Rachoń, Dariusz Witt, Sebastian Demkowicz*

Department of Organic Chemistry, Faculty of Chemistry, Gdańsk University of Technology, Narutowicza 11/12, 80-233 Gdansk, Poland, e-mail: sebdemko@pg.gda.pl.

11-(dodecyldisulfanyl)undecan-1-ol 3a

Chromatography: CH_2Cl_2, R_f =0,32, white solid, yield 94%,

^{1}H NMR ^{1}H NMR (500 MHz, cdcl$_3$) δ 3.75 (t, *J* = 6.6 Hz, 2H), 2.69 (t, *J* = 7.4 Hz, 4H), 1.68 (p, *J* = 7.4 Hz, 4H), 1.59 (t, *J* = 6.9 Hz, 2H), 1.38 (q, *J* = 7.1 Hz, 6H), 1.35 – 1.25 (m, 28H), 0.90 (t, *J* = 6.8 Hz, 4H).

^{13}C NMR ^{13}C NMR (126 MHz, cdcl$_3$) δ 77.32, 77.27, 77.06, 76.81, 63.52, 39.19, 33.02, 31.94, 29.68, 29.66, 29.63, 29.61, 29.54, 29.51, 29.49, 29.46, 29.38, 29.27, 29.26, 29.24, 28.56, 28.54, 25.82, 22.72, 14.16.

HRMS (ESI): *m*/*z* $[M + Na]^+$ calcd for $C_{23}H_{48}NaOS_2$: 427.3039; found: 427.3041.

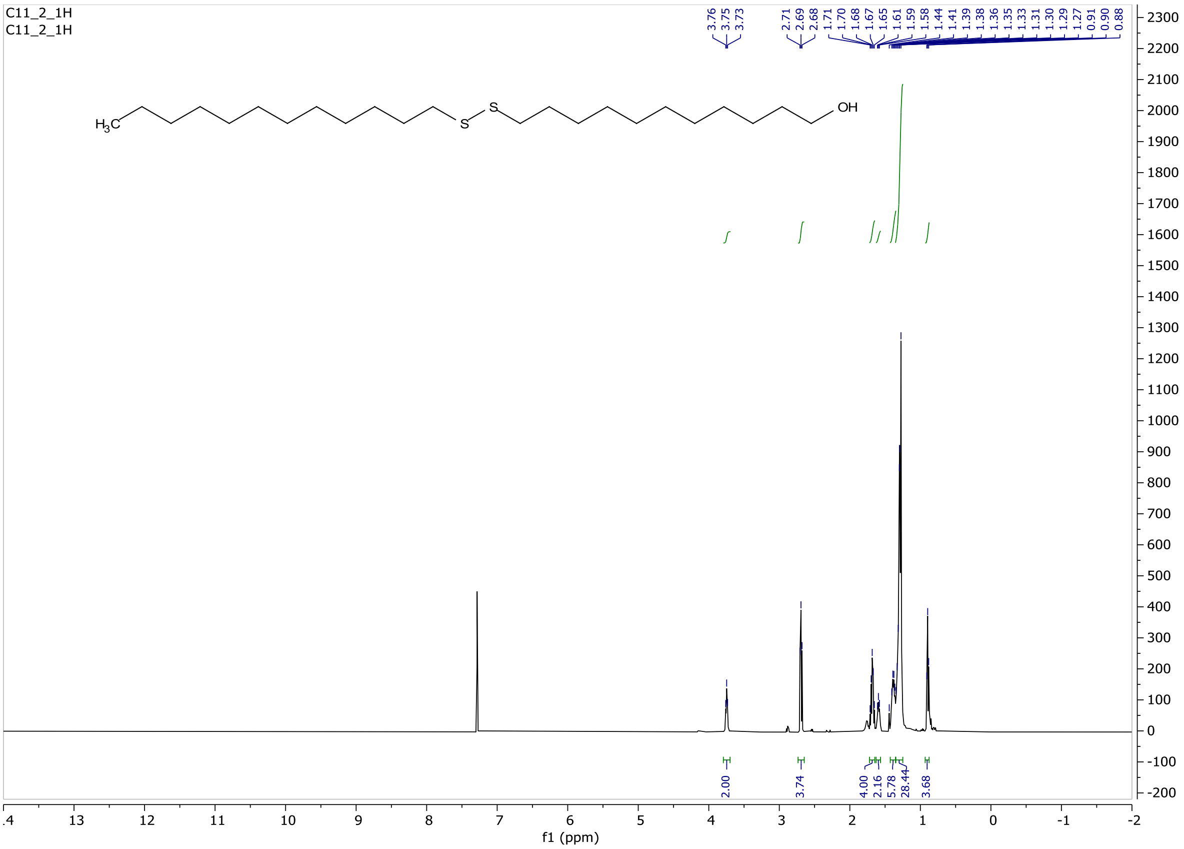

Figure S1. ^{1}H NMR spectrum of 3a

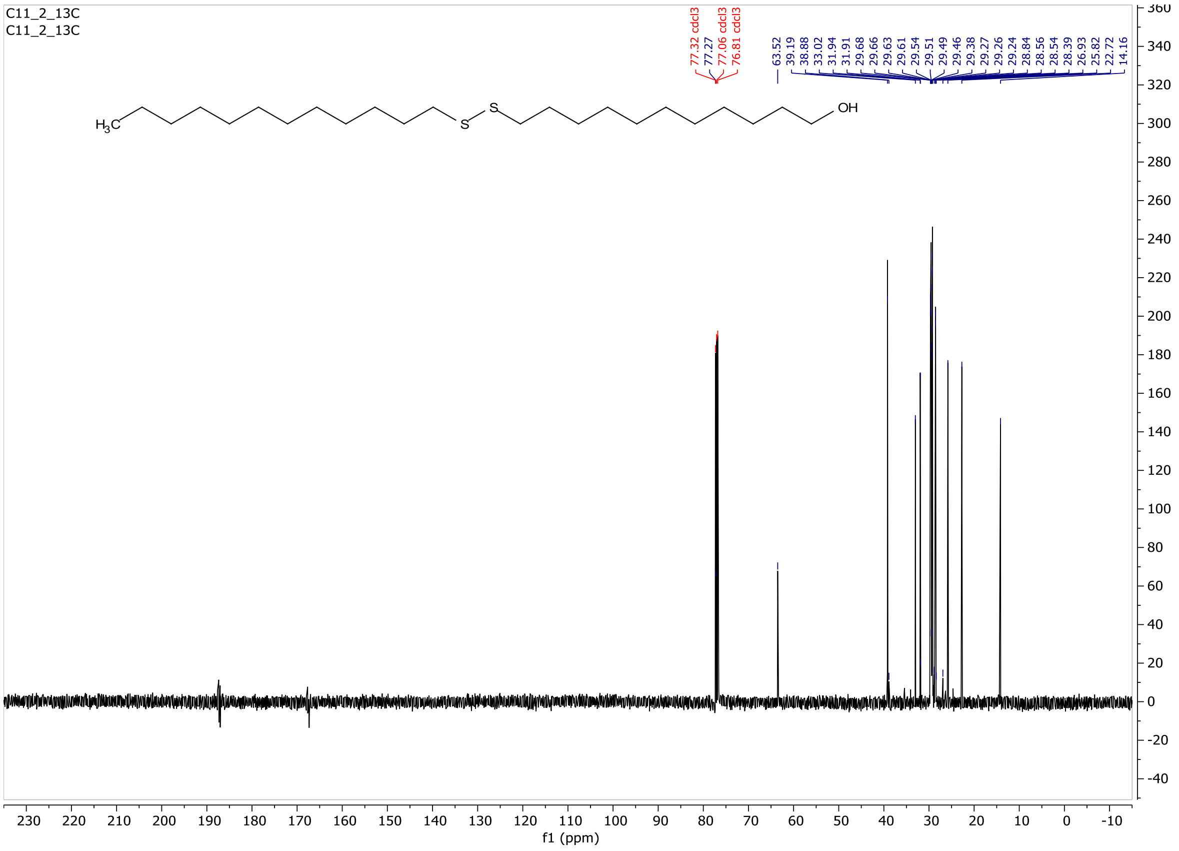

Figure S2. ^{13}C NMR spectrum of 3a

1-dodecyl-2-phenyldisulfane 3b

Chromatography: PE, R_f =0,47, yellowish oil, yield 99%,

^{1}H NMR ^{1}H NMR (500 MHz, cdcl$_3$) δ 7.58 – 7.54 (m, 2H), 7.35 (t, *J* = 7.7 Hz, 2H), 7.24 (td, *J* = 7.2, 1.3 Hz, 1H), 2.76 (t, *J* = 7.3 Hz, 2H), 1.69 (p, *J* = 7.4 Hz, 2H), 1.38 (t, *J* = 7.4 Hz, 2H), 1.36 – 1.21 (m, 17H), 0.91 (t, *J* = 6.9 Hz, 3H).

^{13}C NMR ^{13}C NMR (126 MHz, cdcl$_3$) δ 137.71, 128.94, 127.39, 126.64, 77.30, 77.05, 76.79, 39.01, 31.95, 29.67, 29.66, 29.60, 29.50, 29.38, 29.20, 28.81, 28.48, 22.73, 14.17.

HRMS (ESI): *m*/*z* [M + Na]$^+$ calcd for $C_{18}H_{30}NaS_2$: 333.1681; found: 333.1678

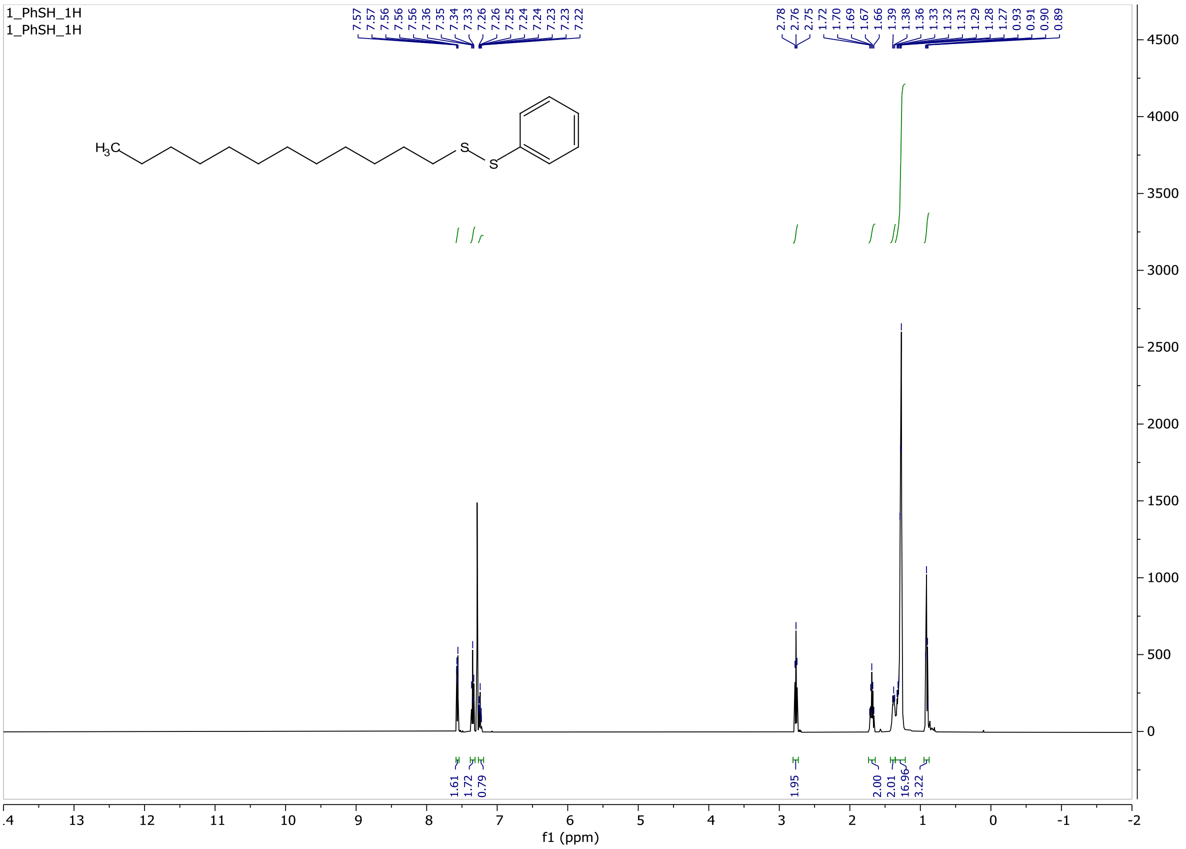

Figure S3. ^{1}H NMR spectrum of 3b

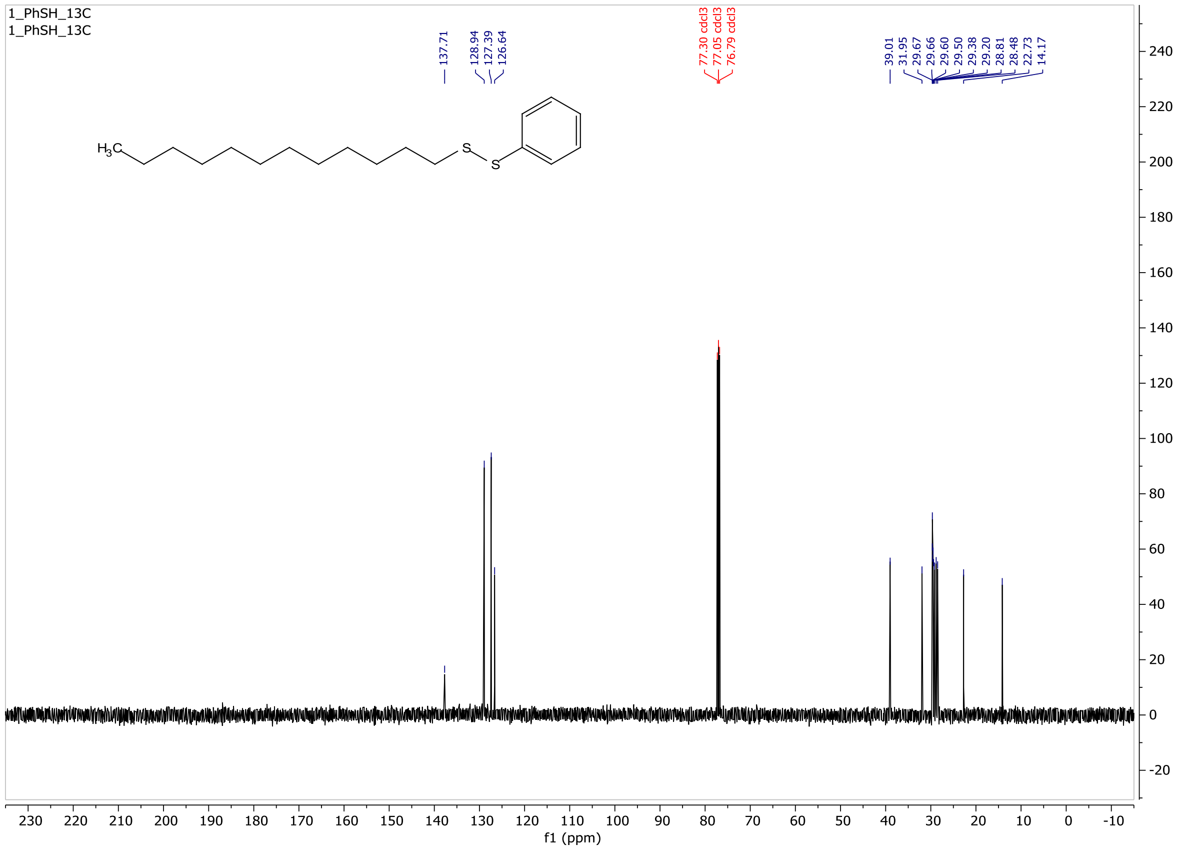

Figure S4. ^{13}C NMR spectrum of 3b

1-dodecyl-2-(p-tolyl)disulfane 3c

Chromatography: PE, R_f=0,45, yellowish oil, yield 98%,

^{1}H NMR ^{1}H NMR (500 MHz, cdcl$_3$) δ 7.49 – 7.44 (m, 2H), 7.16 (d, *J* = 7.9 Hz, 2H), 2.75 (t, *J* = 7.3 Hz, 2H), 2.37 (s, 3H), 1.69 (p, *J* = 7.3 Hz, 2H), 1.37 (t, *J* = 7.5 Hz, 3H), 1.34 – 1.23 (m, 15H), 0.92 (t, *J* = 7.0 Hz, 3H).

^{13}C NMR ^{13}C NMR (126 MHz, cdcl$_3$) δ 136.94, 134.26, 129.73, 128.53, 128.35, 77.31, 77.26, 77.06, 76.80, 38.95, 31.96, 29.69, 29.63, 29.52, 29.46, 29.40, 29.22, 28.78, 28.50, 22.74, 21.06, 14.17.

HRMS (ESI): *m/z* [M + Na]$^+$ calcd for $C_{19}H_{32}NaS_2$: 347.1838; found: 347.1840

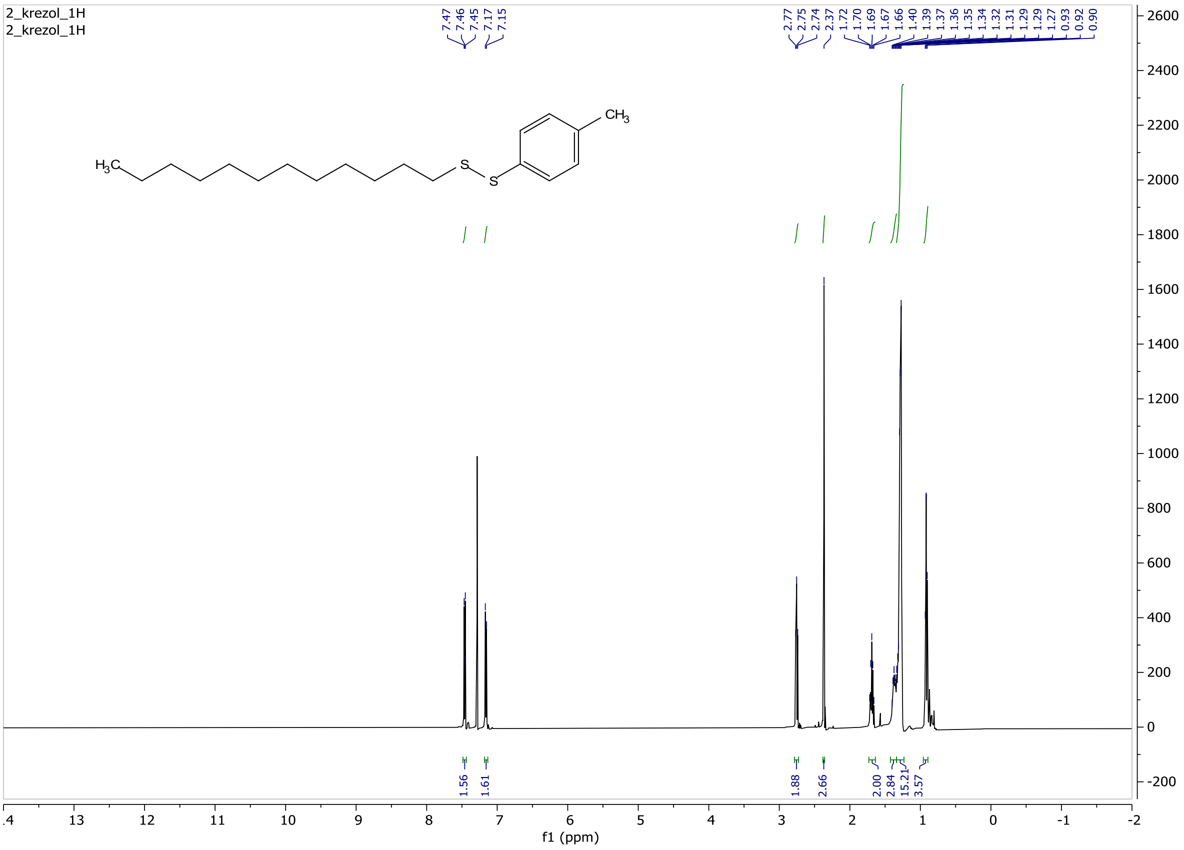

Figure S5. ^{1}H NMR spectrum of 3c

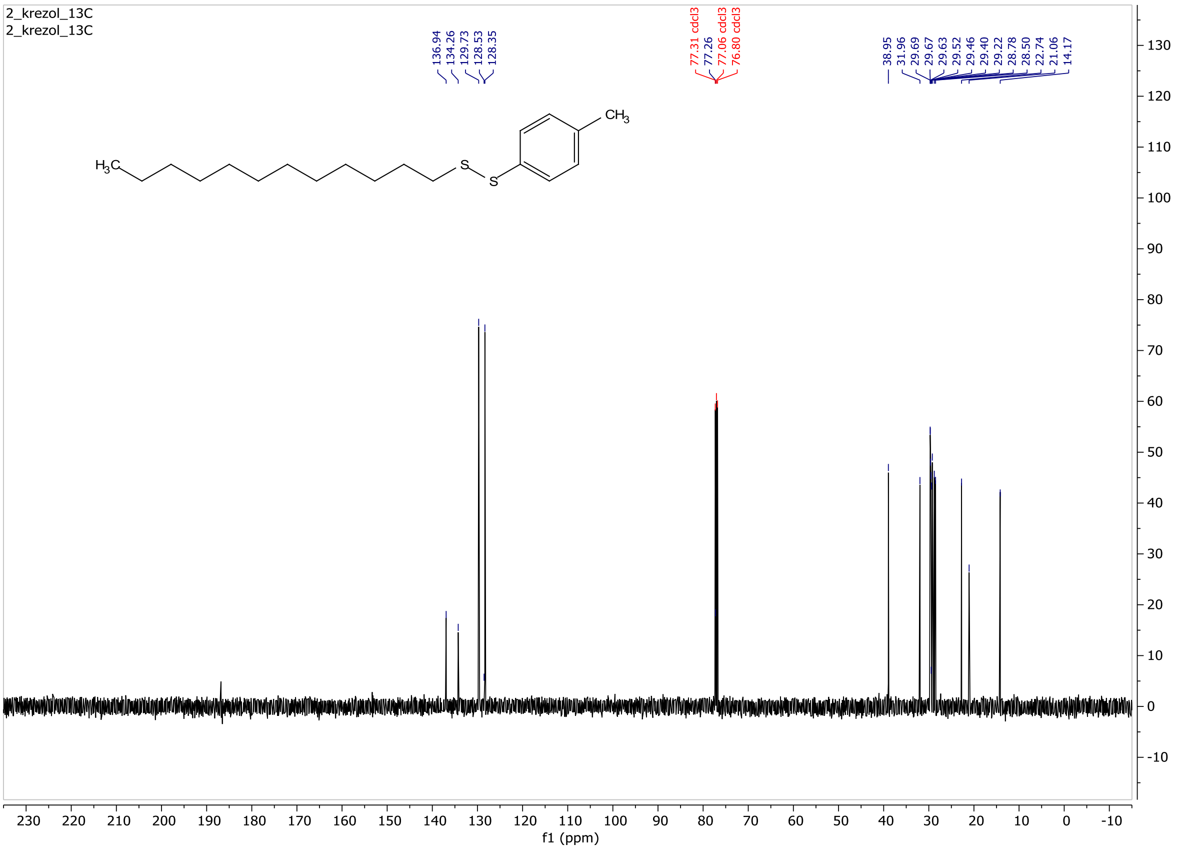

Figure S6. ^{13}C NMR spectrum of 3c

4-((dodecyldisulfanyl)methyl)benzonitrile 3d

Chromatography: PE, R_f =0,38, yellowish oil, yield 92%,

^{1}H NMR ^{1}H NMR (500 MHz, cdcl$_3$) δ 7.63 (d, *J* = 8.2 Hz, 2H), 7.45 (d, *J* = 8.1 Hz, 2H), 3.89 (s, 2H), 2.45 – 2.39 (m, 2H), 1.56 (t, *J* = 7.3 Hz, 2H), 1.27 (s, 18H), 0.90 (t, *J* = 6.8 Hz, 3H).

^{13}C NMR ^{13}C NMR (126 MHz, cdcl$_3$) δ 143.43, 132.25, 130.03, 129.51, 118.76, 111.17, 77.34, 77.29, 77.08, 76.83, 42.97, 38.79, 31.94, 29.68, 29.61, 29.50, 29.38, 29.19, 29.08, 28.46, 22.72, 14.17.

HRMS (ESI): *m*/*z* [M + H]$^+$ calcd for $C_{20}H_{32}NS_2$: 350.1976; found: 350.1979

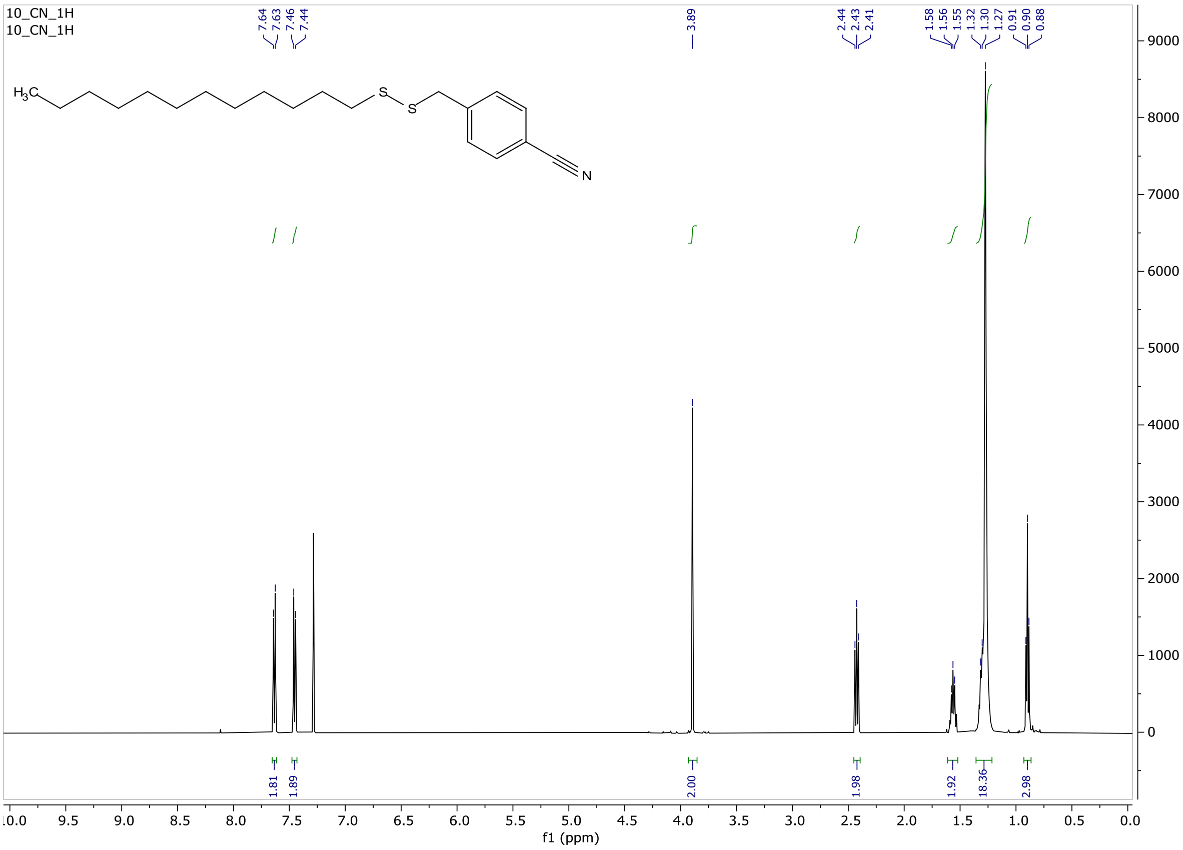

Figure S7. ^{1}H NMR spectrum of 3d

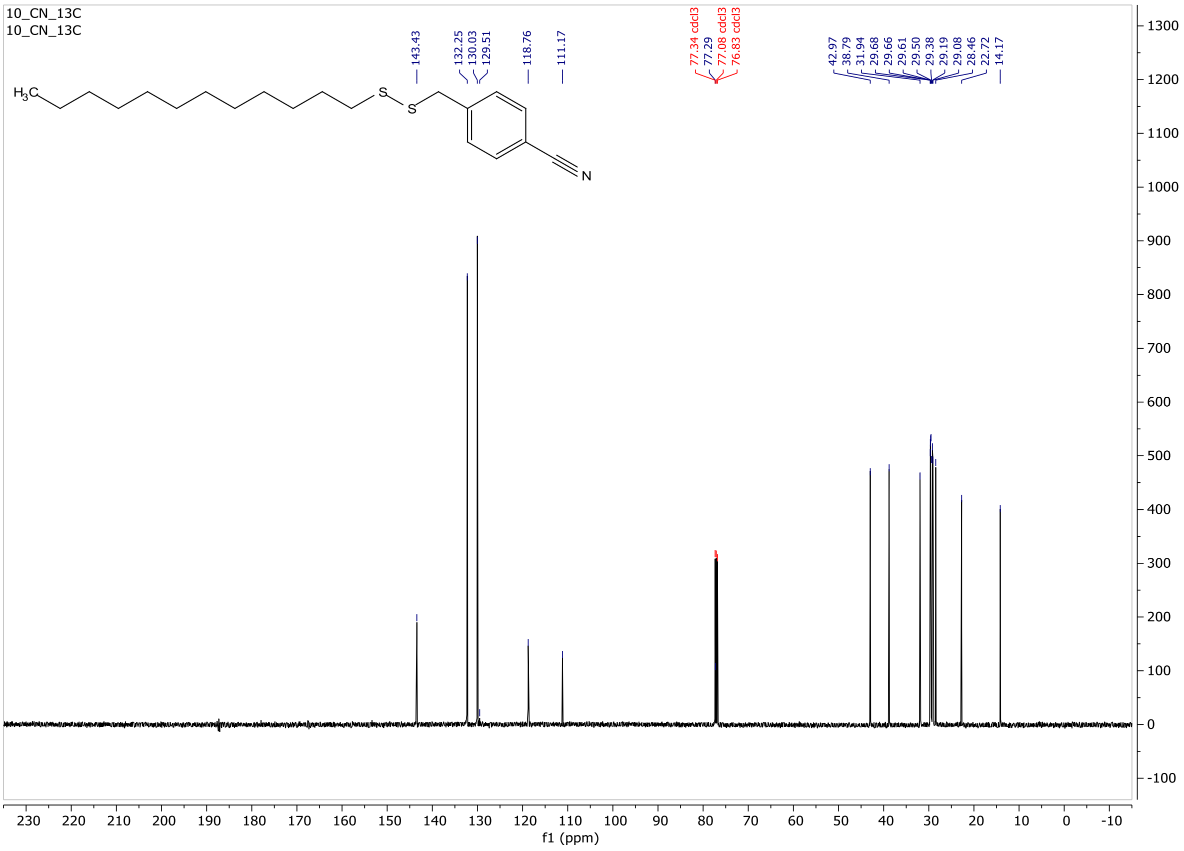

Figure S8. ^{13}C NMR spectrum of 3d

1-dodecyl-2-trityldisulfane 3e

Chromatography: PE, R_f =0,48, yellowish oil, yield 91%,

^{1}H NMR ^{1}H NMR (500 MHz, dmso) δ 7.87 (s, 1H), 7.34 (d, *J* = 7.3 Hz, 4H), 7.26 – 7.14 (m, 10H), 3.83 (d, *J* = 15.4 Hz, 2H), 1.29 – 1.09 (m, 16H), 0.92 (s, 4H), 0.81 (t, *J* = 6.8 Hz, 3H). **^{13}C NMR** ^{13}C NMR (126 MHz, dmso) δ 148.60, 148.54, 147.19, 143.84, 130.04, 129.29, 127.92, 127.90, 127.02, 126.96, 78.93, 78.87, 78.67, 78.41, 74.07, 70.81, 40.43, 40.26, 40.09, 39.92, 39.76, 38.45, 36.45, 31.81, 29.52, 29.47, 29.43, 29.32, 29.24, 29.04, 28.94, 28.74, 28.27, 22.61, 22.29, 14.28.

HRMS (ESI): *m*/*z* $[M + Na]^+$ calcd for $C_{31}H_{40}NaS_2$: 499.2464; found: 499.2463.

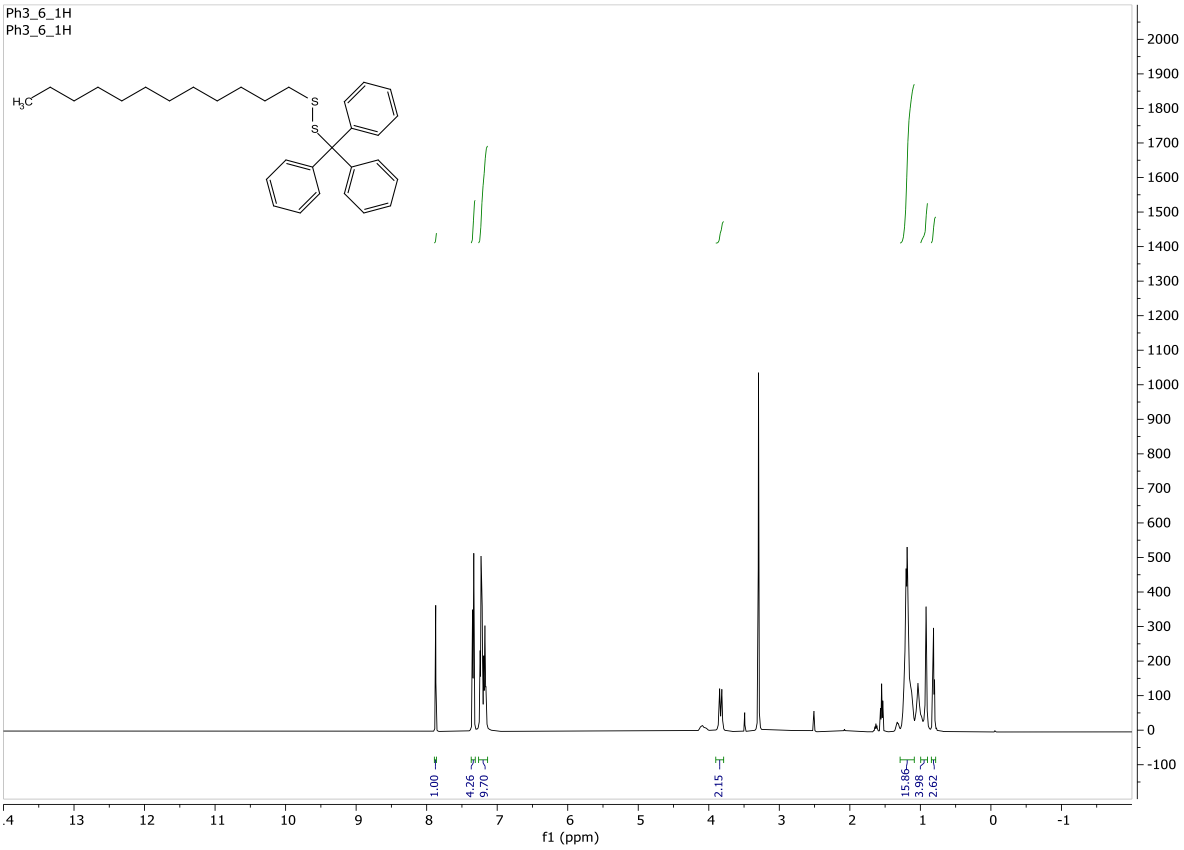

Figure S9. ^{1}H NMR spectrum of 3e

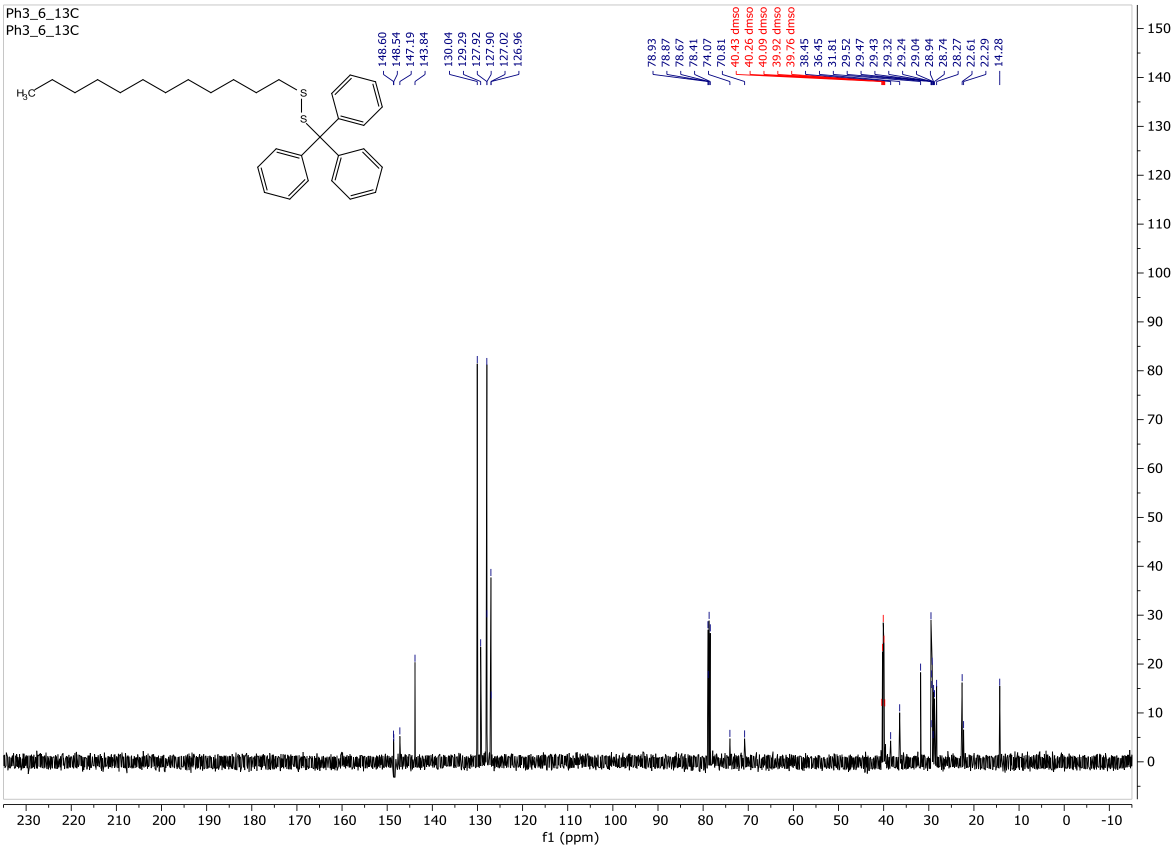

Figure S10. ^{13}C NMR spectrum of 3e

2-(dodecyldisulfanyl)benzo[d]thiazole 3f

Chromatography: PE:CH_2Cl_2 (2:1), R_f =0,37, yellowish oil, yield 89%,

^{1}H NMR ^{1}H NMR (500 MHz, cdcl$_3$) δ 7.88 (d, *J* = 8.1 Hz, 1H), 7.82 (d, *J* = 8.0 Hz, 1H), 7.45 (tt, *J* = 8.3, 1.2 Hz, 1H), 7.38 – 7.31 (m, 1H), 2.97 (t, *J* = 7.4 Hz, 2H), 1.77 (p, *J* = 7.4 Hz, 2H), 1.43 (p, *J* = 7.0 Hz, 2H), 1.27 (d, *J* = 9.2 Hz, 17H), 0.89 (t, *J* = 6.9 Hz, 3H).

^{13}C NMR. ^{13}C NMR (126 MHz, cdcl$_3$) δ 173.33, 155.18, 135.86, 126.23, 124.52, 122.12, 121.13, 77.30, 77.05, 76.79, 39.61, 31.93, 29.63, 29.57, 29.47, 29.36, 29.15, 28.99, 28.45, 22.71, 14.16.

HRMS (ESI): *m*/*z* [M + H]$^+$ calcd for $C_{19}H_{30}NS_3$: 368.1535; found: 368.1536.

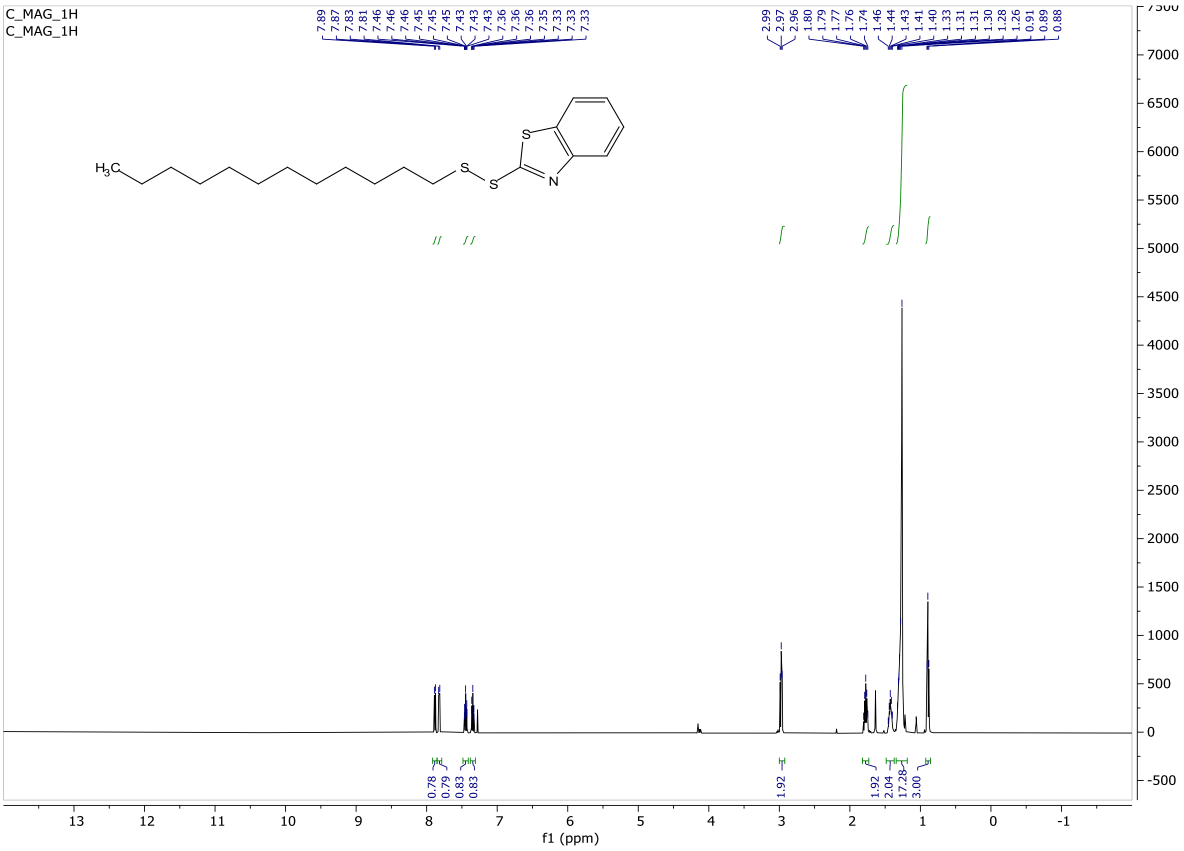

Figure S11. ^{1}H NMR spectrum of 3f

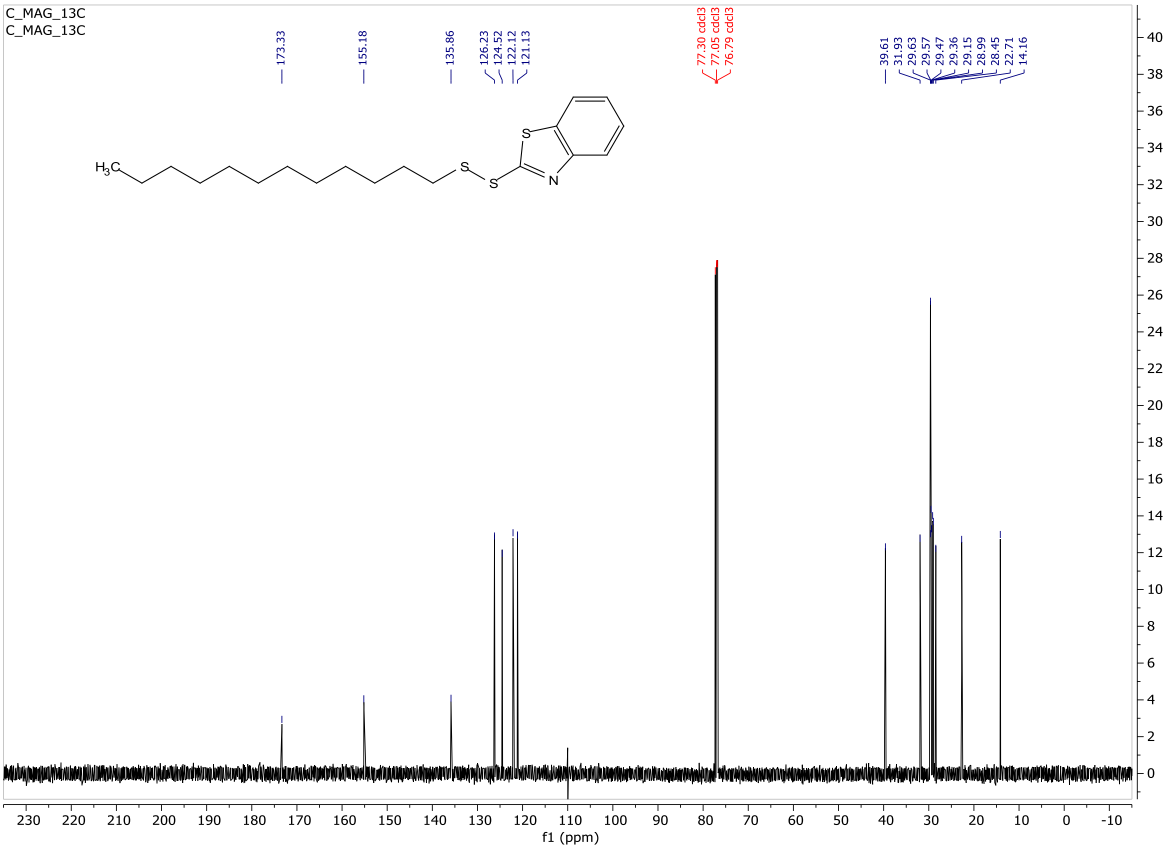

Figure S12. ^{13}C NMR spectrum of 3f

1-dodecyl-2-(4-methoxybenzyl)disulfane 3g

Chromatography: PE, R_f =0,32, yellowish oil, yield 96%,

^{1}H NMR ^{1}H NMR (500 MHz, cdcl$_3$) δ 7.25 (d, *J* = 8.5 Hz, 2H), 6.88 – 6.80 (m, 2H), 3.85 (s, 2H), 3.80 (s, 3H), 2.43 (t, *J* = 7.4 Hz, 2H), 1.61 – 1.51 (m, 2H), 1.34 – 1.19 (m, 20H), 0.88 (t, *J* = 6.9 Hz, 3H).

^{13}C NMR ^{13}C NMR (126 MHz, cdcl$_3$) δ 158.98, 153.20, 130.40, 129.55, 113.88, 77.28, 77.03, 76.77, 55.27, 43.15, 38.76, 31.93, 29.67, 29.60, 29.52, 29.36, 29.20, 29.08, 28.50, 22.70, 14.14.

HRMS (ESI): *m*/*z* $[M + Na]^+$ calcd for $C_{20}H_{34}NaOS_2$: 377.1943; found: 377.1946.

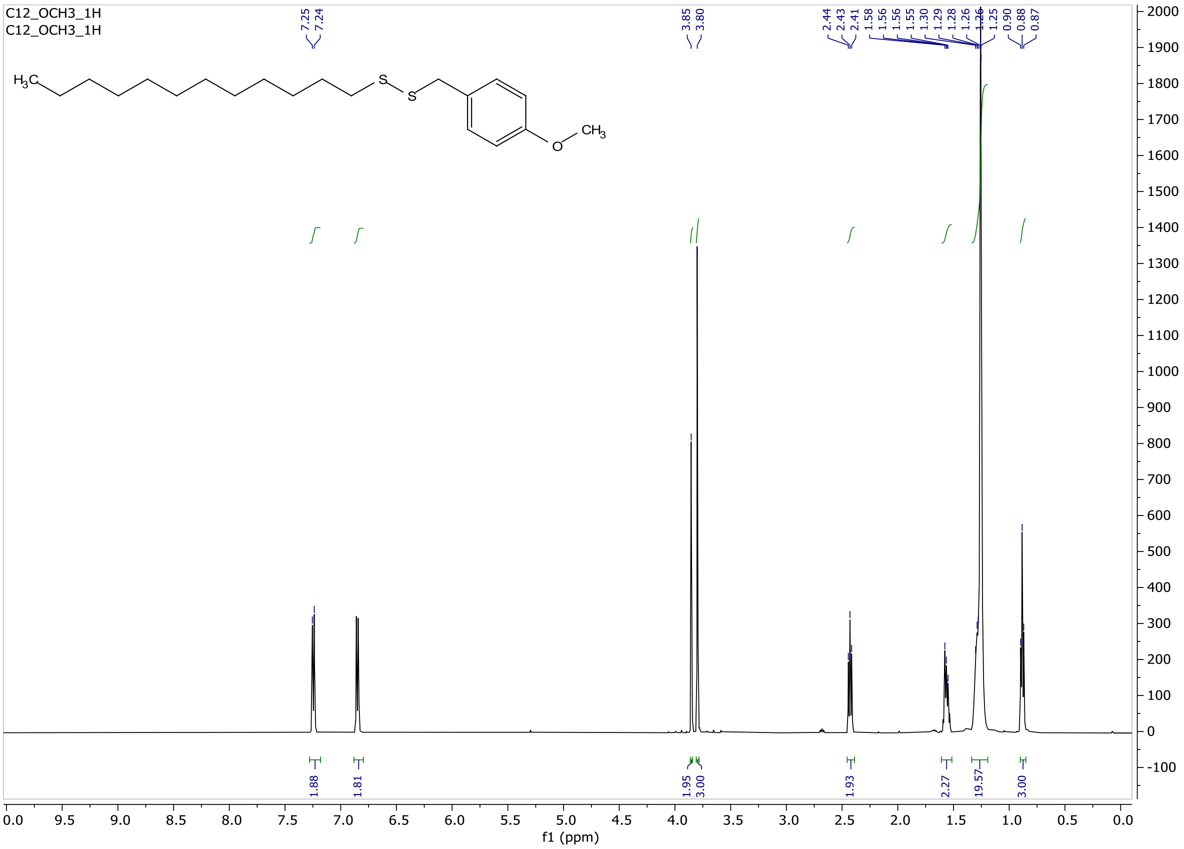

Figure S13. ^{1}H NMR spectrum of 3g

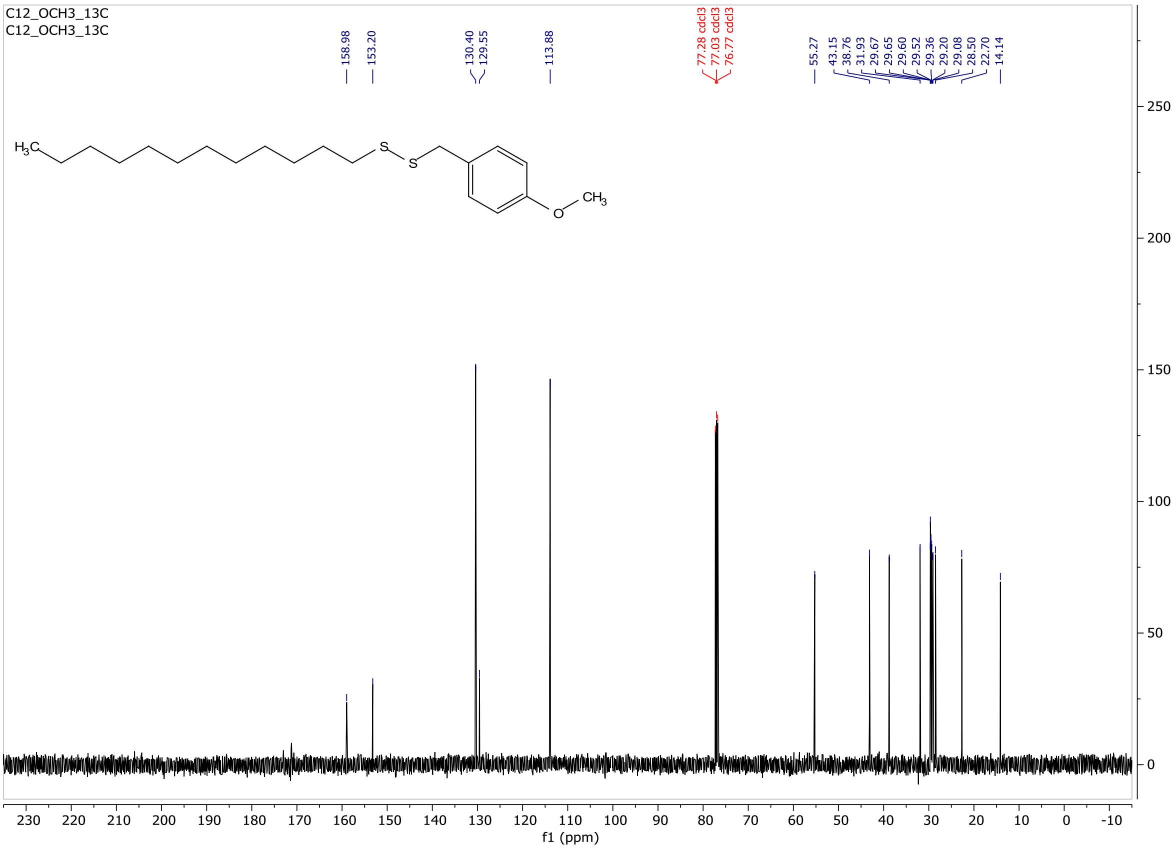

Figure S14. ^{13}C NMR spectrum of 3g

1-dodecyl-2-(4-nitrobenzyl)disulfane 3h

Chromatography: PE, R_f =0,41, yellowish oil, yield 94%,

^{1}H NMR ^{1}H NMR (500 MHz, cdcl$_3$) δ 8.25 – 8.18 (m, 2H), 7.54 – 7.49 (m, 2H), 3.94 (s, 2H), 2.45 (t, J = 7.4 Hz, 2H), 1.58 (p, J = 7.3 Hz, 2H), 1.35 – 1.23 (m, 19H), 0.90 (t, J = 6.9 Hz, 3H). **^{13}C NMR** ^{13}C NMR (126 MHz, cdcl$_3$) δ 147.19, 145.55, 130.10, 123.72, 77.31, 77.26, 77.06, 76.80, 42.60, 38.82, 31.94, 29.67, 29.59, 29.50, 29.44, 29.38, 29.19, 29.09, 28.45, 22.72, 14.16.

HRMS (ESI): m/z [M + Na]$^+$ calcd for $C_{19}H_{31}NNaO_2S_2$: 392.1688; found: 392.1685.

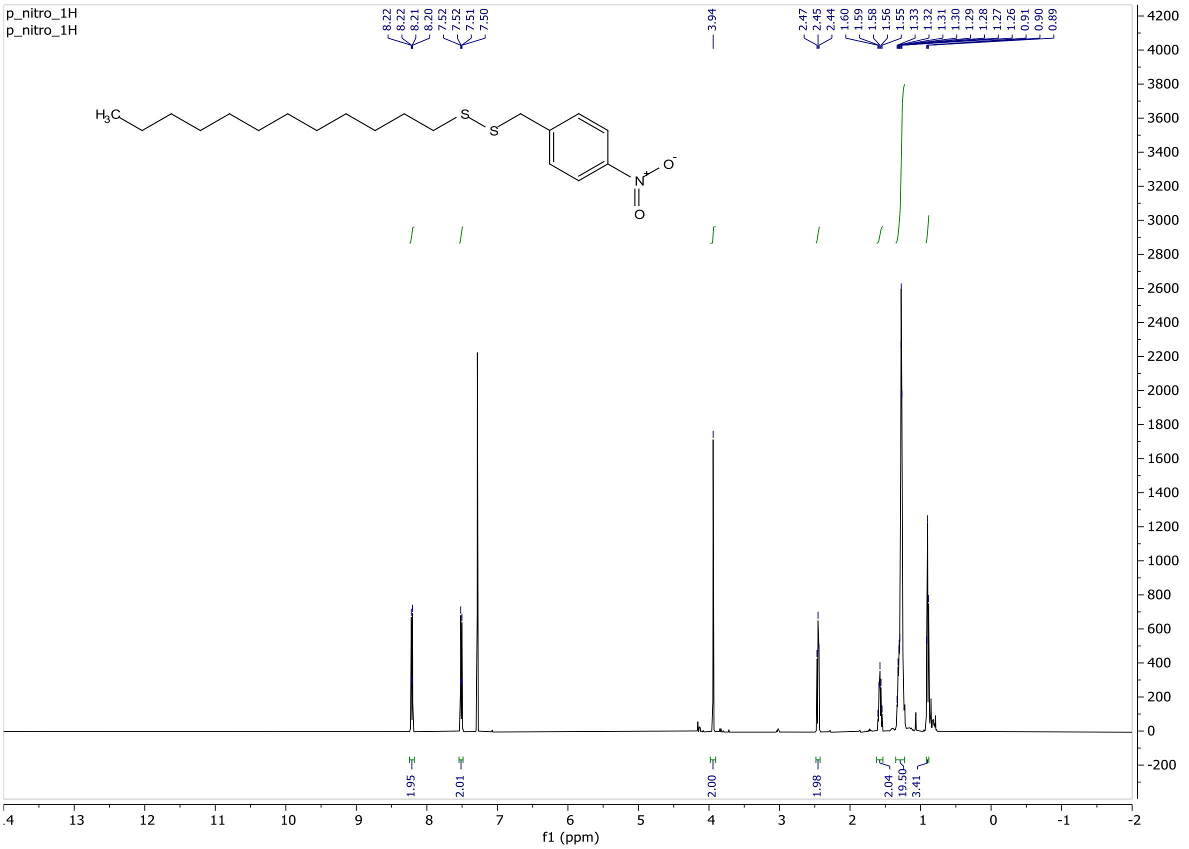

Figure S15. ^{1}H NMR spectrum of 3h

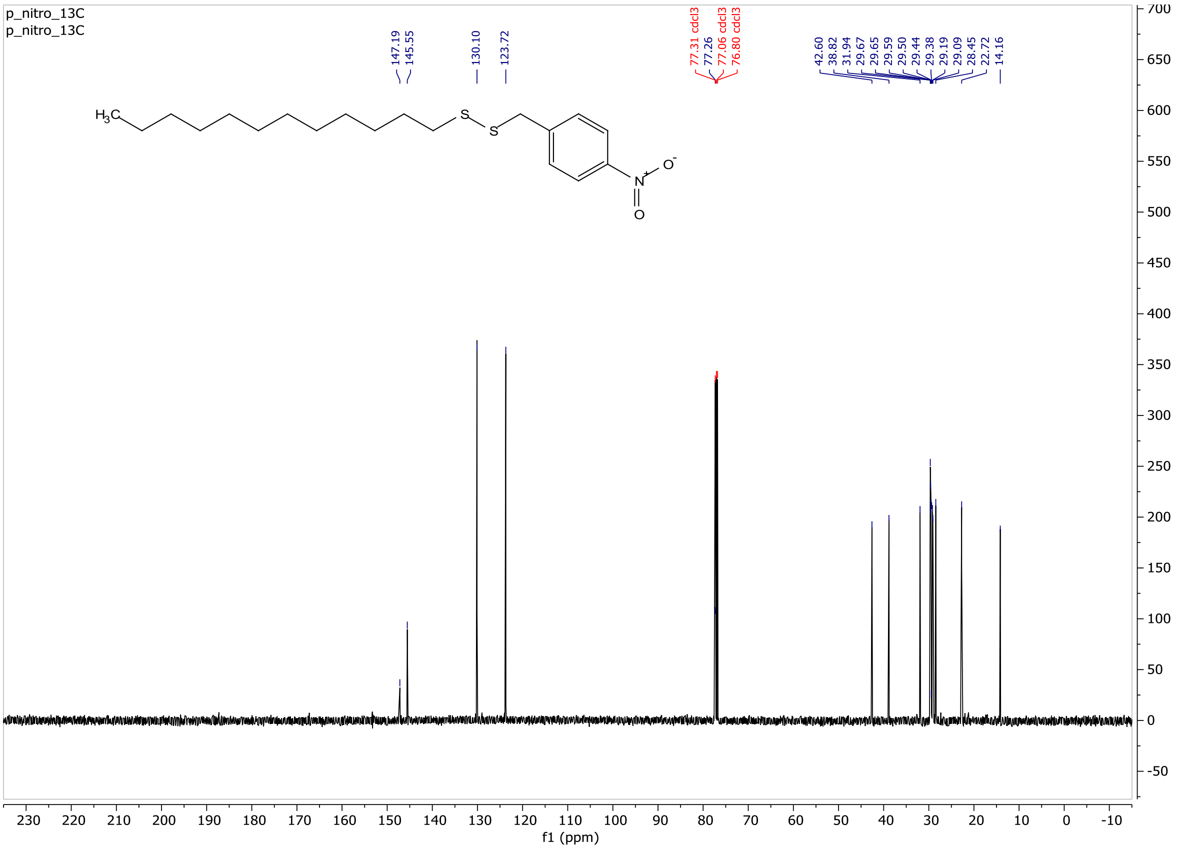

Figure S16. ^{13}C NMR spectrum of 3h

1-dodecyl-2-(3-nitrobenzyl)disulfane 3i

Chromatography: PE, R_f=0,41, yellowish oil, yield 95%,

^{1}H NMR ^{1}H NMR (500 MHz, cdcl$_3$) δ 8.19 (d, *J* = 2.2 Hz, 1H), 8.14 (dd, *J* = 8.2, 1.2 Hz, 1H), 7.66 (dd, *J* = 7.8, 1.7 Hz, 1H), 7.50 (t, *J* = 7.9 Hz, 1H), 3.93 (s, 2H), 2.44 (t, *J* = 7.4 Hz, 2H), 1.56 (dd, *J* = 14.2, 6.8 Hz, 2H), 1.42 – 1.16 (m, 19H), 0.87 (t, *J* = 6.8 Hz, 3H).

^{13}C NMR ^{13}C NMR (126 MHz, cdcl$_3$) δ 139.97, 135.36, 129.39, 124.15, 122.37, 77.29, 77.03, 76.78, 42.39, 38.77, 31.92, 29.63, 29.57, 29.47, 29.35, 29.16, 29.10, 28.46, 22.69, 14.13.

HRMS (ESI): *m*/*z* [M + Na]$^+$ calcd for $C_{19}H_{31}NNaO_2S_2$: 392.1688; found: 392.1690.

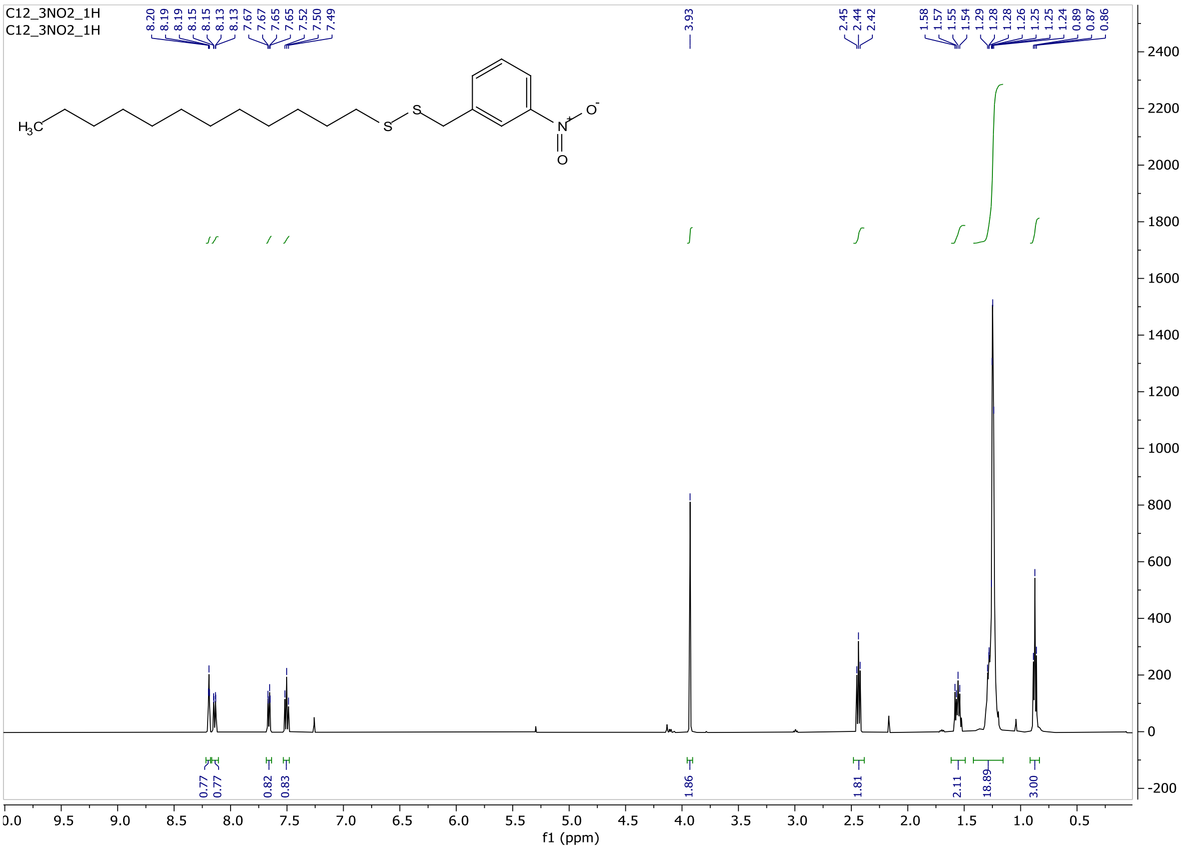

Figure S17. ^{1}H NMR spectrum of 3i

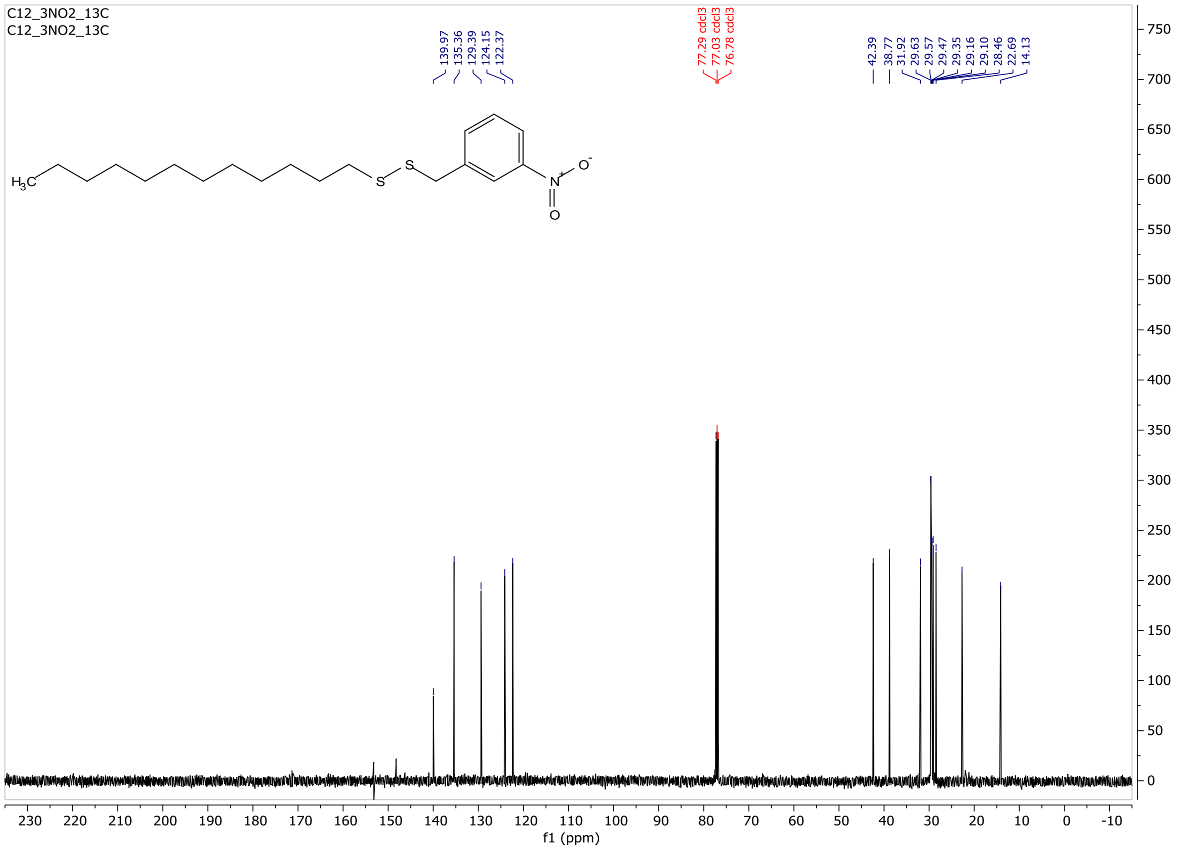

Figure S18. ^{13}C NMR spectrum of 3i

1-dodecyl-2-(2-nitrobenzyl)disulfane 3j

Chromatography: PE, R_f=0,41, yellowish oil, yield 92%,

^{1}H NMR ^{1}H NMR (500 MHz, cdcl$_3$) δ 8.04 (dd, J = 8.1, 1.4 Hz, 1H), 7.57 (td, J = 7.5, 1.3 Hz, 1H), 7.50 – 7.43 (m, 2H), 4.24 (s, 2H), 2.41 – 2.33 (m, 2H), 1.52 (p, J = 7.2 Hz, 2H), 1.24 (d, J = 6.7 Hz, 19H), 0.88 (t, J = 6.9 Hz, 3H).

^{13}C NMR ^{13}C NMR (126 MHz, cdcl$_3$) δ 153.20, 133.72, 133.11, 132.75, 128.47, 125.43, 77.28, 77.02, 76.77, 40.88, 38.80, 31.92, 29.64, 29.58, 29.47, 29.35, 29.14, 28.93, 28.47, 22.69, 14.13.

HRMS (ESI): m/z [M + Na]$^+$ calcd for $C_{19}H_{31}NNaO_2S_2$: 392.1688; found: 392.1691.

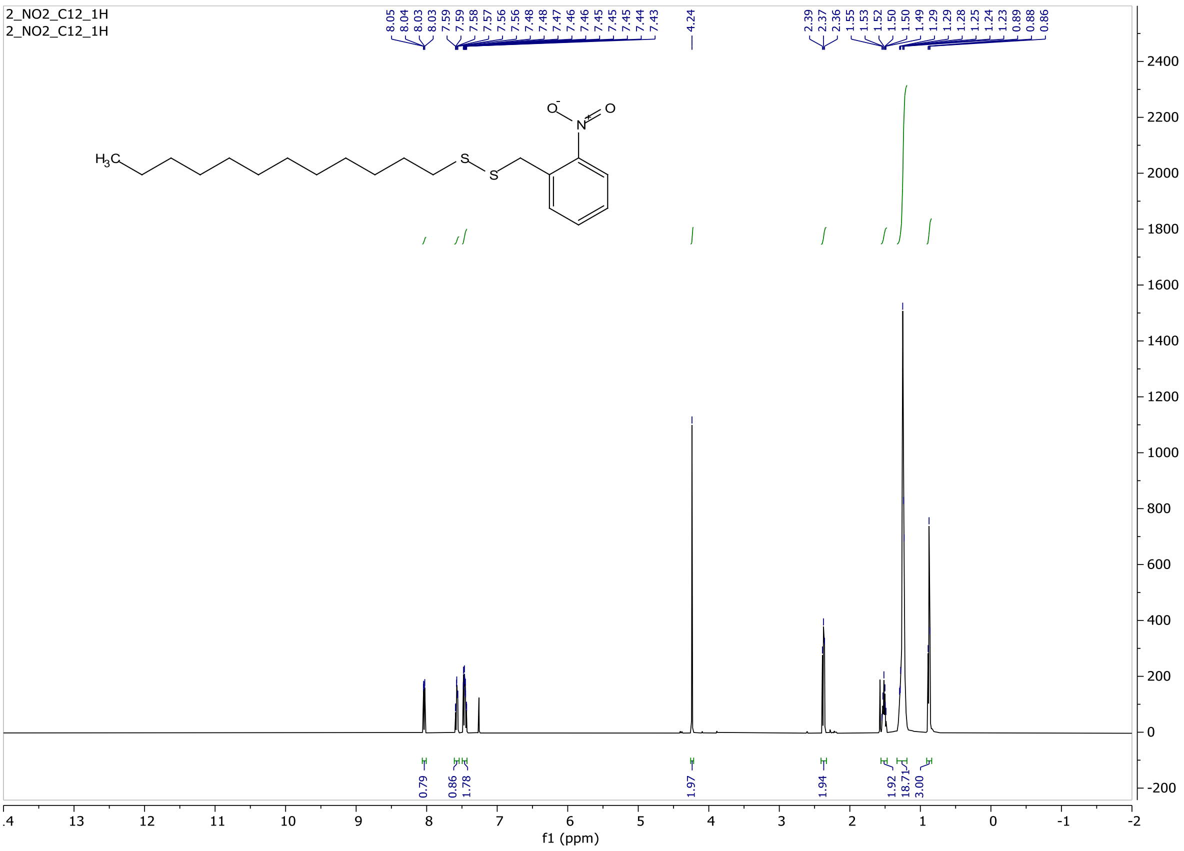

Figure S19. ^{1}H NMR spectrum of 3j

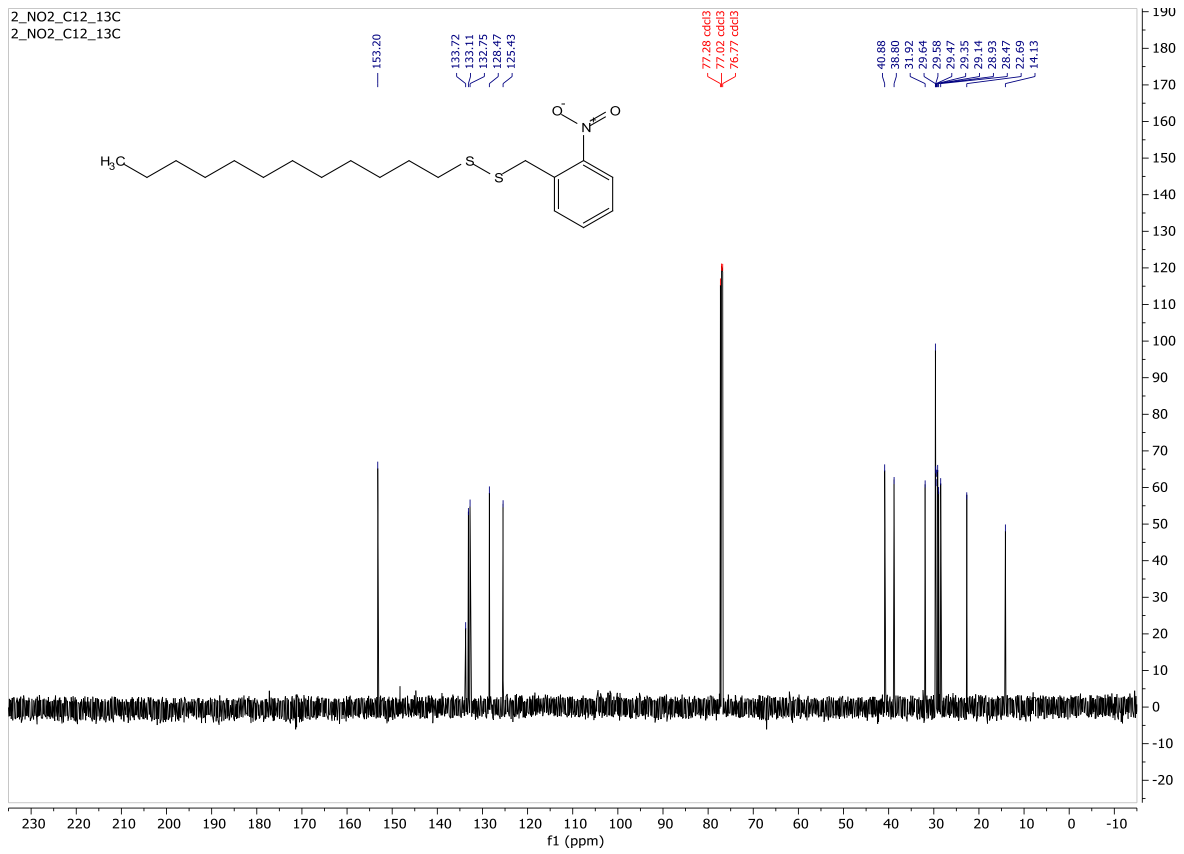

Figure S20. ^{13}**C NMR spectrum of 3j**

ethyl 2-amino-3-(dodecyldisulfanyl)propanoate 3k

Chromatography: CH_2CL_2, R_f =0,28, yellowish oil, yield 95%,

^{1}H NMR ^{1}H NMR (500 MHz, cdcl$_3$) δ 4.22 (q, *J* = 7.1 Hz, 2H), 3.80 (dd, *J* = 7.8, 4.6 Hz, 1H), 3.11 (dd, *J* = 13.6, 4.5 Hz, 1H), 2.88 (dd, *J* = 13.5, 7.8 Hz, 1H), 2.72 (t, *J* = 7.4 Hz, 2H), 1.68 (p, *J* = 7.4 Hz, 2H), 1.41 – 1.35 (m, 2H), 1.32 – 1.24 (m, 24H), 0.89 (t, *J* = 6.8 Hz, 4H).

^{13}C NMR ^{13}C NMR (126 MHz, cdcl$_3$) δ 77.31, 77.05, 76.80, 61.34, 53.62, 43.70, 38.93, 31.92, 29.65, 29.64, 29.60, 29.51, 29.36, 29.24, 29.11, 28.51, 22.70, 14.20, 14.14.

HRMS (ESI): *m*/*z* $[M + H]^+$ calcd for $C_{17}H_{36}NOS_2$: 350.2187; found: 350.2189.

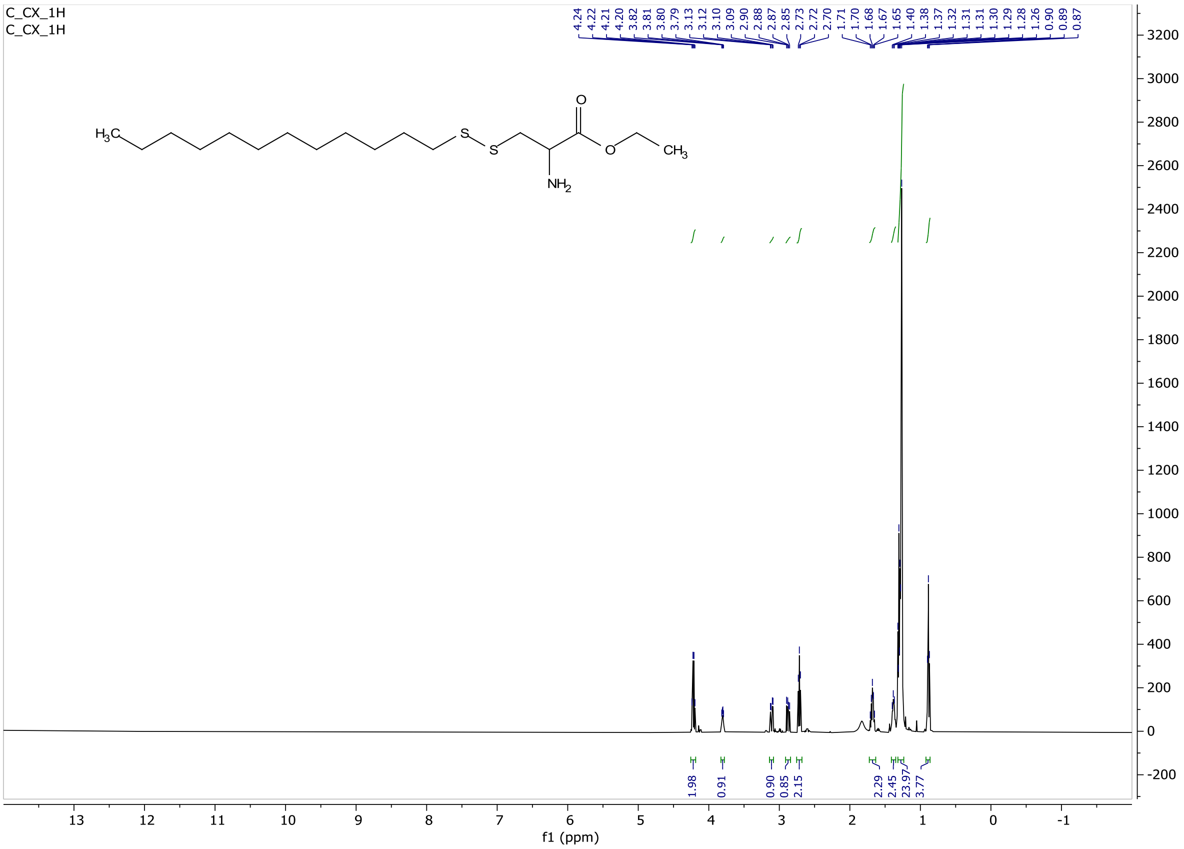

Figure S21. ^{1}H NMR spectrum of 3k

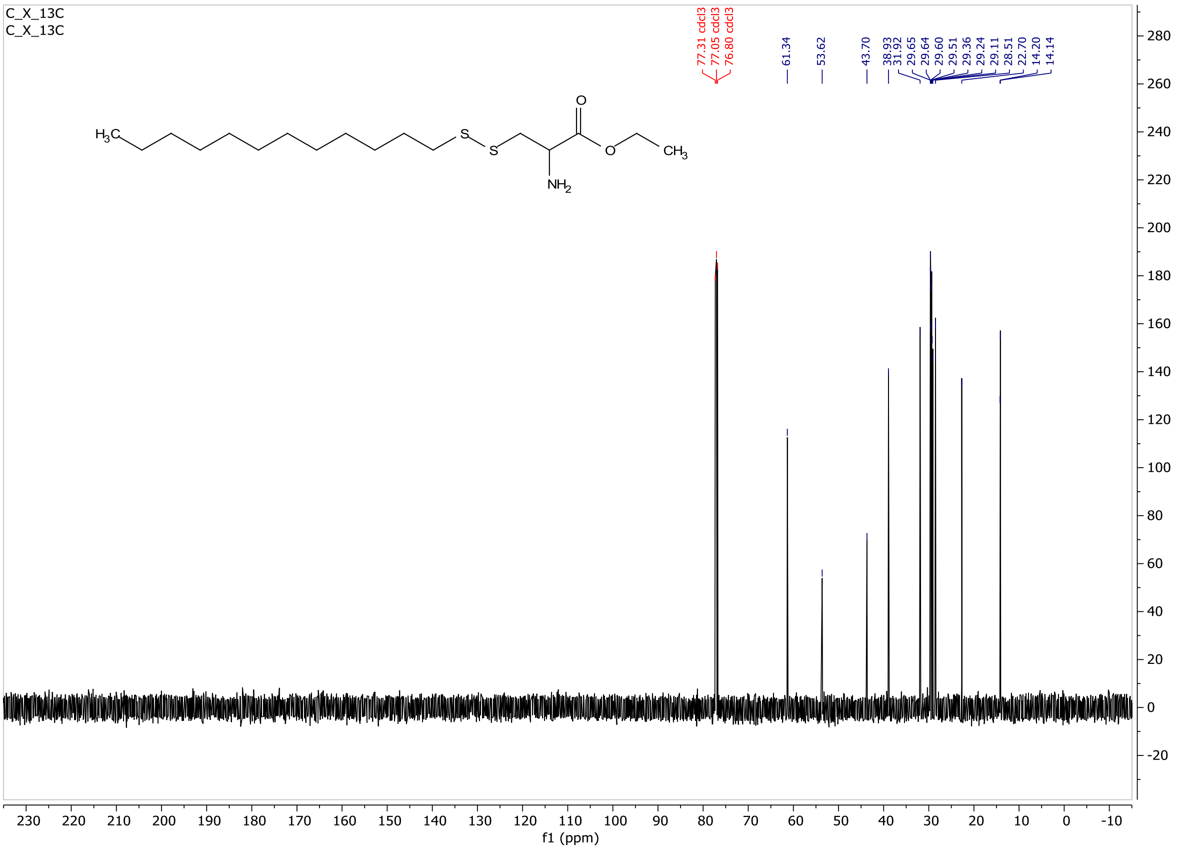

Figure S22. ^{13}C **NMR spectrum of 3k**

methyl 4-(dodecyldisulfanyl)benzoate 3l

Chromatography: PE, R_f =0,30, yellowish oil, yield 92%,

^{1}H NMR ^{1}H NMR (500 MHz, cdcl$_3$) δ 7.99 (d, *J* = 8.5 Hz, 2H), 7.60 (d, *J* = 8.5 Hz, 2H), 3.93 (s, 3H), 2.76 (t, *J* = 7.3 Hz, 2H), 1.67 (dd, *J* = 8.4, 6.6 Hz, 2H), 1.37 (p, *J* = 7.2 Hz, 3H), 1.34 – 1.24 (m, 21H), 0.90 (t, *J* = 6.9 Hz, 3H).

^{13}C NMR ^{13}C NMR (126 MHz, cdcl$_3$) δ 144.16, 130.25, 130.03, 129.50, 128.11, 127.93, 125.60, 77.61, 77.54, 77.32, 77.27, 77.07, 76.81, 52.13, 52.11, 39.03, 38.66, 32.72, 32.67, 31.94, 29.67, 29.65, 29.60, 29.58, 29.48, 29.37, 29.17, 28.89, 28.80, 28.46, 28.44, 22.72, 22.00, 21.20, 14.16,

HRMS (ESI): *m*/*z* [M + H]$^+$ calcd for $C_{20}H_{33}O_2S_2$: 369.1916; found: 369.1917.

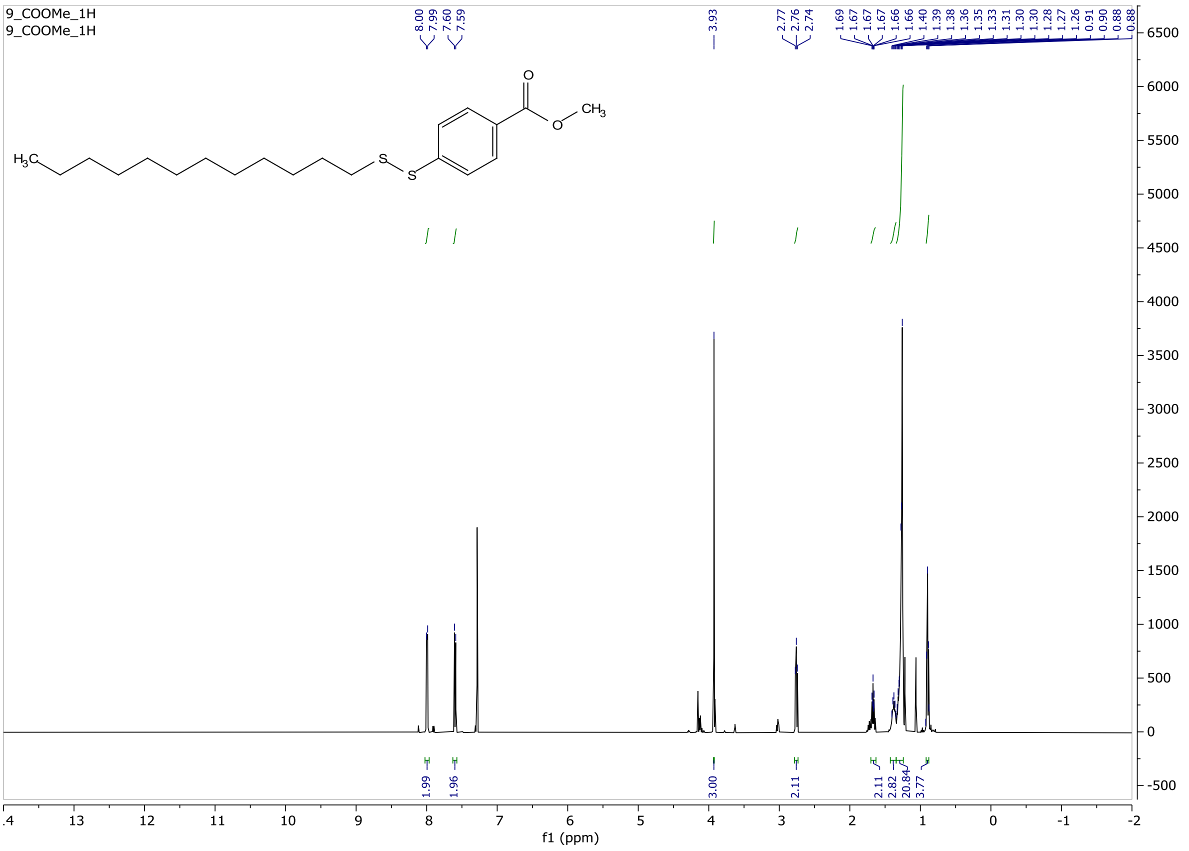

Figure S23. ^{1}H NMR spectrum of 3l

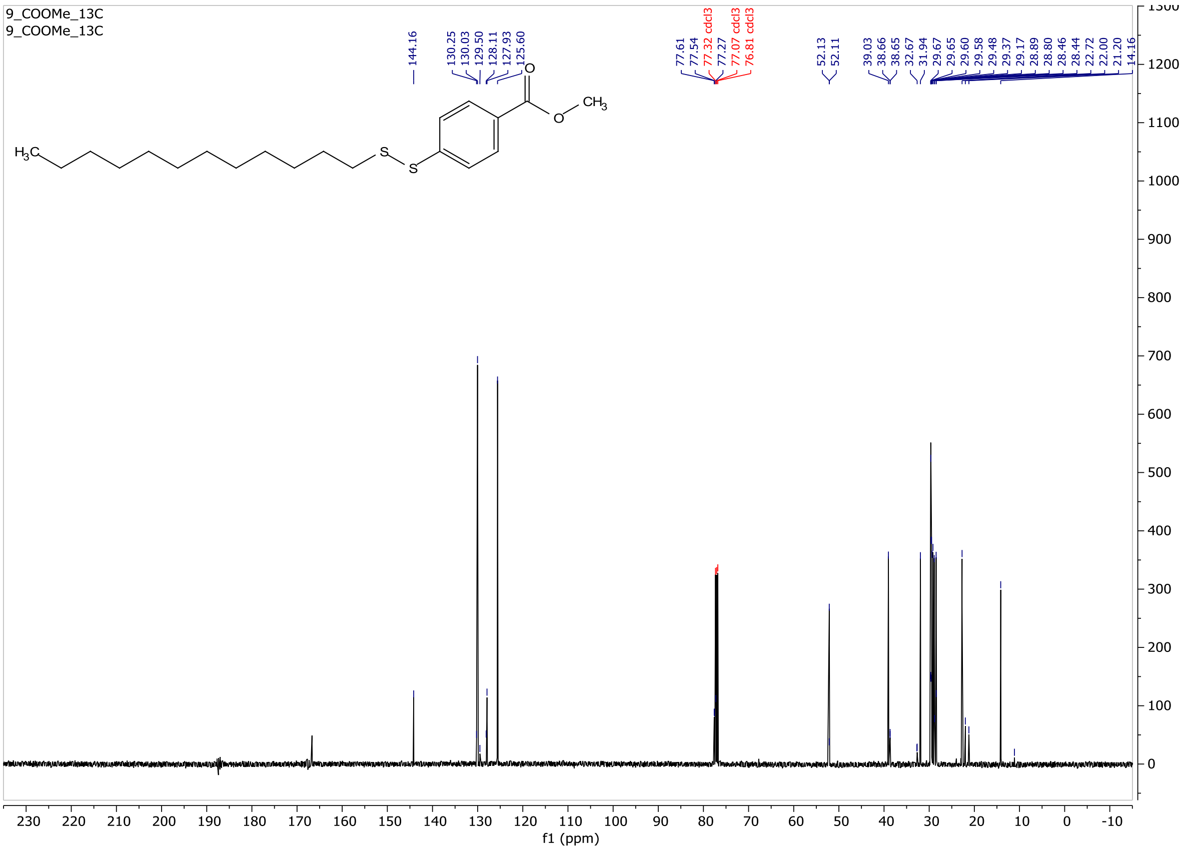

Figure S24. ^{13}C NMR spectrum of 3l

1-benzyl-2-dodecyldisulfane 3m

Chromatography: PE, R_f =0,44, yellowish oil, yield 92%,

^{1}H NMR ^{1}H NMR (500 MHz, cdcl$_3$) δ 7.36 – 7.32 (m, 3H), 7.31 – 7.24 (m, 1H), 3.89 (d, *J* = 1.8 Hz, 2H), 2.44 – 2.34 (m, 2H), 1.55 (t, *J* = 7.0 Hz, 2H), 1.26 (d, *J* = 7.5 Hz, 21H), 0.89 (td, *J* = 7.1, 2.1 Hz, 3H).

^{13}C NMR . ^{13}C NMR (126 MHz, cdcl$_3$) δ 153.20, 137.63, 129.30, 128.48, 127.36, 77.29, 77.03, 76.78, 43.73, 38.68, 31.93, 29.66, 29.60, 29.50, 29.37, 29.17, 29.03, 28.49, 22.71, 14.15.

HRMS (ESI): *m*/*z* [M + Na]$^+$ calcd for $C_{19}H_{32}NaS_2$: 347.1838; found: 347.1836.

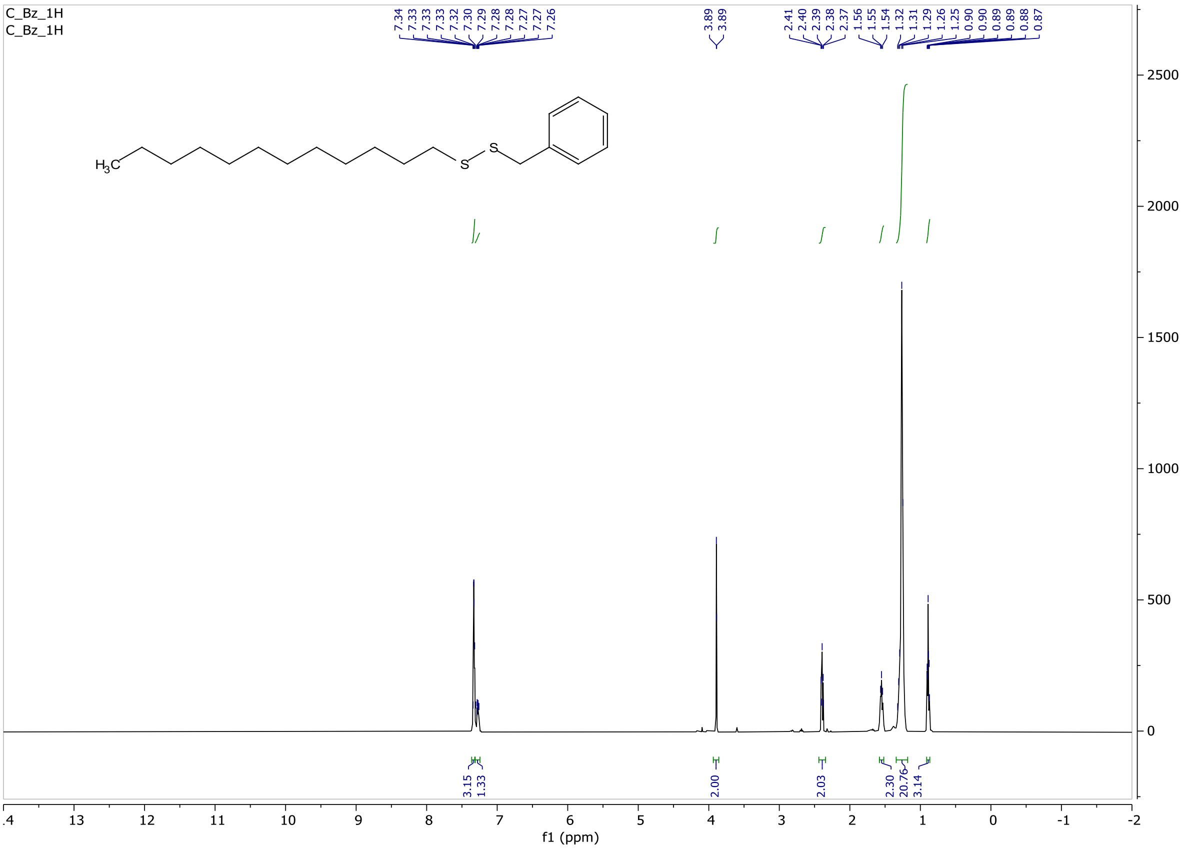

Figure S25. ^{1}H NMR spectrum of 3m

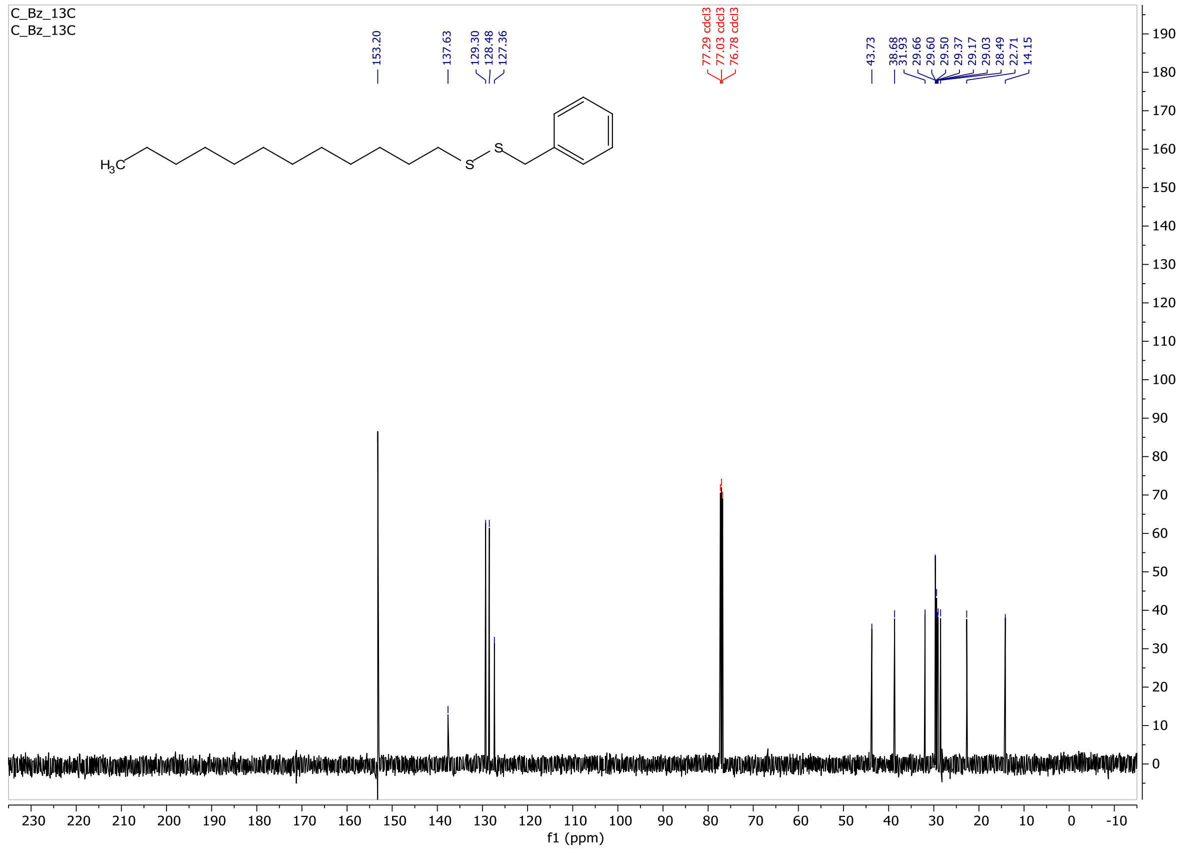

Figure S26. ^{13}C NMR spectrum of 3m

11-(benzyldisulfanyl)undecan-1-ol 3n

Chromatography: PE:DCM (4:1), R_f =0,35, yellowish oil, yield 92%,

^{1}H NMR ^{1}H NMR (500 MHz, cdcl$_3$) δ 7.37 – 7.22 (m, 5H), 3.88 (s, 2H), 3.63 (t, J = 6.5 Hz, 2H), 2.39 (t, J = 7.3 Hz, 2H), 1.55 (h, J = 7.4 Hz, 5H), 1.41 – 1.19 (m, 15H).

^{13}C NMR . ^{13}C NMR (126 MHz, cdcl$_3$) δ 137.61, 129.45, 129.29, 128.59, 128.48, 127.36, 77.30, 77.05, 76.79, 63.07, 43.71, 43.05, 38.66, 32.79, 29.57, 29.49, 29.45, 29.42, 29.15, 29.01, 28.47, 28.45, 25.74.

HRMS (ESI): m/z [M + Na]$^+$ calcd for $C_{18}H_{30}NaOS_2$: 349.1630; found: 349.1627.

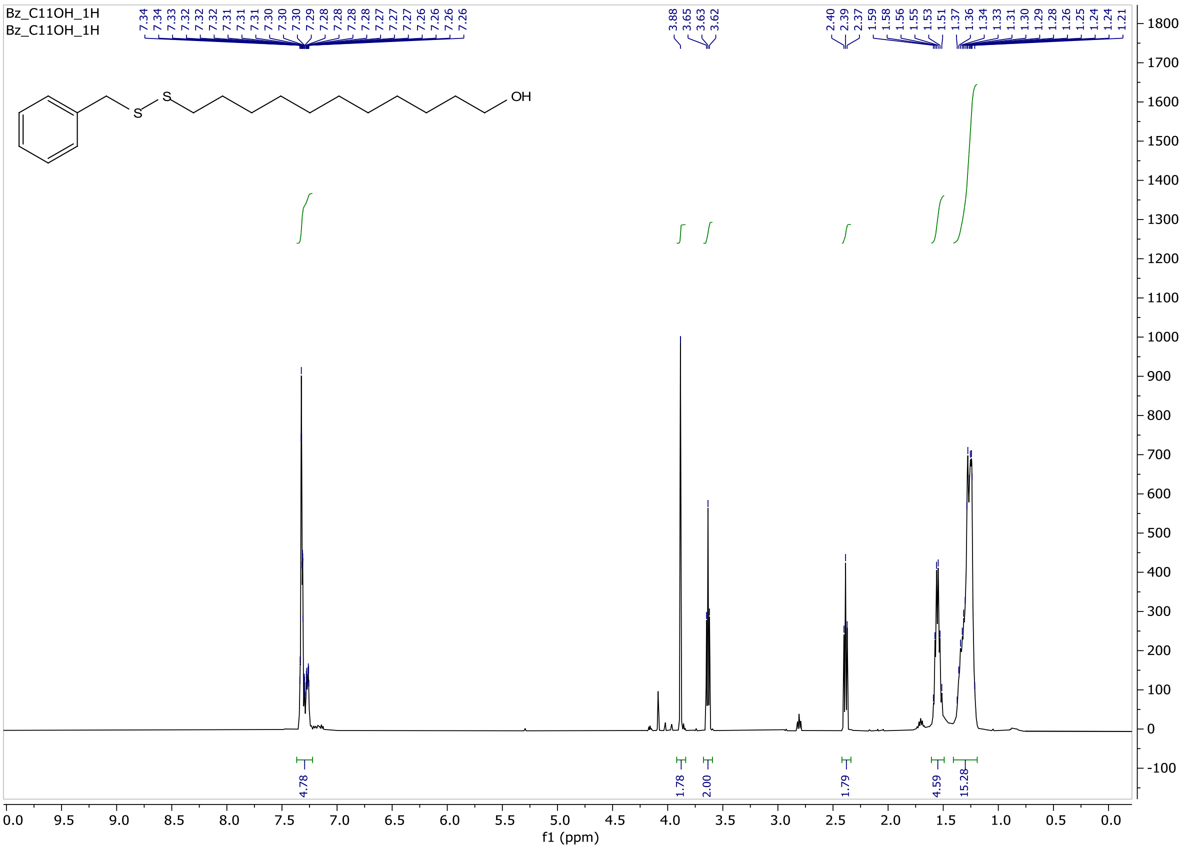

Figure S27. ^{1}H NMR spectrum of 3n

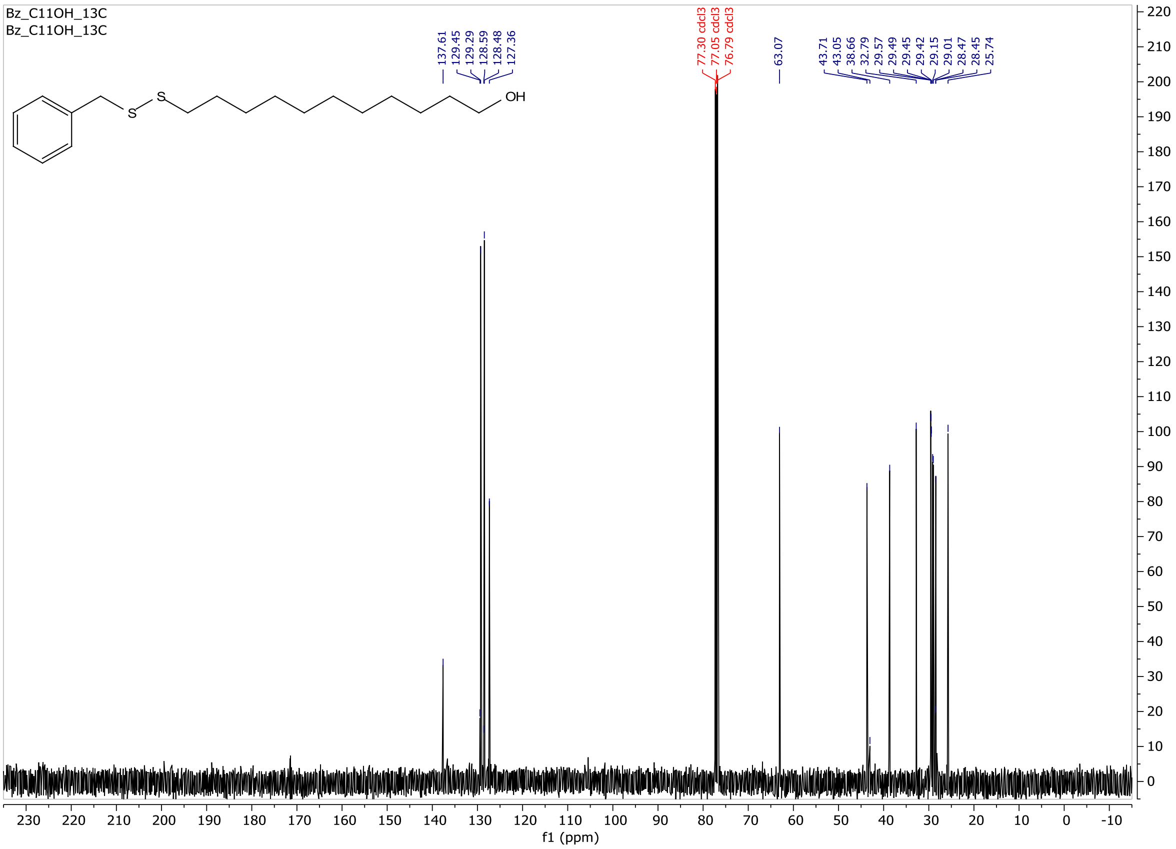

Figure S28. ^{13}C NMR spectrum of 3n

1-benzyl-2-(p-tolyl)disulfane 3o

Chromatography: PE, R_f =0,41, yellowish oil, yield 92%,

^{1}H NMR ^{1}H NMR (500 MHz, cdcl$_3$) δ 7.40 – 7.37 (m, 2H), 7.35 – 7.26 (m, 5H), 7.15 – 7.12 (m, 2H), 3.97 (s, 2H), 2.37 (s, 3H).

^{13}C NMR . ^{13}C NMR (126 MHz, cdcl$_3$) δ 137.20, 136.71, 133.61, 131.43, 129.98, 129.76, 129.47, 129.44, 128.69, 128.61, 128.56, 127.61, 127.52, 77.32, 77.07, 76.81, 43.30, 21.09.

HRMS (ESI): *m*/*z* [M + Na]$^+$ calcd for $C_{14}H_{14}NaS_2$: 269.0429; found: 269.0431.

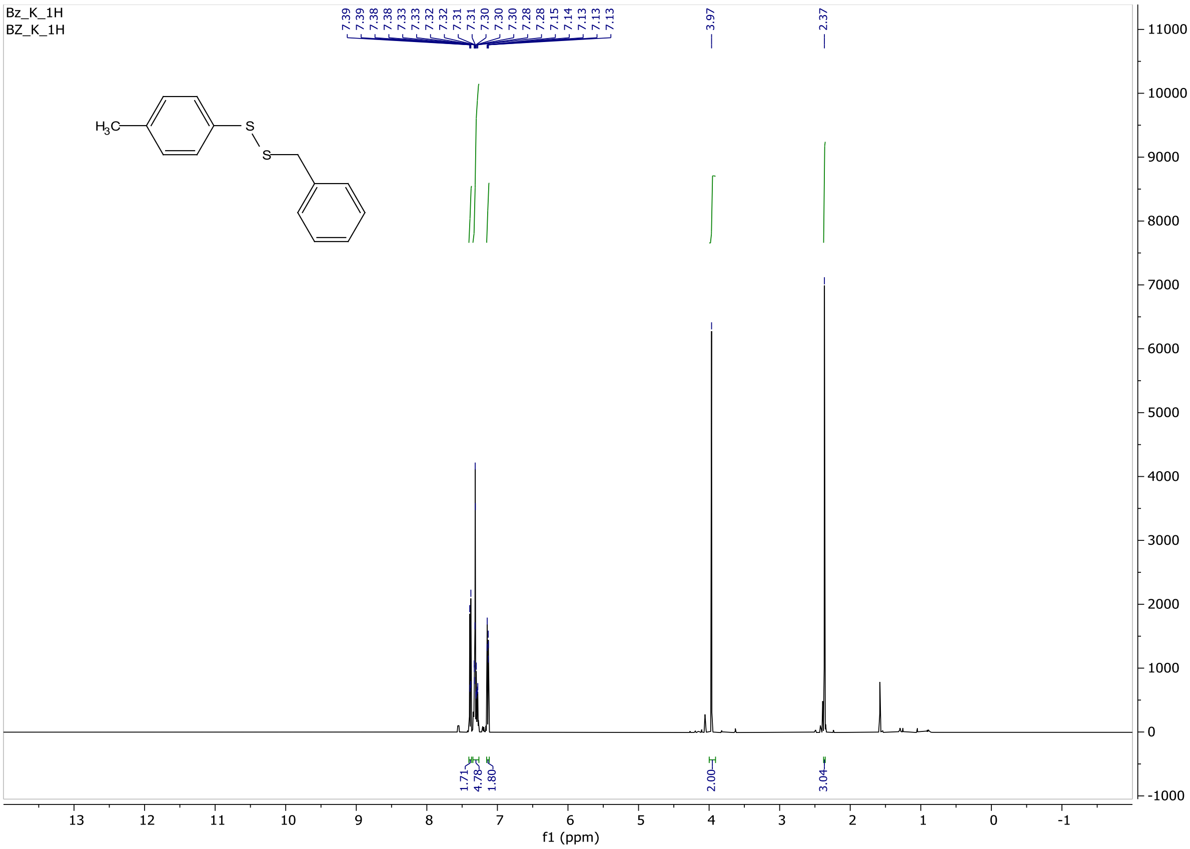

Figure S29. ^{1}H NMR spectrum of 3o

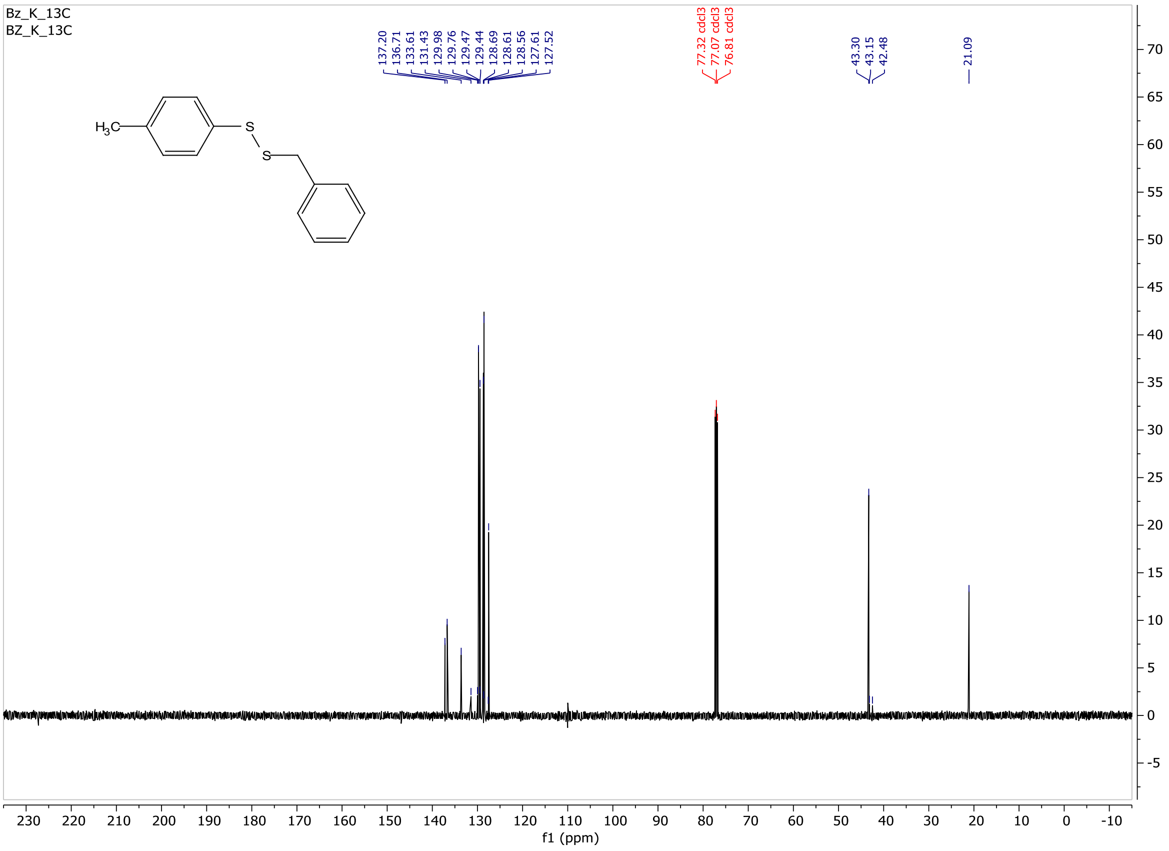

Figure S30. ^{13}C NMR spectrum of 3o

1-benzyl-2-trityldisulfane 3p

Chromatography: PE, R_f =0,44, yellowish oil, yield 92%,

^{1}H NMR ^{1}H NMR (500 MHz, cdcl$_3$) δ 7.54 – 7.49 (m, 5H), 7.34 (t, *J* = 7.6 Hz, 5H), 7.32 – 7.26 (m, 5H), 7.25 – 7.18 (m, 3H), 6.99 (d, *J* = 7.2 Hz, 2H), 2.89 (s, 2H).

^{13}C NMR . ^{13}C NMR (126 MHz, cdcl$_3$) δ 143.88, 136.69, 130.45, 130.35, 130.25, 129.48, 129.39, 129.24, 128.54, 128.40, 128.02, 127.97, 127.93, 127.31, 127.30, 127.20, 126.98, 77.32, 77.06, 76.81, 41.66.

HRMS (ESI): *m*/*z* $[M + Na]^+$ calcd for $C_{26}H_{22}NaS_2$: 421.1055; found: 421.1051.

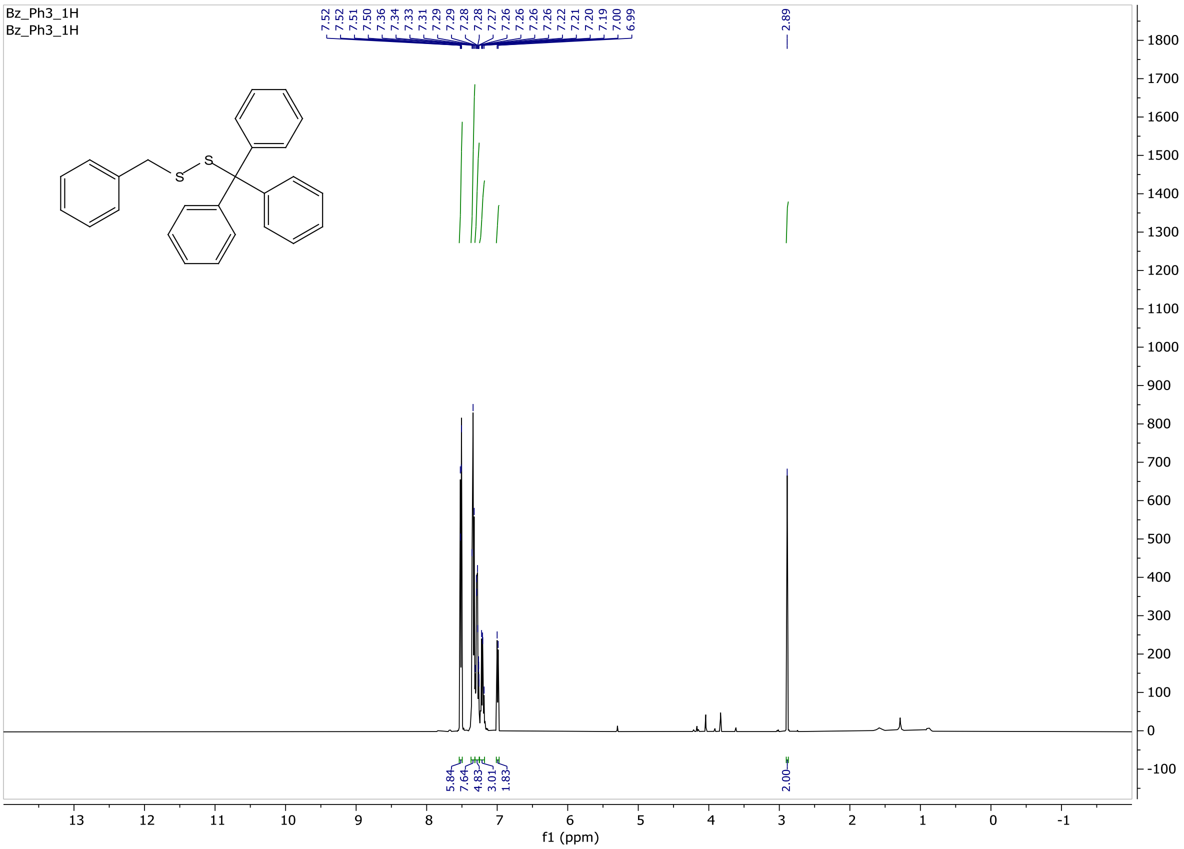

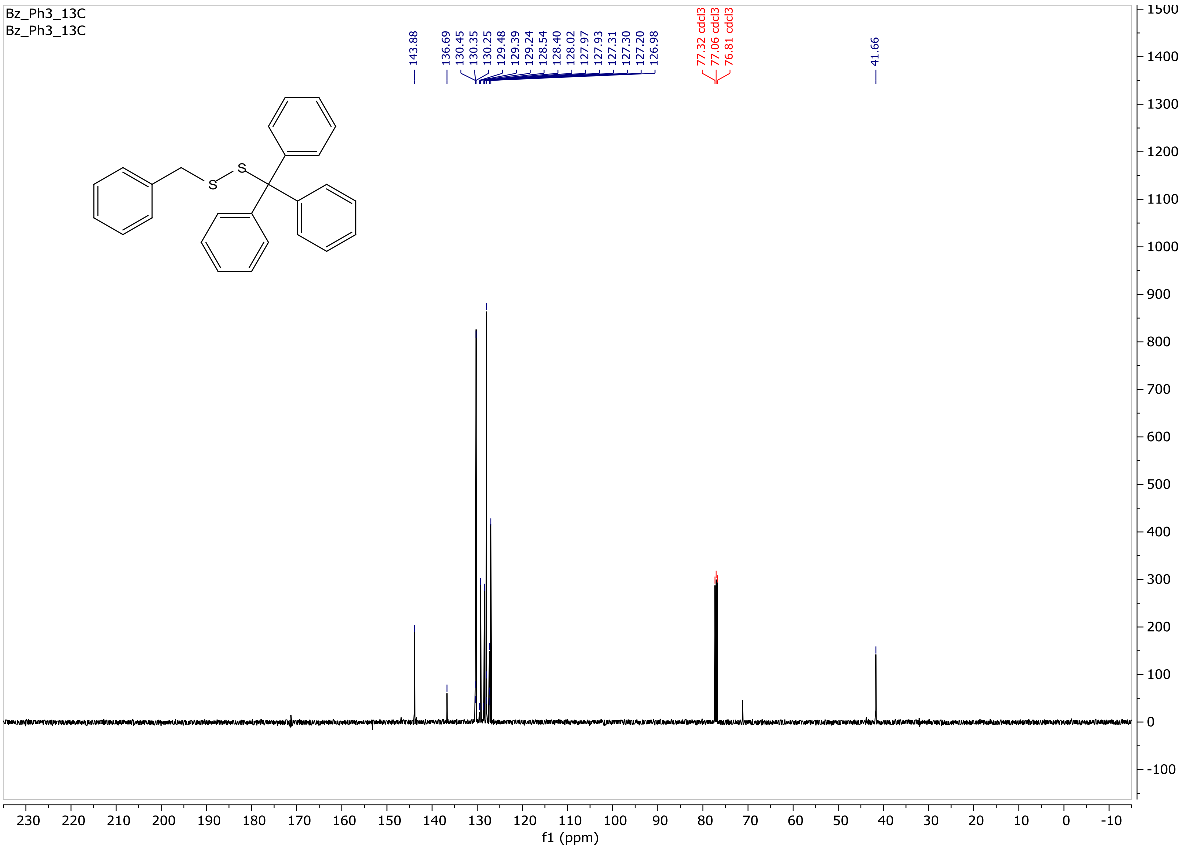
Bz_Ph3_13C
Bz_Ph3_13C
143.88
136.69
130.45
130.35
130.25
129.48
129.39
129.24
128.54
128.40
128.02
127.97
127.93
127.31
127.30
127.20
126.98
77.32 cdcl3
77.06 cdcl3
76.81 cdcl3
41.66
S
S
f1 (ppm)

2-(benzyldisulfanyl)benzo[d]thiazole 3q

Chromatography: PE:DCM (2:1), R_f =0,33, yellowish oil, yield 92%,

^{1}H NMR ^{1}H NMR (500 MHz, cdcl$_3$) δ 7.90 (dt, *J* = 8.1, 0.9 Hz, 1H), 7.82 (dt, *J* = 8.1, 1.0 Hz, 1H), 7.49 – 7.43 (m, 1H), 7.39 – 7.31 (m, 5H), 7.31 – 7.27 (m, 1H), 4.20 (s, 2H).

^{13}C NMR . ^{13}C NMR (126 MHz, cdcl$_3$) δ 155.08, 135.90, 135.43, 129.55, 128.80, 128.10, 126.26, 124.62, 122.18, 121.14, 77.32, 77.07, 76.81, 43.97.

HRMS (ESI): *m*/*z* [M + H]$^+$ calcd for $C_{14}H_{12}NS_3$: 290.0126; found: 290.0127.

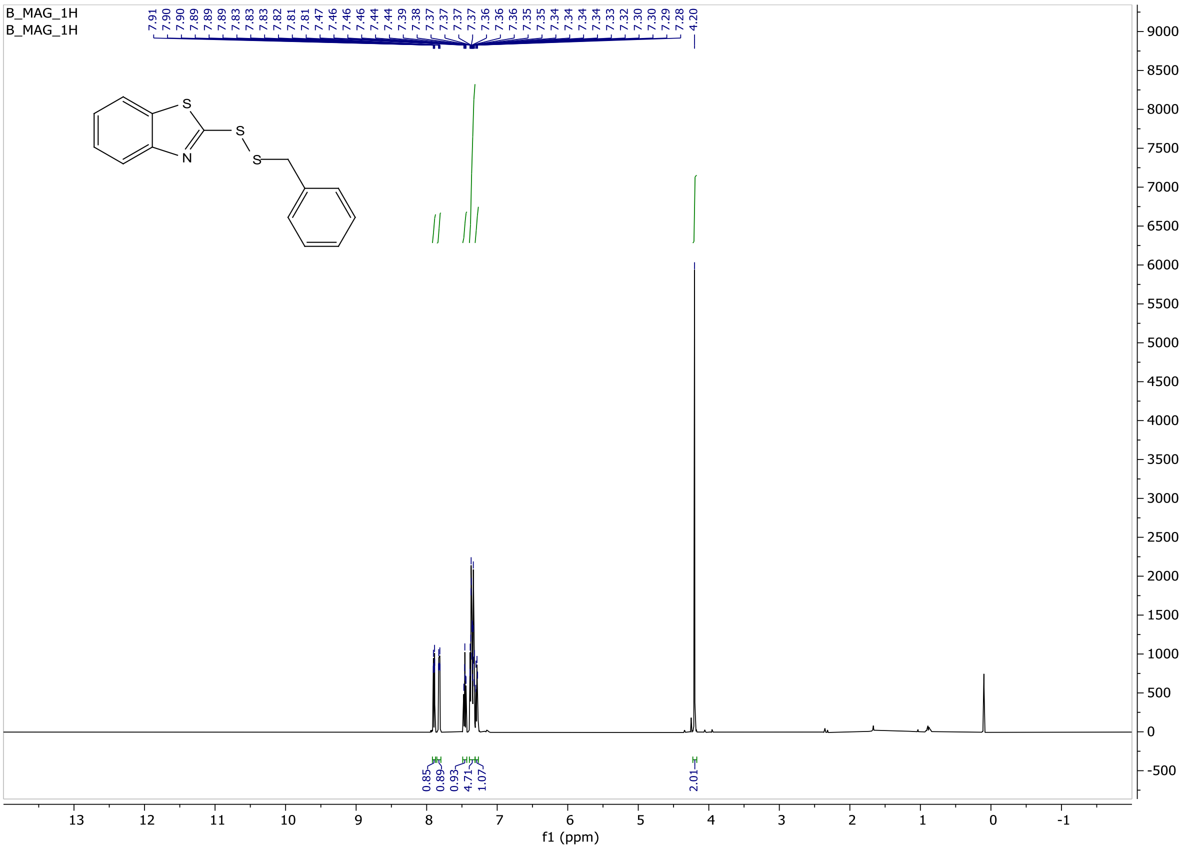

Figure S33. ^{1}H NMR spectrum of 3q

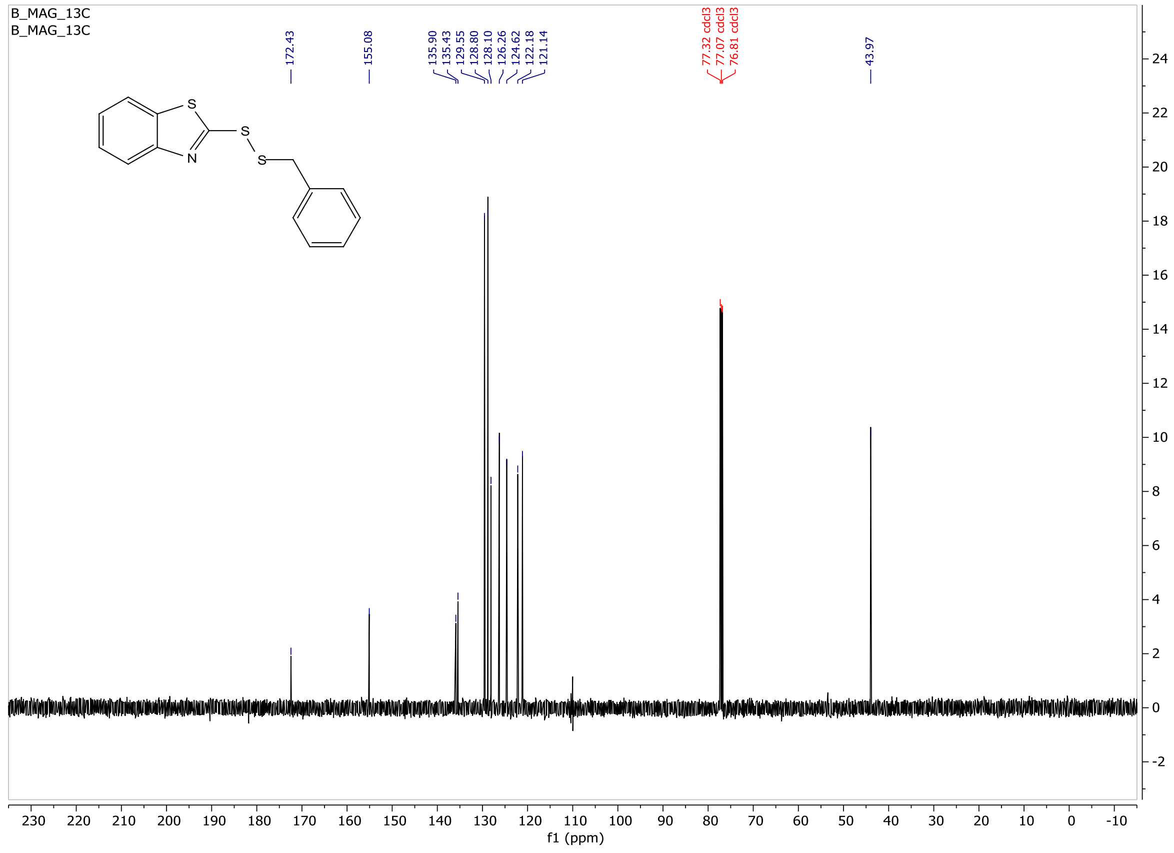

Figure S34. ^{13}C NMR spectrum of 3q

1-benzyl-2-(4-methoxybenzyl)disulfane 3r

Chromatography: PE, R_f =0,32, yellowish oil, yield 92%,

^{1}H NMR ^{1}H NMR (500 MHz, cdcl$_3$) δ 7.36 – 7.31 (m, 2H), 7.31 – 7.25 (m, 3H), 7.18 – 7.14 (m, 2H), 6.88 – 6.84 (m, 2H), 3.80 (s, 3H), 3.64 (s, 2H), 3.56 (s, 2H).

^{13}C NMR .. ^{13}C NMR (126 MHz, cdcl$_3$) δ 159.02, 153.20, 137.49, 130.61, 130.53, 129.44, 129.28, 128.62, 128.50, 127.43, 114.03, 113.89, 77.30, 77.05, 76.80, 55.29, 43.31, 42.70.

HRMS (ESI): *m*/*z* $[M + Na]^+$ calcd for $C_{15}H_{16}NaOS_2$: 299.0535; found: 299.0533.

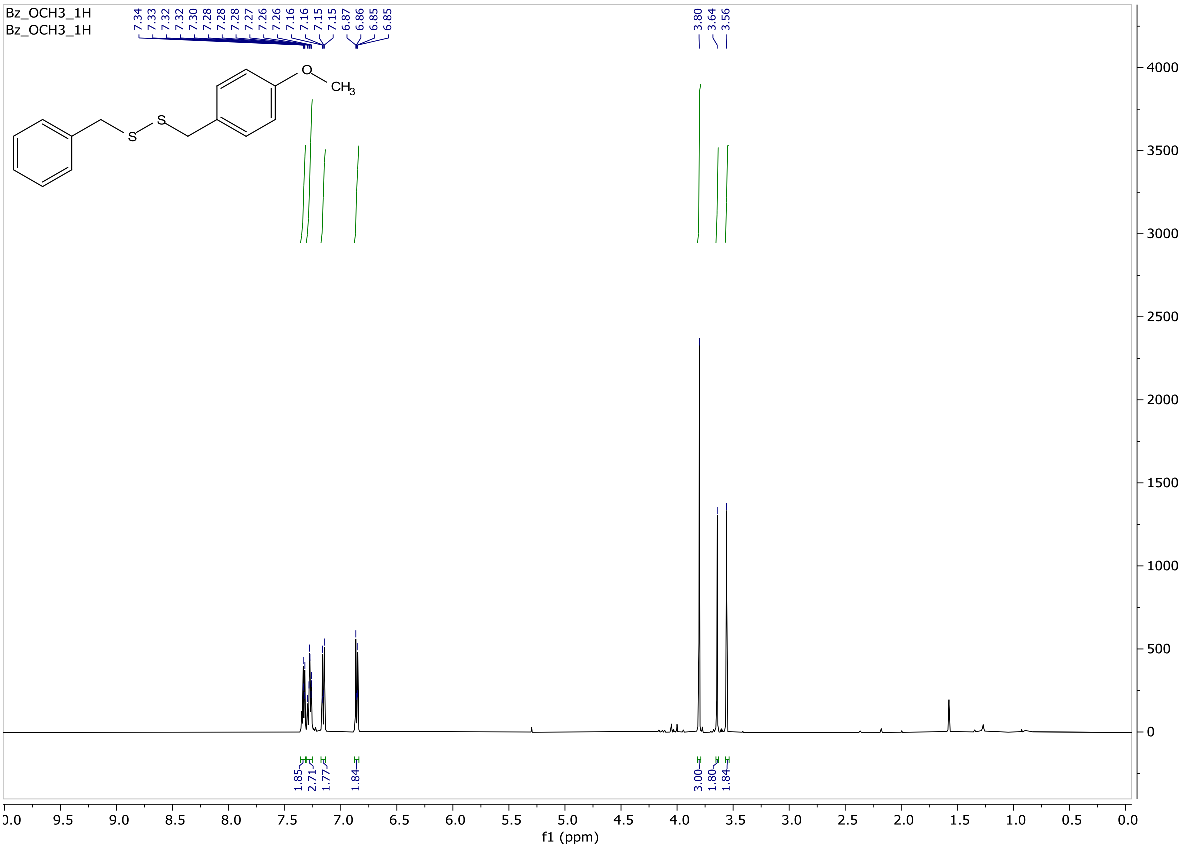

Figure S35. ^{1}H NMR spectrum of 3r

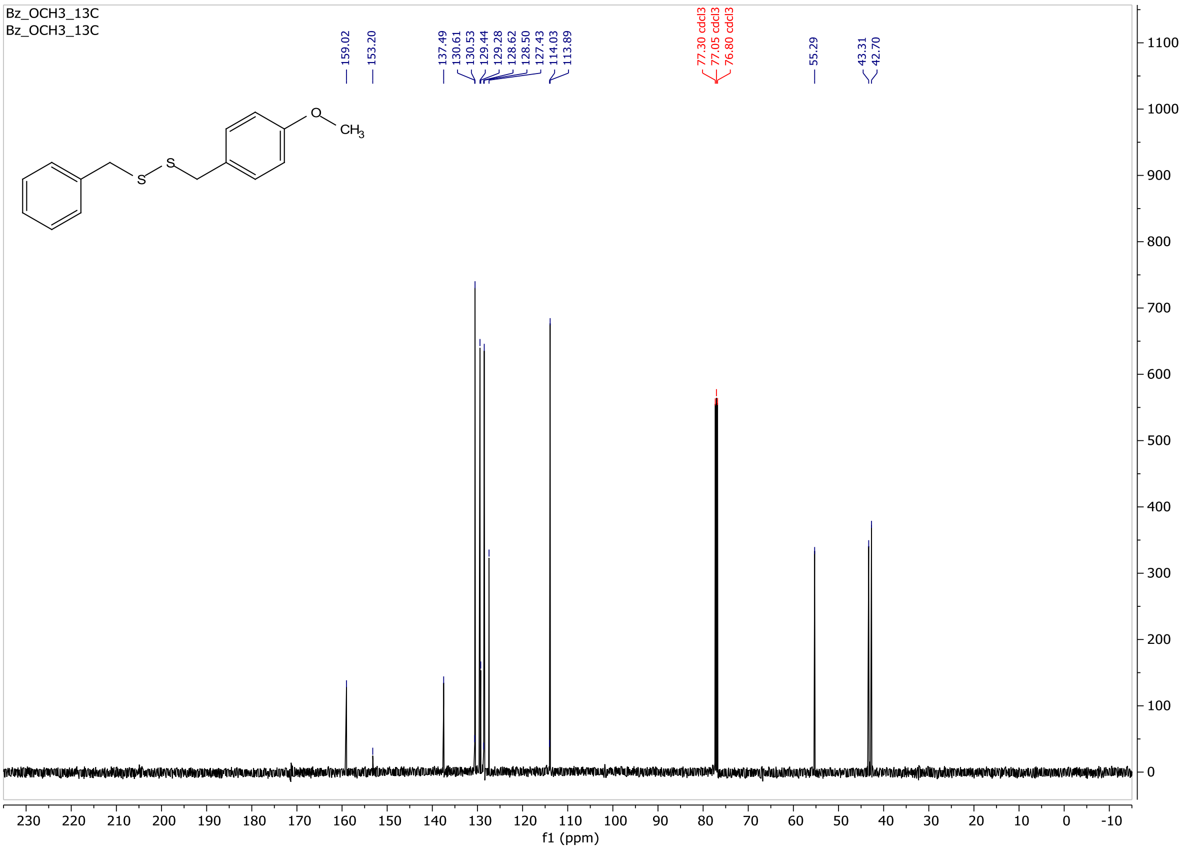

Figure S36. ^{13}C NMR spectrum of 3r

1-benzyl-2-(4-nitrobenzyl)disulfane 3s

Chromatography: PE, R_f =0,34, yellowish oil, yield 92%,

^{1}H NMR ^{1}H NMR (500 MHz,) δ 8.20 – 8.08 (m, 2H), 7.42 – 7.23 (m, 8H), 3.72 (s, 2H), 3.51 (s, 2H).

^{13}C NMR ^{13}C NMR (126 MHz, cdcl$_3$) δ 153.20, 145.04, 137.20, 130.15, 130.09, 129.42, 129.40, 128.66, 128.49, 127.70, 123.83, 123.65, 77.30, 77.04, 76.79, 43.41, 41.92.

HRMS (ESI): *m*/*z* [M + Na]$^+$ calcd for $C_{14}H_{13}NNaO_2S_2$: 314.0280; found: 314.0278.

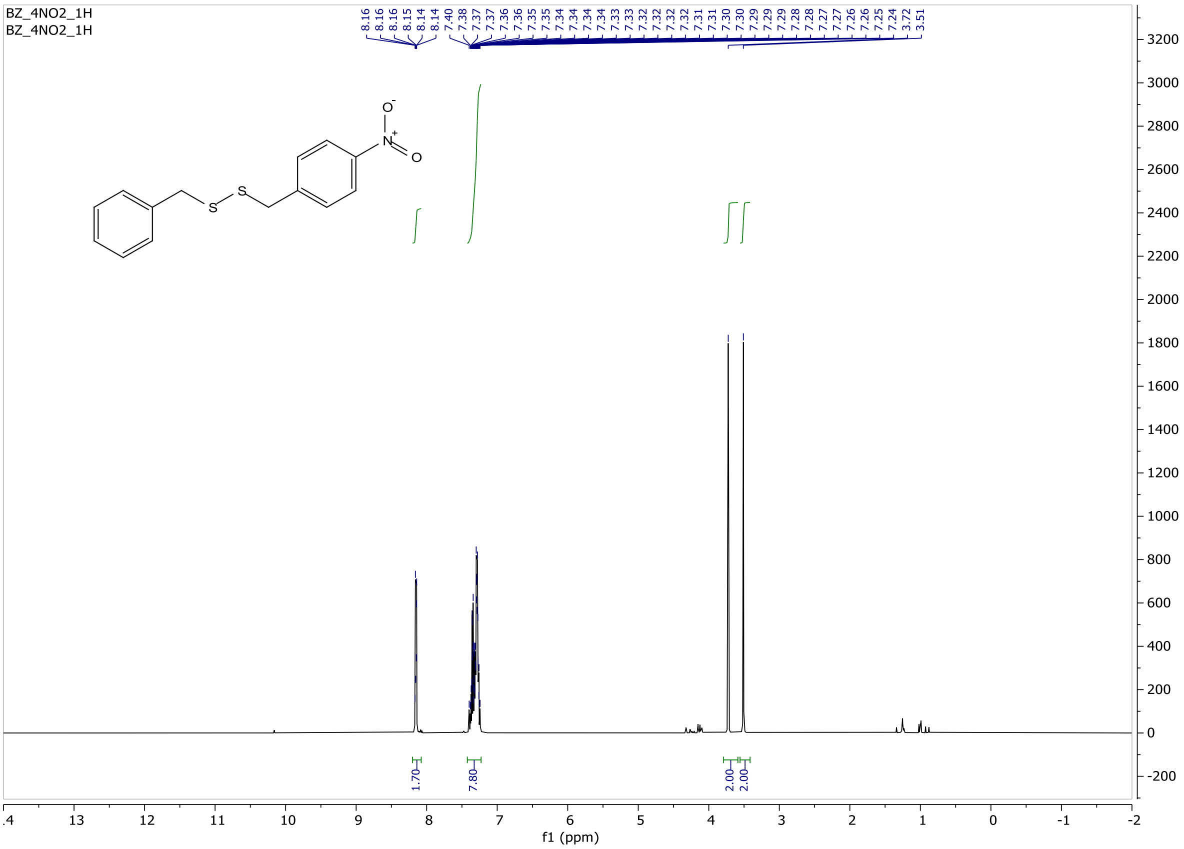

Figure S37. ^{1}H NMR spectrum of 3s

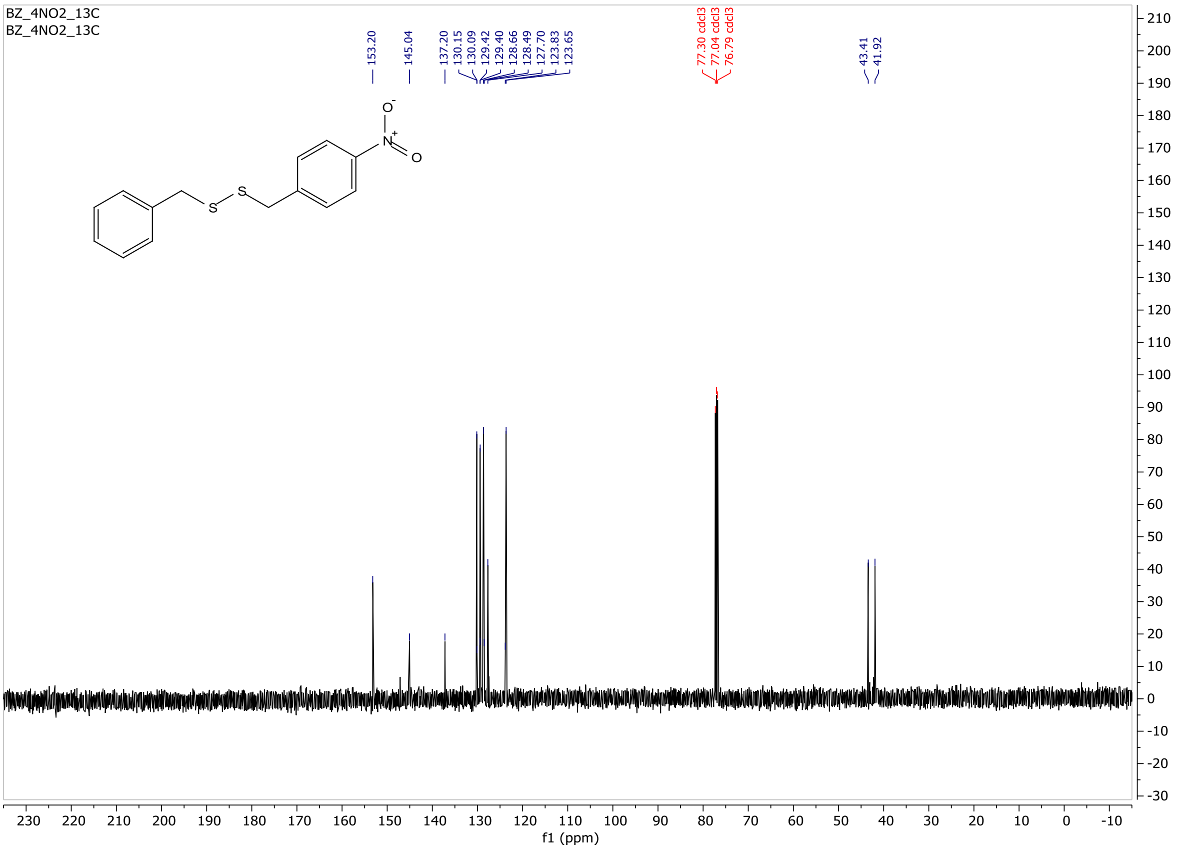

Figure S38. ^{13}**C NMR spectrum of 3s**

1-benzyl-2-(3-nitrobenzyl)disulfane 3t

Chromatography: PE, R_f =0,37, yellowish oil, yield 92%,

^{1}H NMR ^{1}H NMR (500 MHz, cdcl$_3$) δ 8.11 (d, *J* = 6.7 Hz, 1H), 7.97 (q, *J* = 1.4 Hz, 1H), 7.50 – 7.43 (m, 2H), 7.40 – 7.36 (m, 1H), 7.35 – 7.28 (m, 3H), 3.73 (s, 2H), 3.49 (s, 2H).

^{13}C NMR ^{13}C NMR (126 MHz, cdcl$_3$) δ 153.20, 135.39, 129.44, 129.34, 128.70, 127.73, 124.33, 122.37, 77.30, 77.04, 76.79, 43.36, 41.70.

HRMS (ESI): *m*/*z* [M + Na]$^+$ calcd for $C_{14}H_{13}NNaO_2S_2$: 314.0280; found: 314.0281.

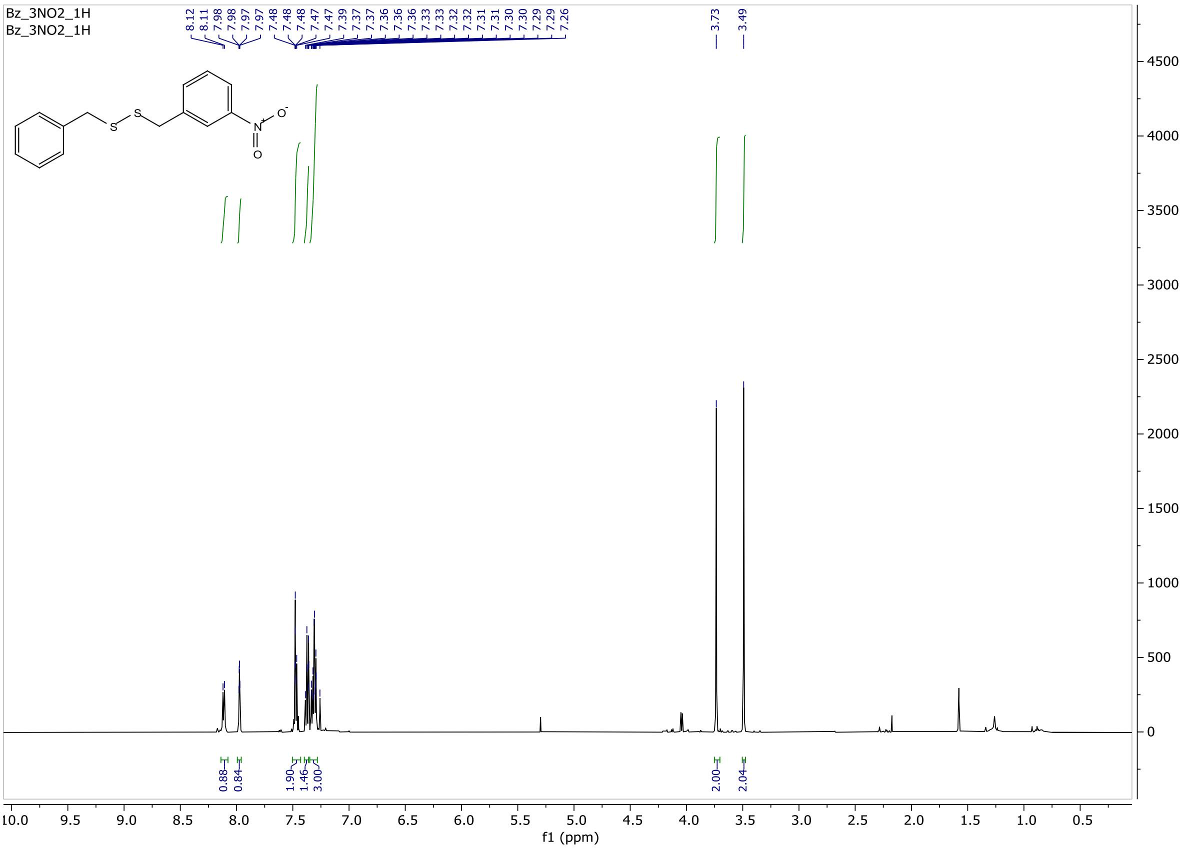

Figure S39. ^{1}H NMR spectrum of 3t

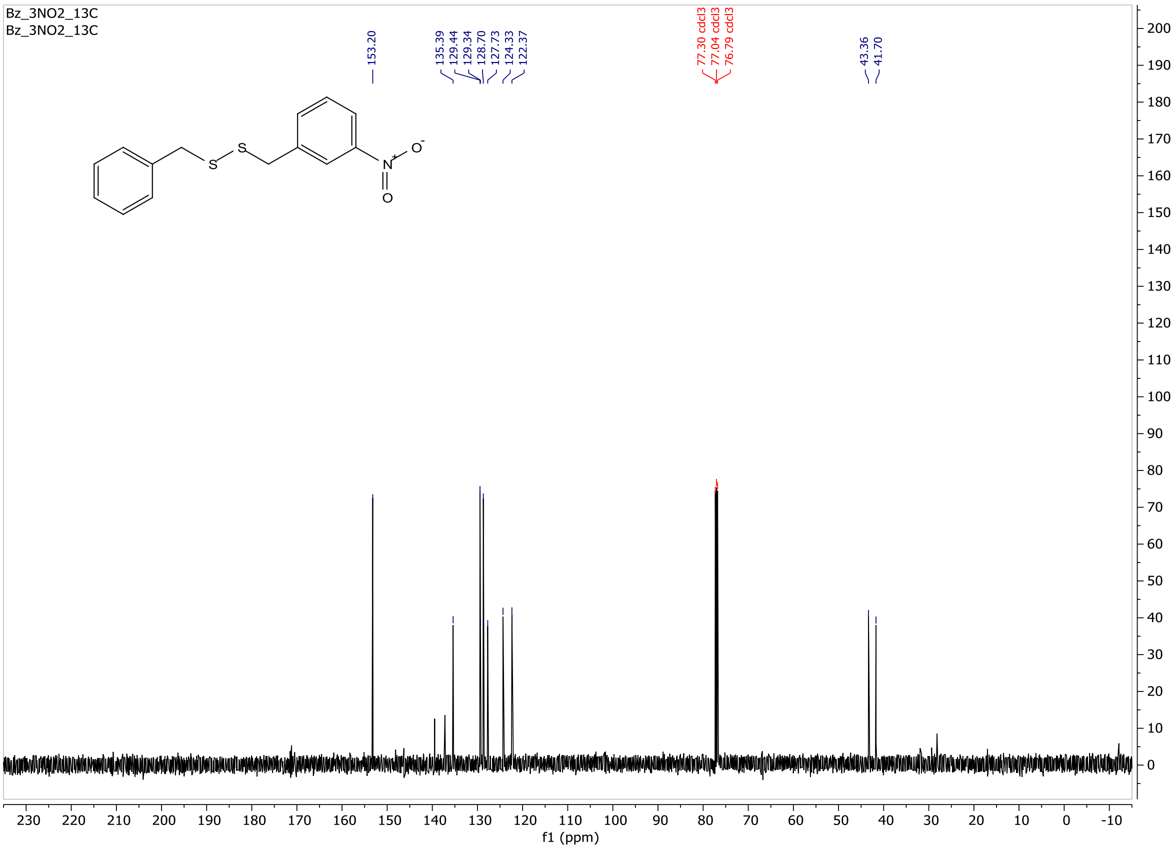

Figure S40. ^{13}C NMR spectrum of 3t

1-benzyl-2-(2-nitrobenzyl)disulfane 3u

Chromatography: PE, R_f =0,38, yellowish oil, yield 92%,

^{1}H NMR ^{1}H NMR (500 MHz, $cdcl_3$) δ 8.04 (dd, *J* = 8.2, 1.4 Hz, 1H), 7.56 (td, *J* = 7.5, 1.4 Hz, 1H), 7.44 (td, *J* = 7.8, 1.5 Hz, 1H), 7.33 (t, *J* = 7.2 Hz, 2H), 7.31 – 7.23 (m, 4H), 3.96 (s, 2H), 3.66 (s, 2H).

^{13}C NMR ^{13}C NMR (126 MHz, $cdcl_3$) δ 137.05, 133.49, 133.13, 132.86, 129.35, 128.65, 128.55, 127.62, 125.48, 77.32, 77.07, 76.82, 43.60, 40.46.

HRMS (ESI): *m/z* $[M + Na]^+$ calcd for $C_{14}H_{13}NNaO_2S_2$: 314.0280; found: 314.0281.

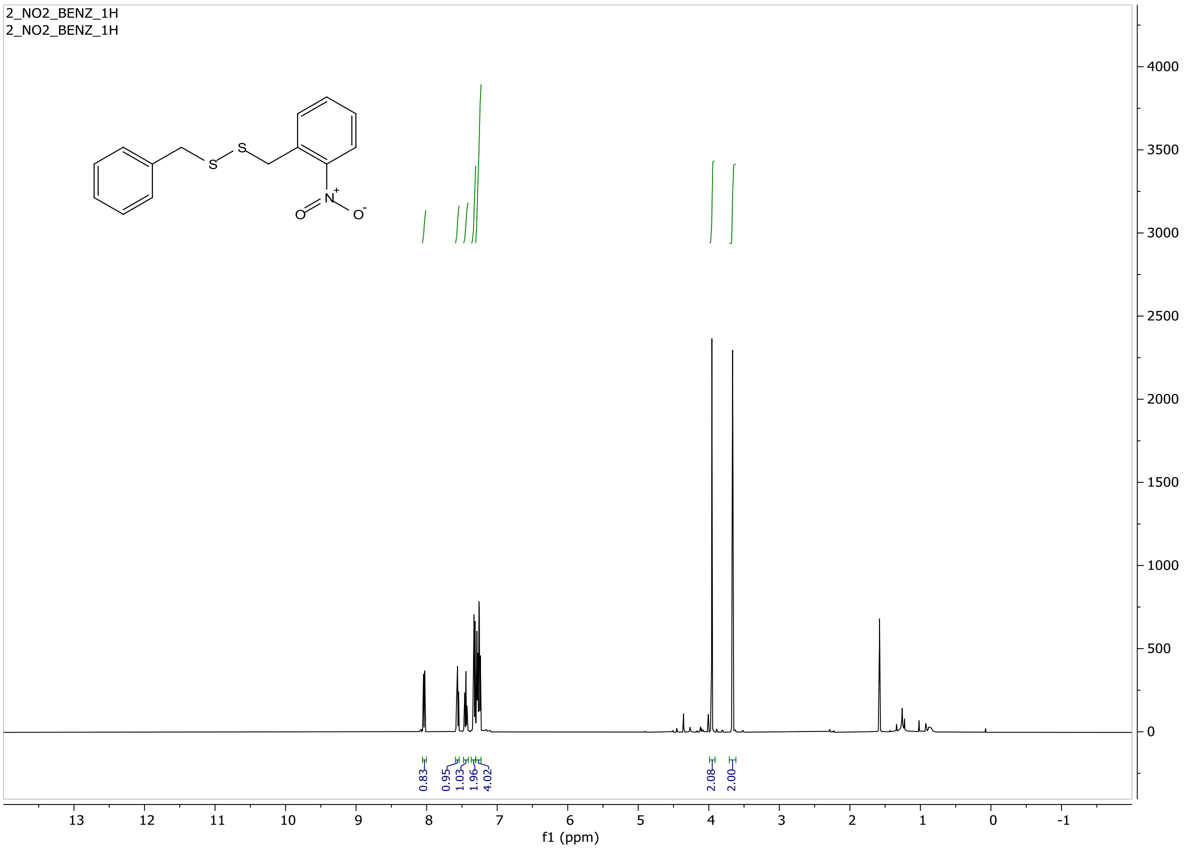

Figure S41. ^{1}H NMR spectrum of 3u

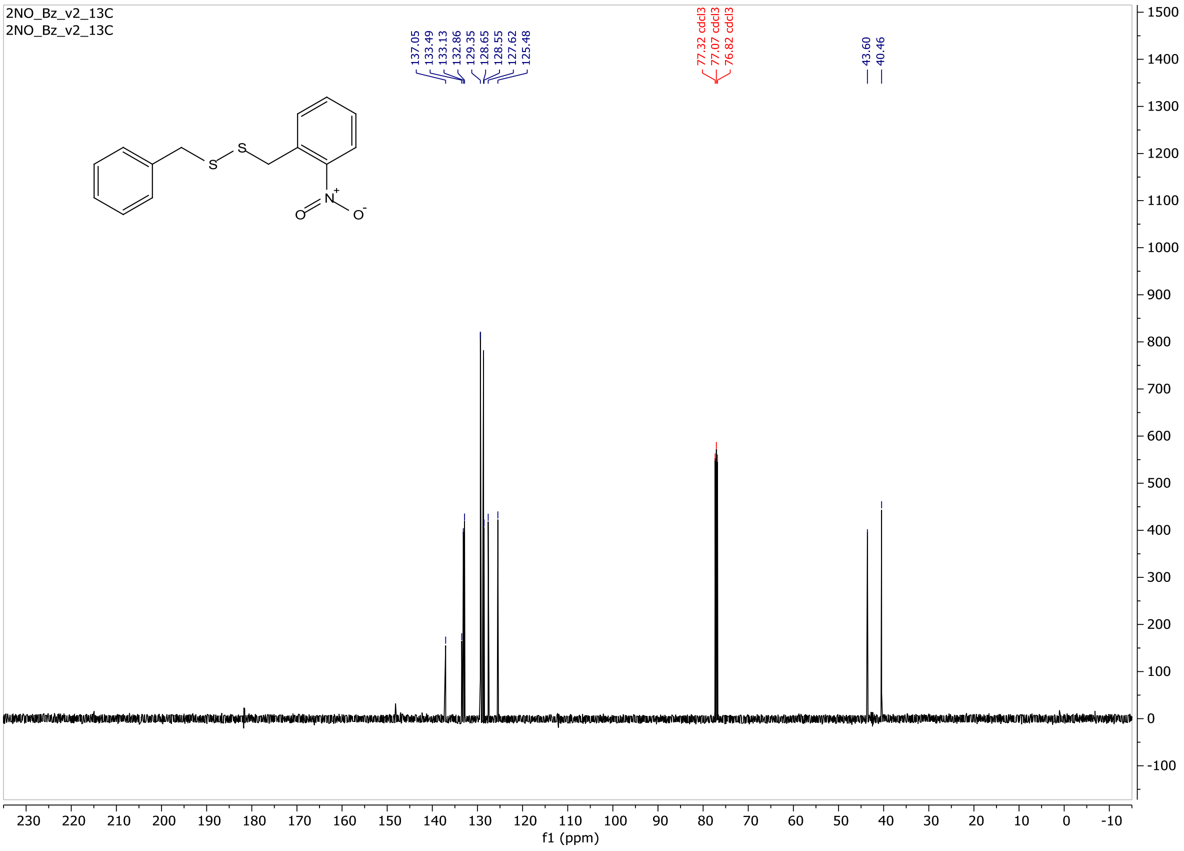

Figure S42. ^{13}C NMR spectrum of 3u

ethyl 2-amino-3-(benzyldisulfanyl)propanoate 3v

Chromatography CH_2Cl_2, R_f =0,41, yellowish oil, yield 97%,

^{1}H NMR ^{1}H NMR (500 MHz, cdcl$_3$) δ 7.35 – 7.31 (m, 3H), 7.31 – 7.25 (m, 1H), 4.16 (q, J = 7.1 Hz, 2H), 3.91 (s, 2H), 3.62 (dd, J = 8.0, 4.5 Hz, 1H), 2.74 (dd, J = 13.5, 4.5 Hz, 1H), 2.56 (dd, J = 13.5, 7.9 Hz, 1H), 1.67 (s, 2H), 1.27 (t, J = 7.2 Hz, 3H).

^{13}C NMR ^{13}C NMR (126 MHz, cdcl$_3$) δ 173.75, 137.14, 129.34, 128.60, 127.55, 77.31, 77.06, 76.80, 61.26, 53.53, 43.41, 43.39, 14.19.

HRMS (ESI): m/z [M + H]$^+$ calcd for $C_{12}H_{18}NO_2S_2$: 272.0779; found: 272.0781.

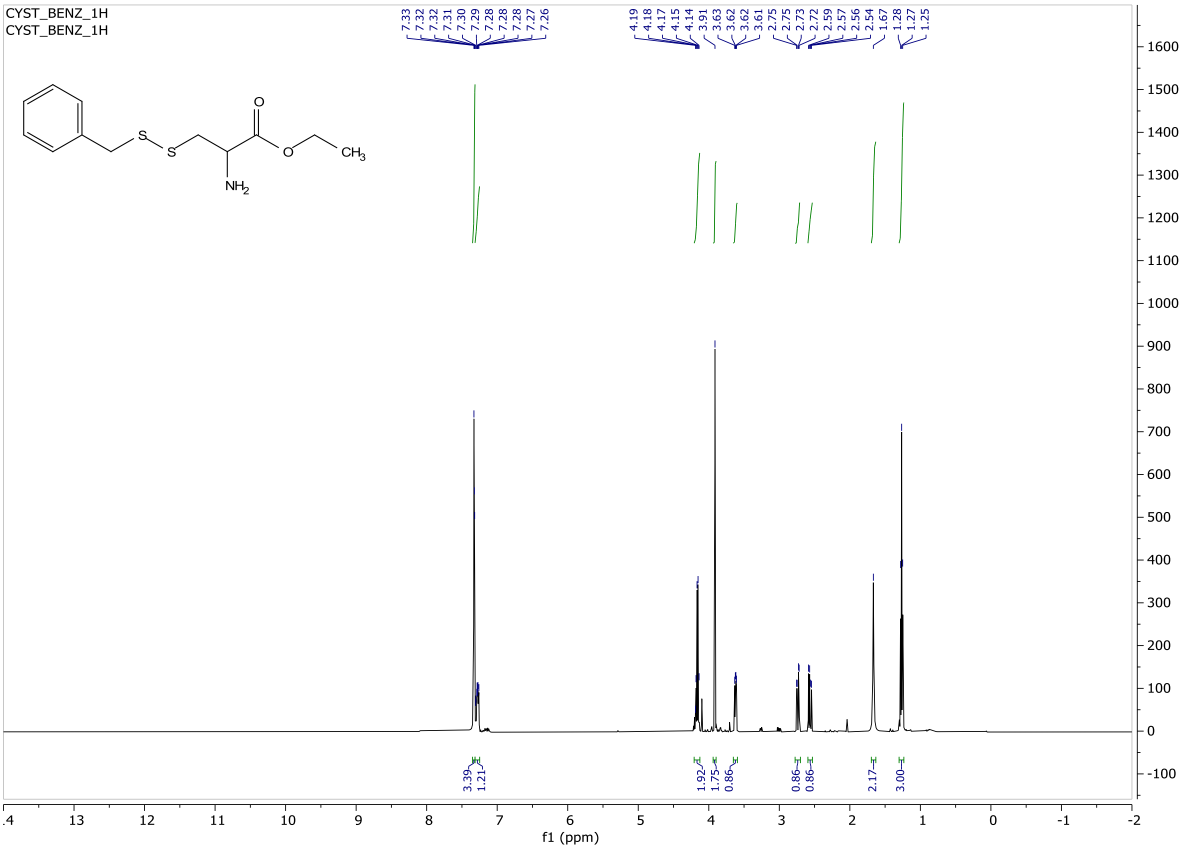

Figure S43. ^{1}H NMR spectrum of 3v

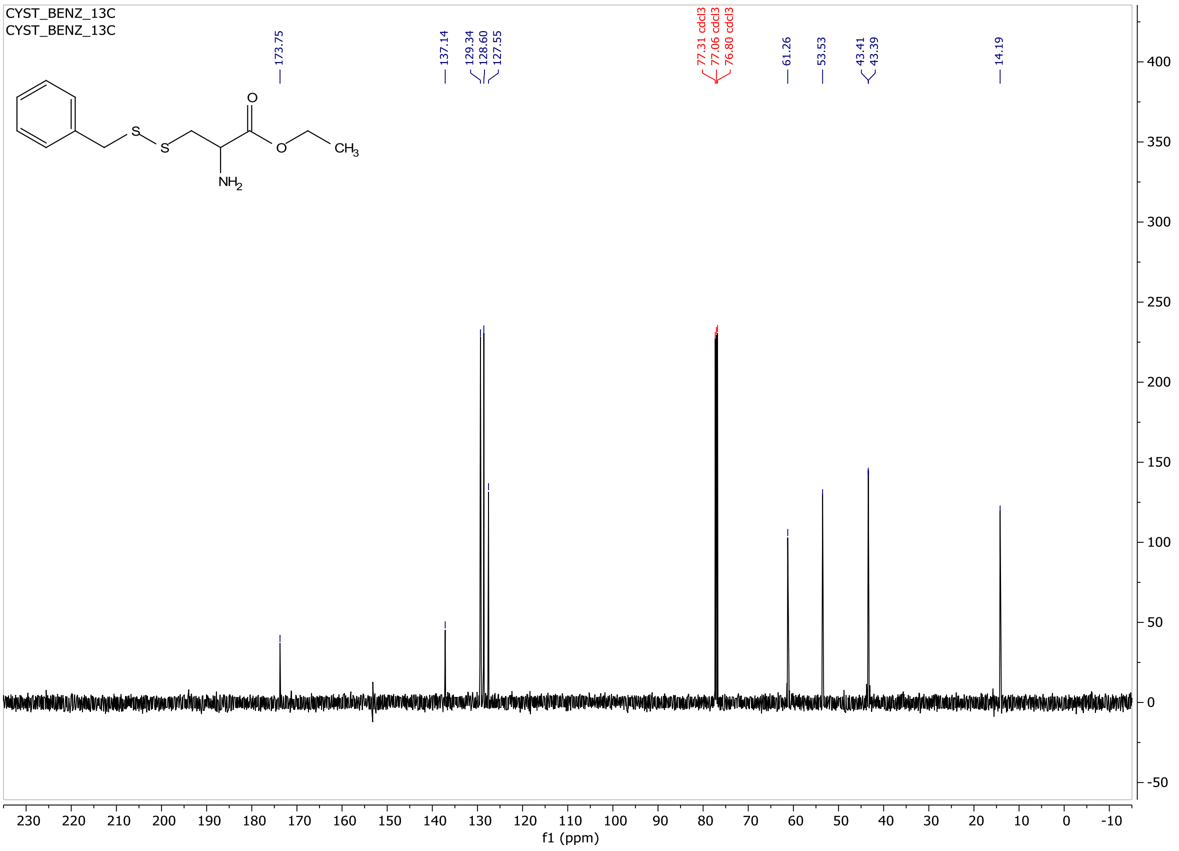

Figure S44. ^{13}C NMR spectrum of 3v

ethyl 2-amino-3-(benzyldisulfanyl)propanoate 3w

Chromatography PE, R_f =0,33, yellowish oil, yield 96%,

^{1}H NMR ^{1}H NMR (500 MHz, cdcl$_3$) δ 7.60 (d, J = 8.3 Hz, 2H), 7.39 – 7.31 (m, 4H), 7.28 (td, J = 8.3, 1.7 Hz, 3H), 3.72 (s, 2H), 3.50 (s, 2H).

^{13}C NMR ^{13}C NMR (126 MHz, cdcl$_3$) δ 142.97, 137.20, 132.21, 130.08, 129.41, 128.65, 127.69, 118.76, 111.14, 77.31, 77.26, 77.06, 76.80, 43.39, 42.35.

HRMS (ESI): m/z [M + H]$^+$ calcd for $C_{15}H_{14}NS_2$: 272.0562; found: 272.0565.

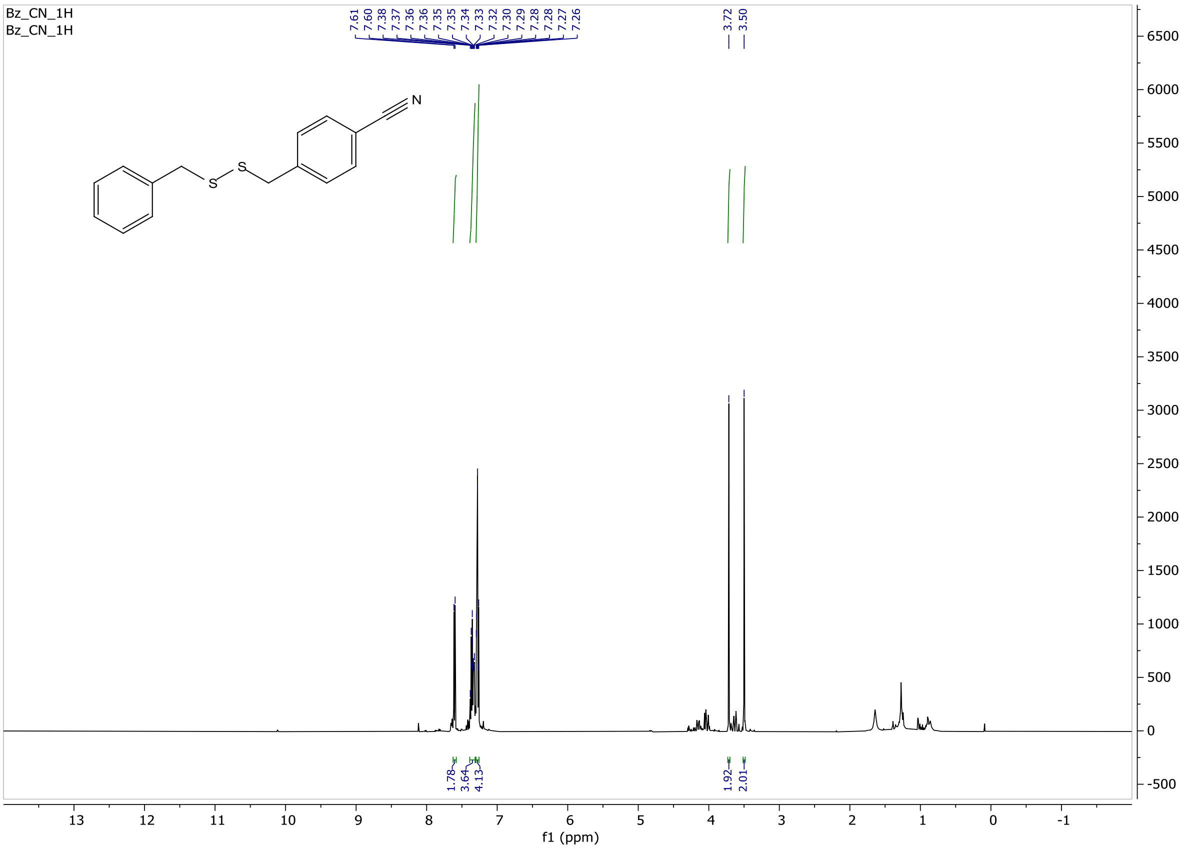

Figure S45. ^{1}H NMR spectrum of 3w

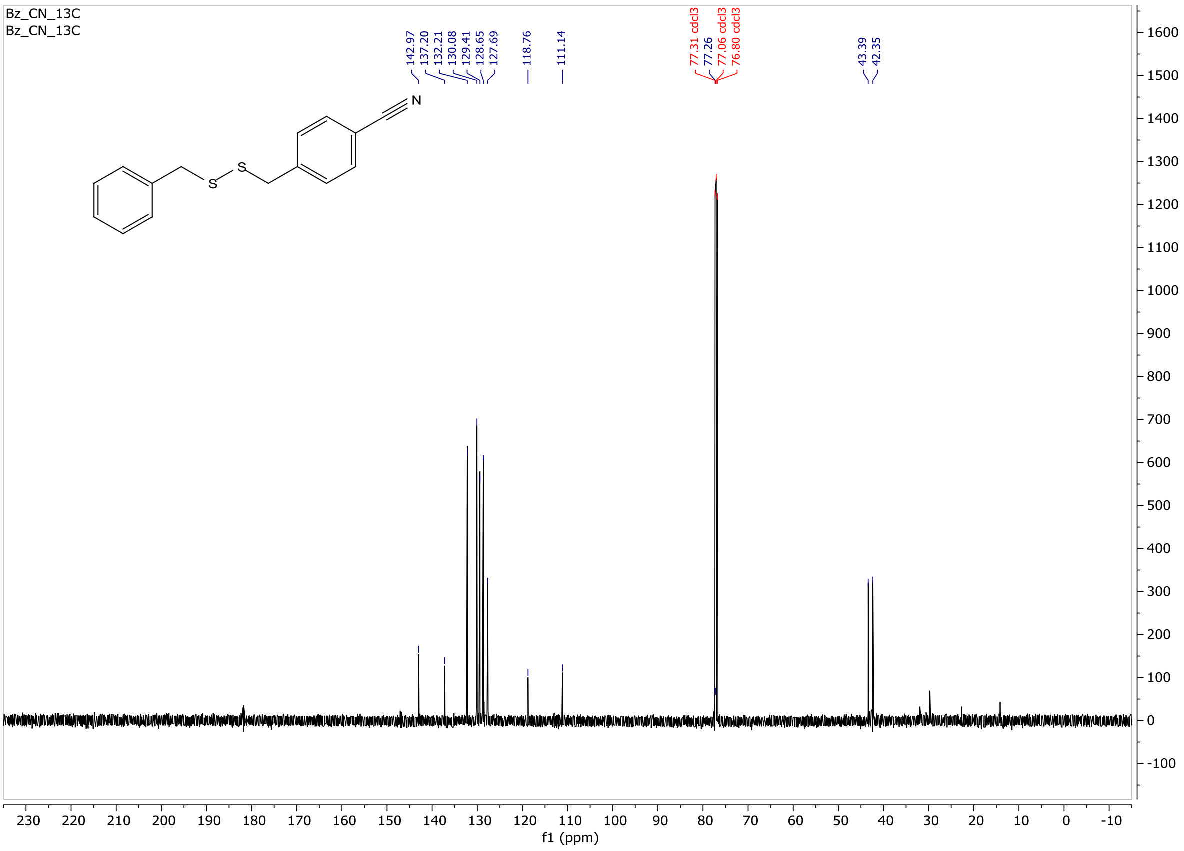

Figure S46. ^{13}**C NMR spectrum of 3w**

1-(4-methoxybenzyl)-2-(p-tolyl)disulfane 3x

Chromatography PE:CH_2Cl_2 (4:1), R_f =0,31, yellowish oil, yield 98%,

^{1}H NMR ^{1}H NMR (500 MHz, cdcl$_3$) δ 7.37 (d, J = 8.1 Hz, 2H), 7.20 (d, J = 8.6 Hz, 2H), 7.12 (d, J = 7.9 Hz, 2H), 6.82 (d, J = 8.6 Hz, 2H), 3.91 (s, 2H), 3.79 (s, 3H), 2.34 (s, 3H).

^{13}C NMR ^{13}C NMR (126 MHz, cdcl$_3$) δ 159.04, 153.20, 137.05, 133.71, 130.57, 129.71, 128.61, 128.54, 113.94, 77.29, 77.04, 76.78, 55.27, 42.75, 21.05.

HRMS (ESI): m/z [M + Na]$^+$ calcd for $C_{15}H_{16}NaOS_2$: 299.0535; found: 299.0533

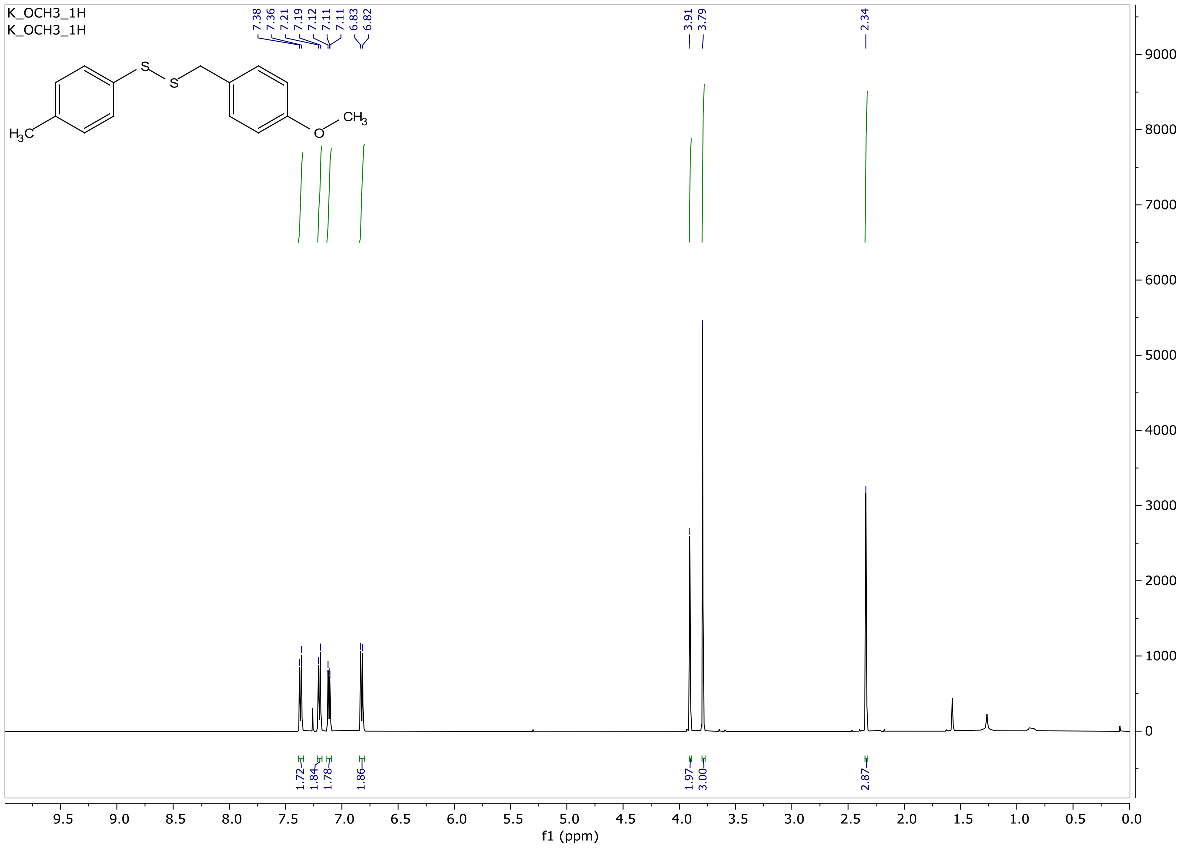

Figure S47. ^{1}H NMR spectrum of 3x

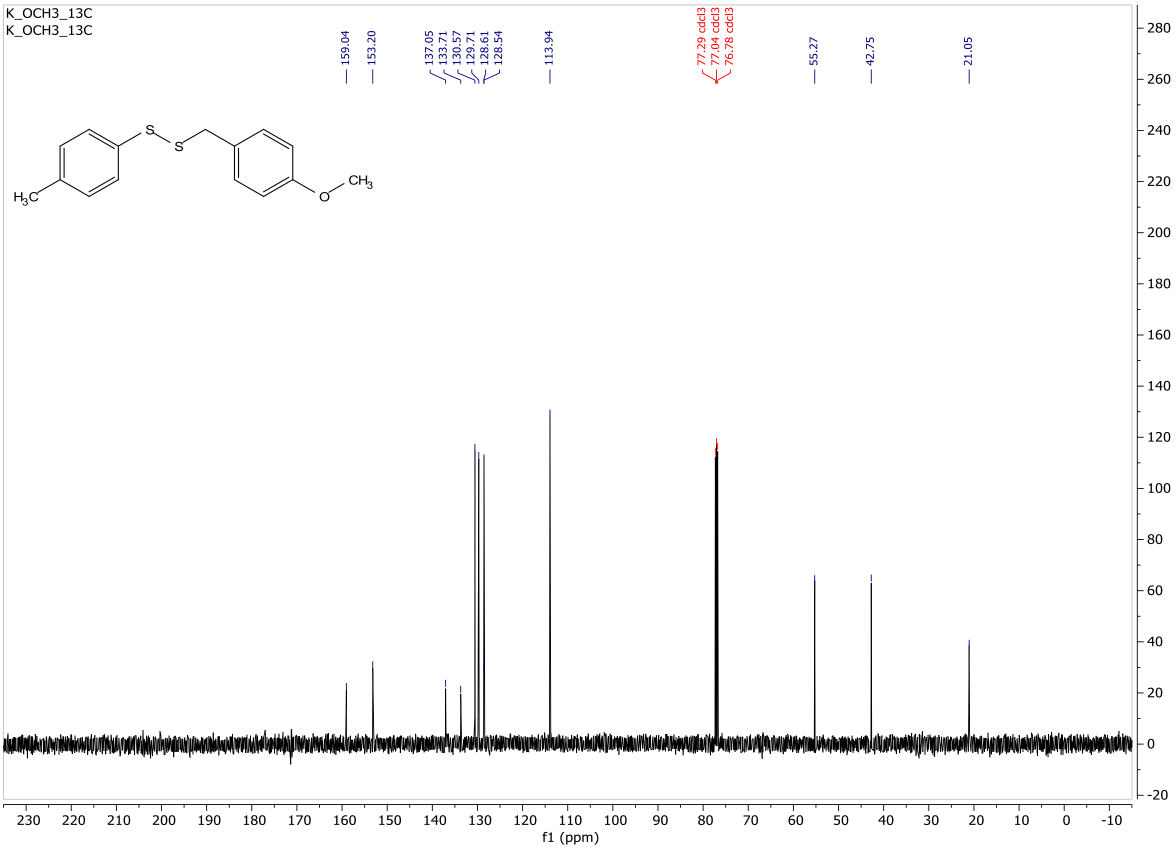

Figure S48. ^{13}C NMR spectrum of 3x

1-(p-tolyl)-2-trityldisulfane 3y

Chromatography PE, R_f =0,61, yellowish oil, yield 98%,

^{1}H NMR ^{1}H NMR (500 MHz, cdcl$_3$) δ 7.31 – 7.25 (m, 6H), 7.22 – 7.17 (m, 9H), 6.99 – 6.89 (m, 4H), 2.30 (s, 3H).

^{13}C NMR ^{13}C NMR (126 MHz, cdcl$_3$) δ 143.58, 137.02, 133.35, 130.30, 130.02, 129.95, 129.09, 127.97, 127.94, 127.67, 127.30, 126.97, 77.31, 77.05, 76.80, 21.08.

HRMS (ESI): *m*/*z* [M + Na]$^+$ calcd for $C_{26}H_{22}NaS_2$: 421.1055; found: 421.1057

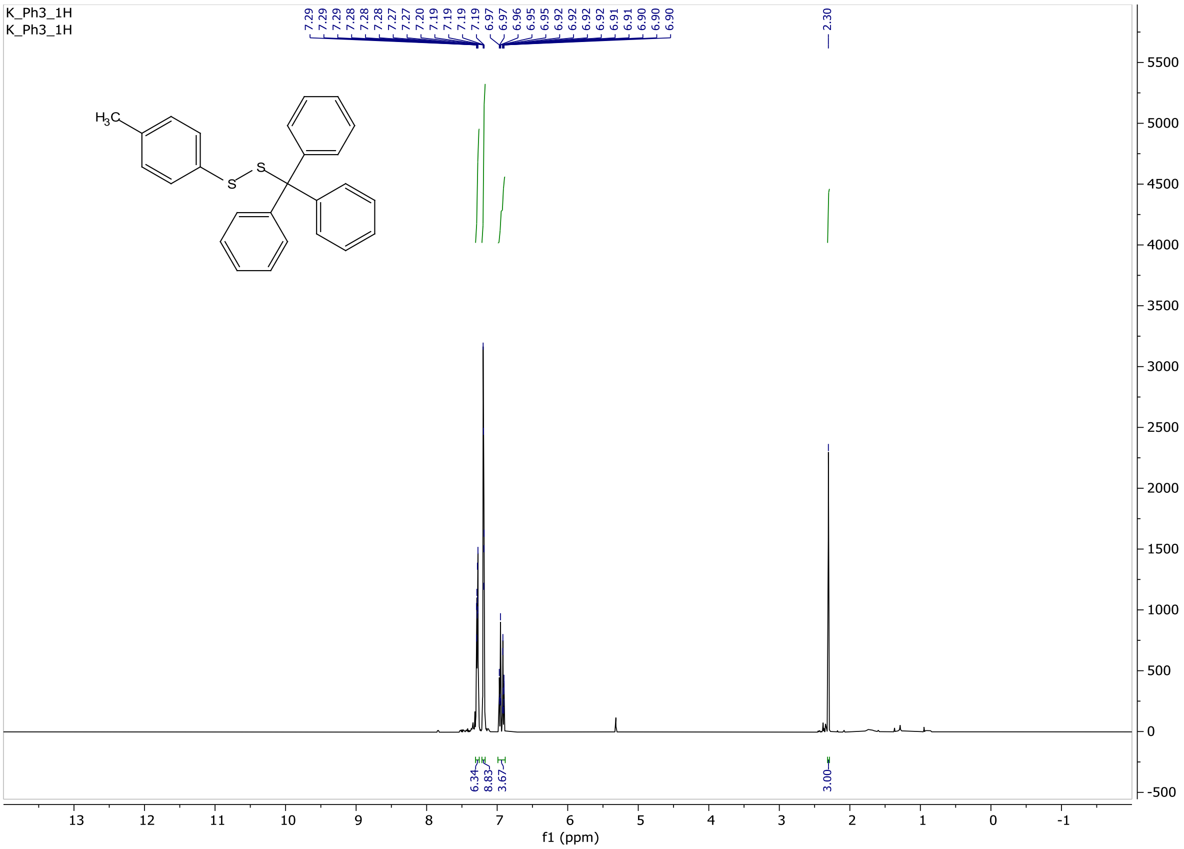

Figure S49. ^{1}H NMR spectrum of 3y

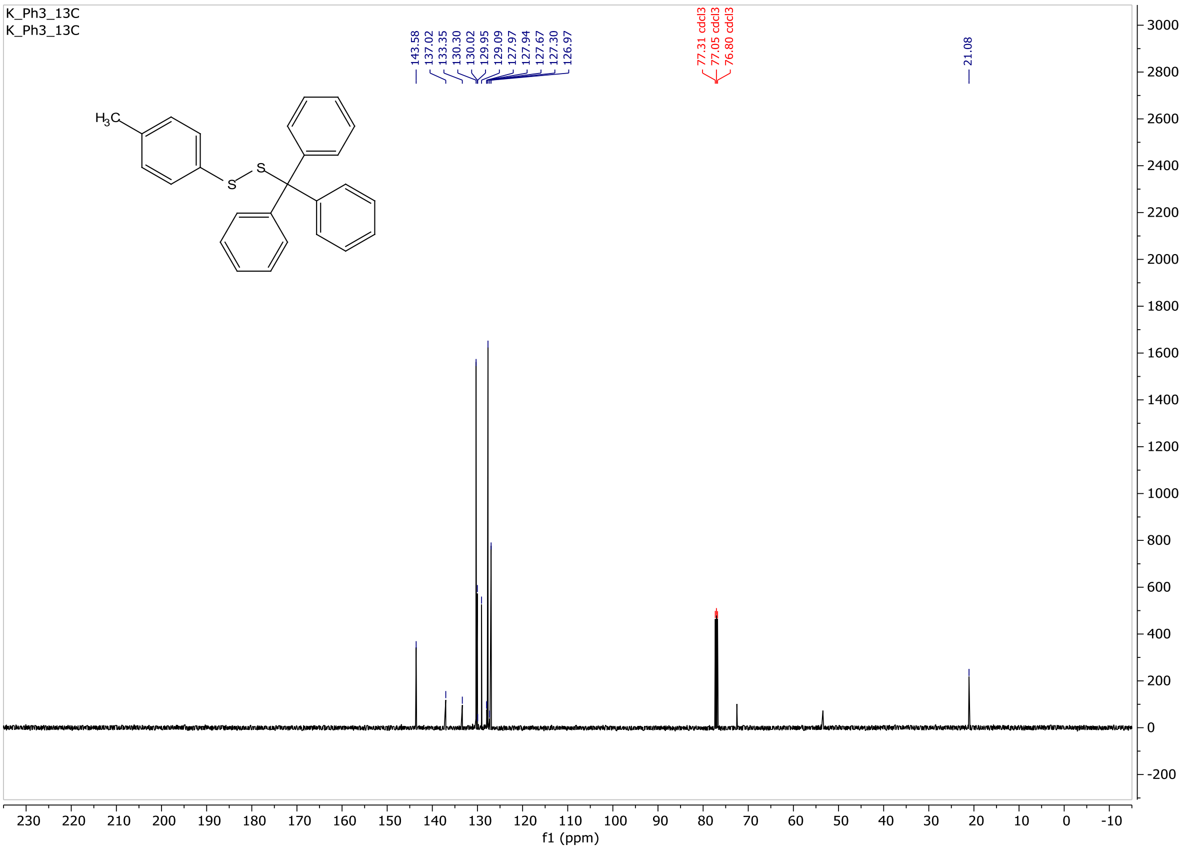

Figure S50. ^{13}**C NMR spectrum of 3y**

2-(p-tolyldisulfanyl)benzo[d]thiazole 3z

Chromatography CH_2Cl_2, R_f =0,41, yellowish oil, yield 96%,

^{1}H NMR ^{1}H NMR (500 MHz, cdcl$_3$) δ 7.89 (dt, *J* = 8.3, 0.9 Hz, 1H), 7.80 (ddd, *J* = 8.0, 1.3, 0.7 Hz, 1H), 7.59 – 7.53 (m, 2H), 7.44 (ddd, *J* = 8.3, 7.3, 1.3 Hz, 1H), 7.34 (ddd, *J* = 8.2, 7.3, 1.2 Hz, 1H), 7.20 – 7.14 (m, 2H), 2.35 (s, 3H).

^{13}C NMR ^{13}C NMR (126 MHz, cdcl$_3$) δ 154.97, 139.17, 135.89, 131.53, 130.17, 129.94, 126.28, 124.66, 122.23, 121.18, 110.00, 77.32, 77.07, 76.81, 21.20.

HRMS (ESI): *m*/*z* [M + H]$^+$ calcd for $C_{14}H_{12}NS_3$: 290.1226; found: 290.0125

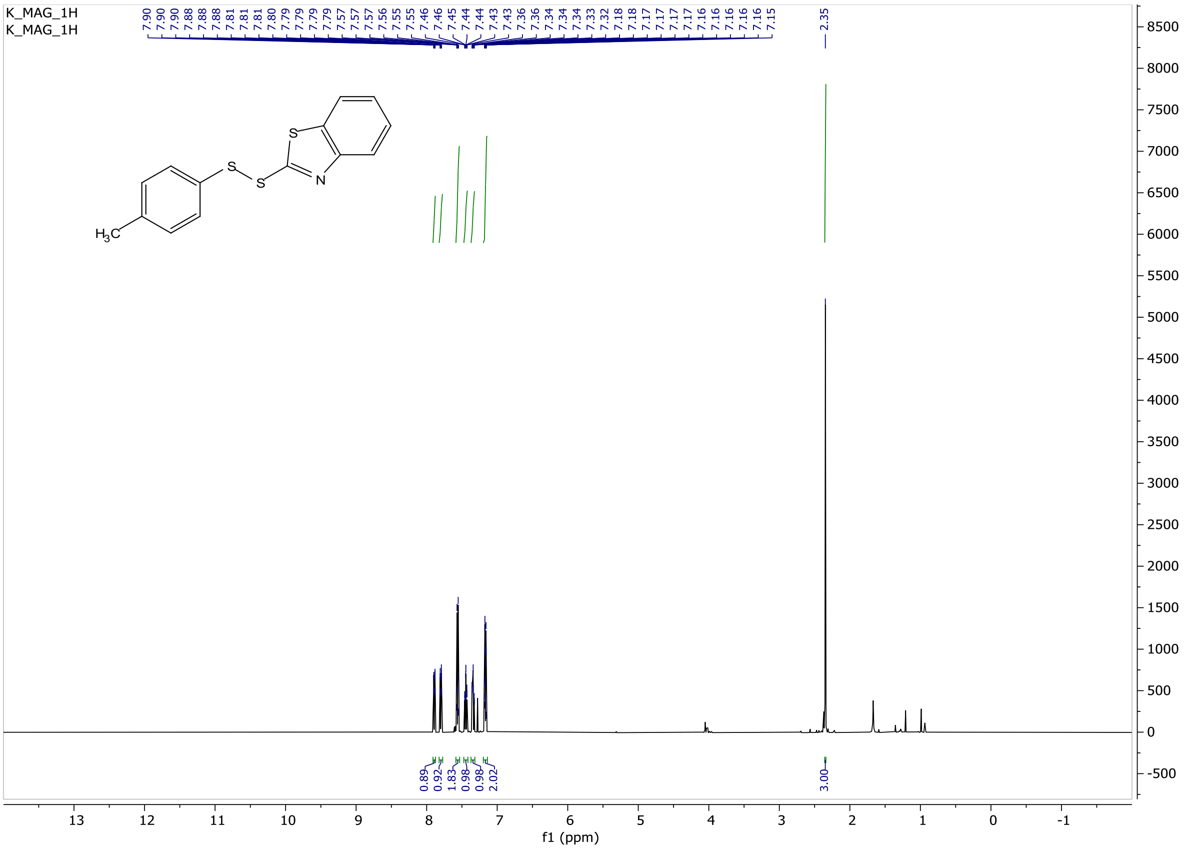

Figure S51. ^{1}H NMR spectrum of 3z

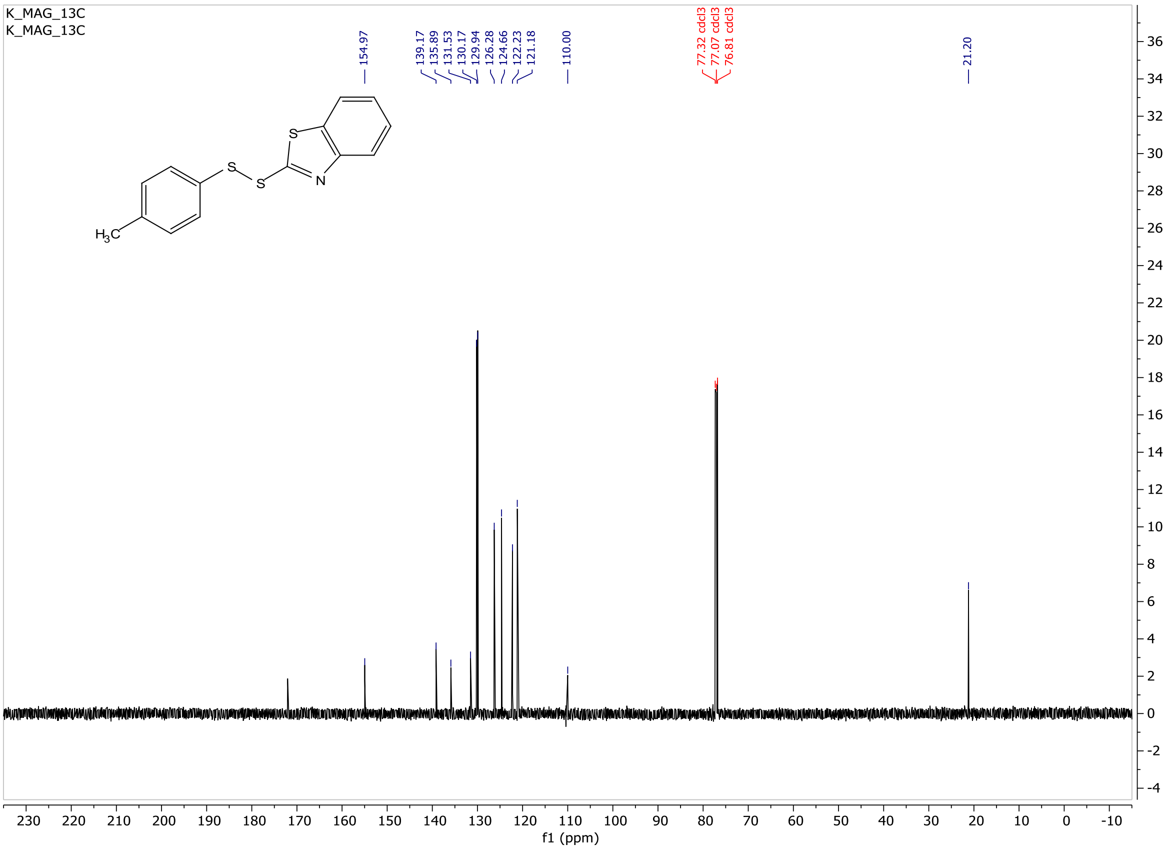

Figure S52. ^{13}C NMR spectrum of 3z

methyl 2-amino-3-(p-tolyldisulfanyl)propanoate 3aa

Chromatography CH_2Cl_2, R_f =0,41, yellowish oil, yield 96%,

^{1}H NMR ^{1}H NMR (500 MHz, cdcl$_3$) δ 7.44 (d, *J* = 8.3 Hz, 1H), 7.37 (d, *J* = 8.3 Hz, 1H), 7.14 (d, *J* = 7.9 Hz, 1H), 7.10 (d, *J* = 8.0 Hz, 1H), 4.23 – 4.14 (m, 2H), 3.79 (dt, *J* = 8.1, 4.7 Hz, 1H), 3.14 (dd, *J* = 13.6, 4.4 Hz, 1H), 2.89 (ddd, *J* = 13.7, 9.9, 7.9 Hz, 1H), 2.31 (s, 3H), 1.73 (s, 3H), 1.28 (dt, *J* = 15.2, 7.0 Hz, 3H).

^{13}C NMR ^{13}C NMR (126 MHz, cdcl$_3$) δ 153.20, 129.91, 129.78, 129.27, 128.50, 77.30, 77.04, 76.79, 61.44, 53.58, 43.68, 21.09, 14.20.

HRMS (ESI): *m/z* $[M + H]^+$ calcd for $C_{12}H_{18}NO_2S_2$: 272.0779; found: 272.0781.

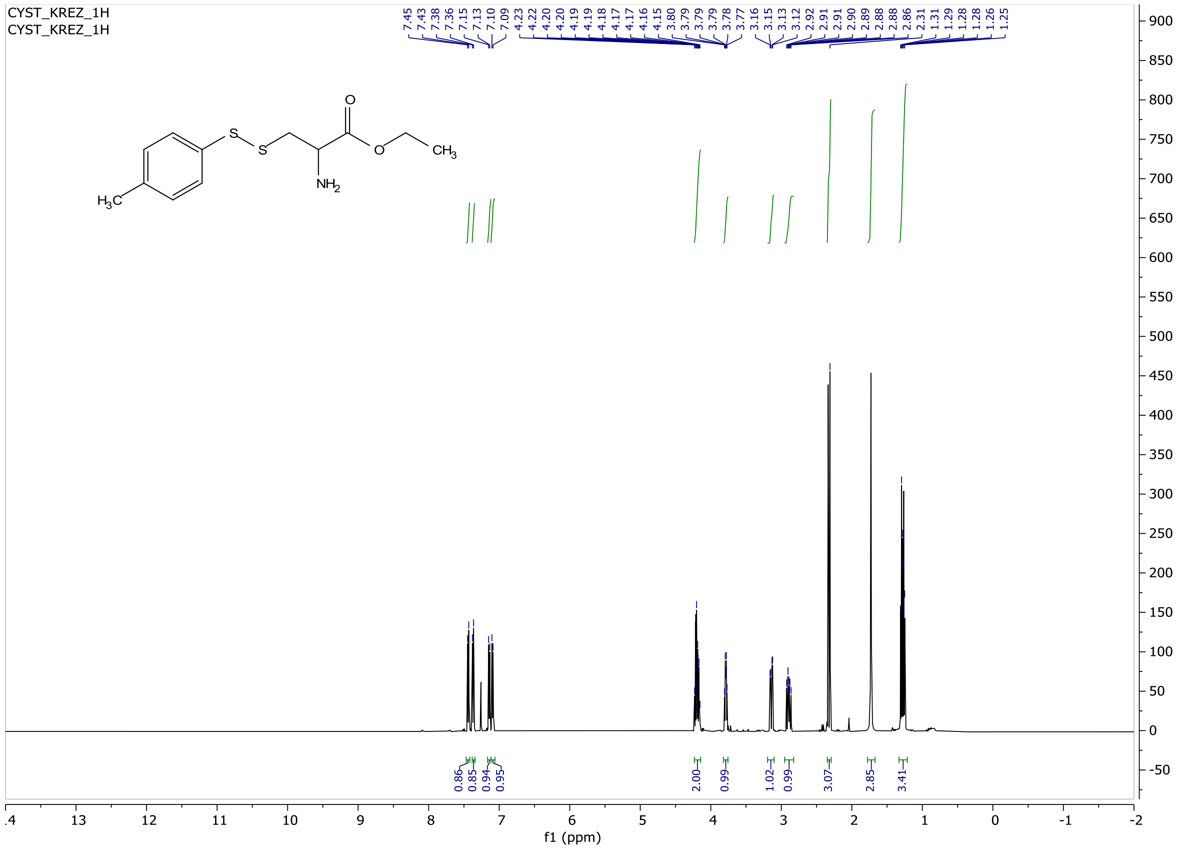

Figure S53. ^{1}H NMR spectrum of 3aa

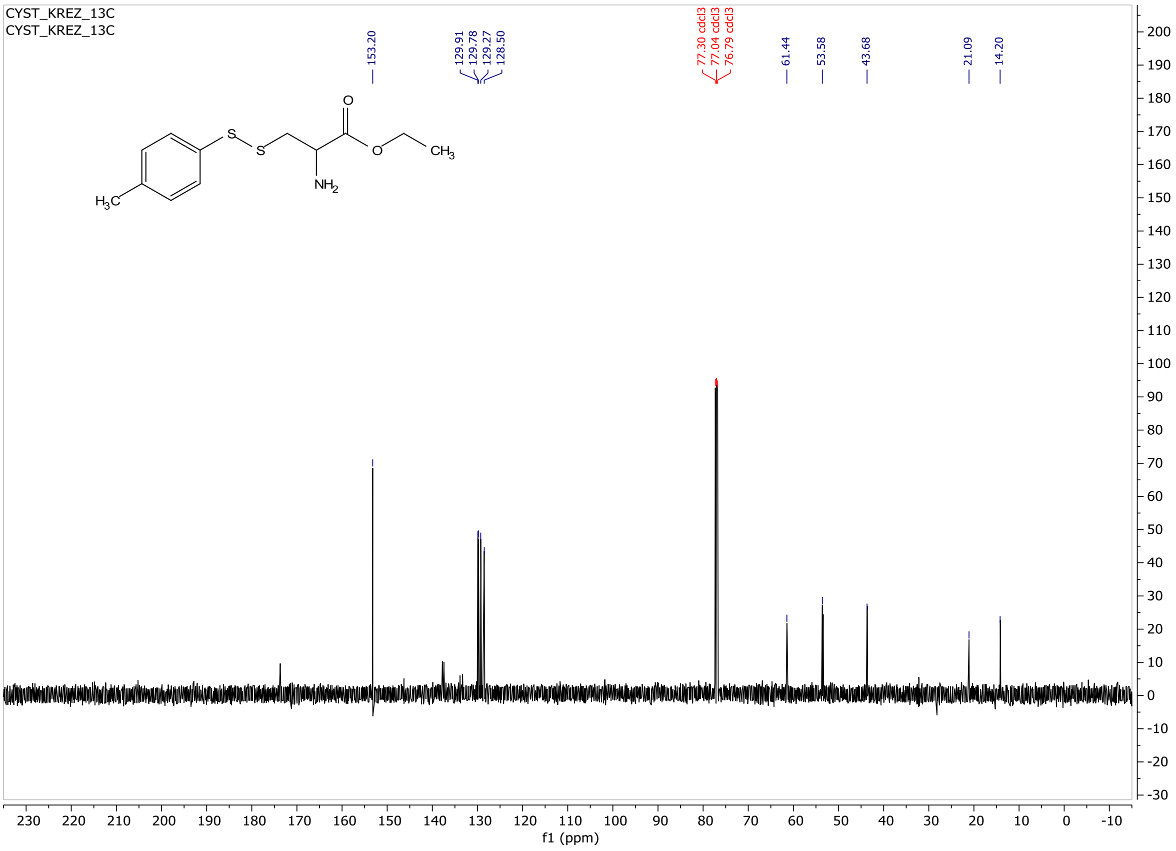

Figure S54. ^{13}C NMR spectrum of 3aa

methyl 4-(p-tolyldisulfanyl)benzoate 3ab

Chromatography CH_2Cl_2, R_f =0,41, yellowish oil, yield 98%,

^{1}H NMR ^{1}H NMR (500 MHz, cdcl$_3$) δ 7.96 (d, *J* = 8.4 Hz, 2H), 7.56 (d, *J* = 8.4 Hz, 2H), 7.37 (d, *J* = 8.1 Hz, 2H), 7.11 (d, *J* = 7.9 Hz, 2H), 3.90 (s, 3H), 2.31 (s, 3H).

^{13}C NMR ^{13}C NMR (126 MHz, cdcl$_3$) δ 166.58, 143.44, 137.95, 136.51, 132.74, 130.21, 130.13, 129.99, 129.81, 129.37, 128.46, 125.95, 77.31, 77.05, 76.80, 52.16, 21.07.

HRMS (ESI): *m*/*z* $[M + H]^+$ calcd for $C_{15}H_{15}O_2S_2$: 291.0508; found: 291.0510

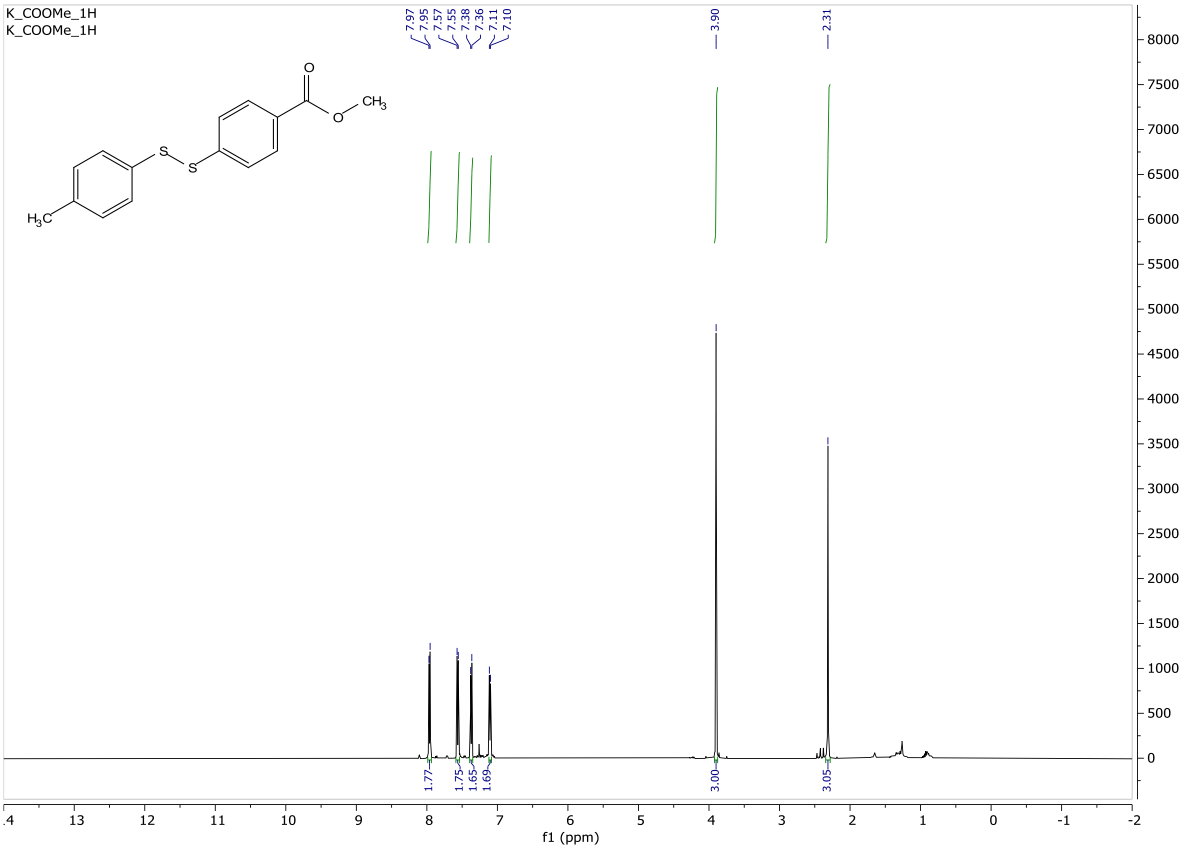

Figure S55. ^{1}H NMR spectrum of 3ab

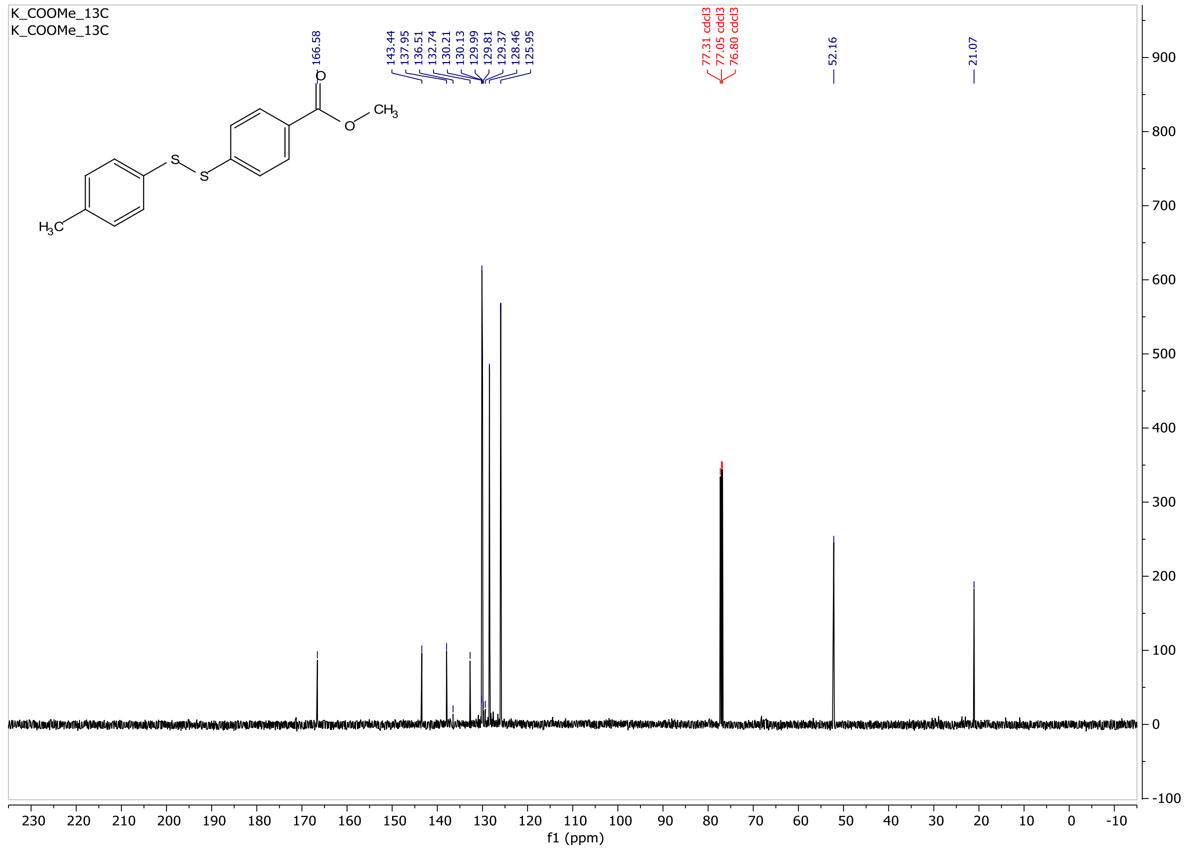

Figure S56. ^{13}C NMR spectrum of 3ab

1-benzyl-2-phenyldisulfane 3ac

Chromatography PE, R_f =0,43, yellowish oil, yield 98%,

^{1}H NMR ^{1}H NMR (500 MHz, cdcl$_3$) δ 7.51 – 7.47 (m, 2H), 7.35 – 7.27 (m, 7H), 7.27 – 7.21 (m, 1H), 3.98 (s, 2H).

^{13}C NMR ^{13}C NMR (126 MHz, cdcl$_3$) δ 137.05, 136.60, 129.44, 128.96, 128.58, 127.68, 127.60, 126.84, 77.34, 77.09, 76.83, 43.39, 43.18.

HRMS (ESI): *m/z* [M + Na]$^+$ calcd for $C_{13}H_{12}NaS_2$: 255.0273; found: 255.0270

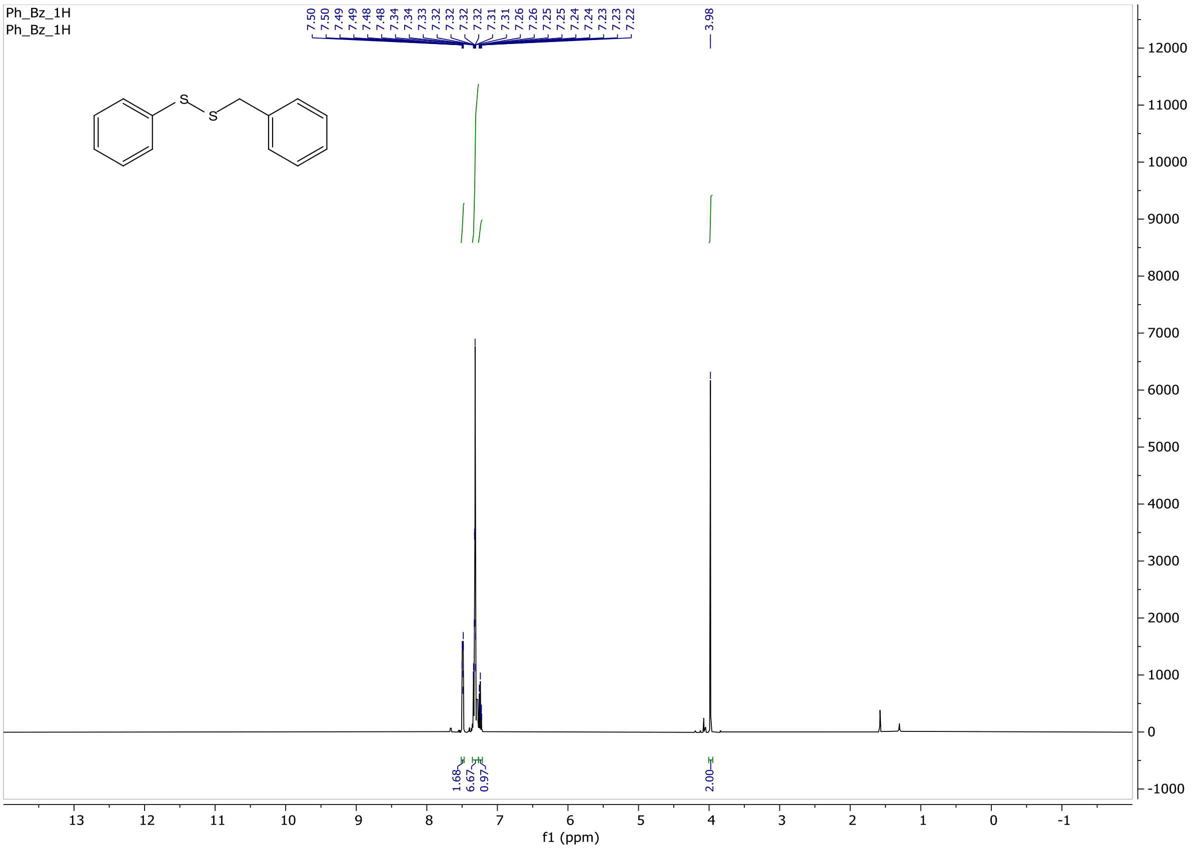

Figure S57. ^{1}H NMR spectrum of 3ac

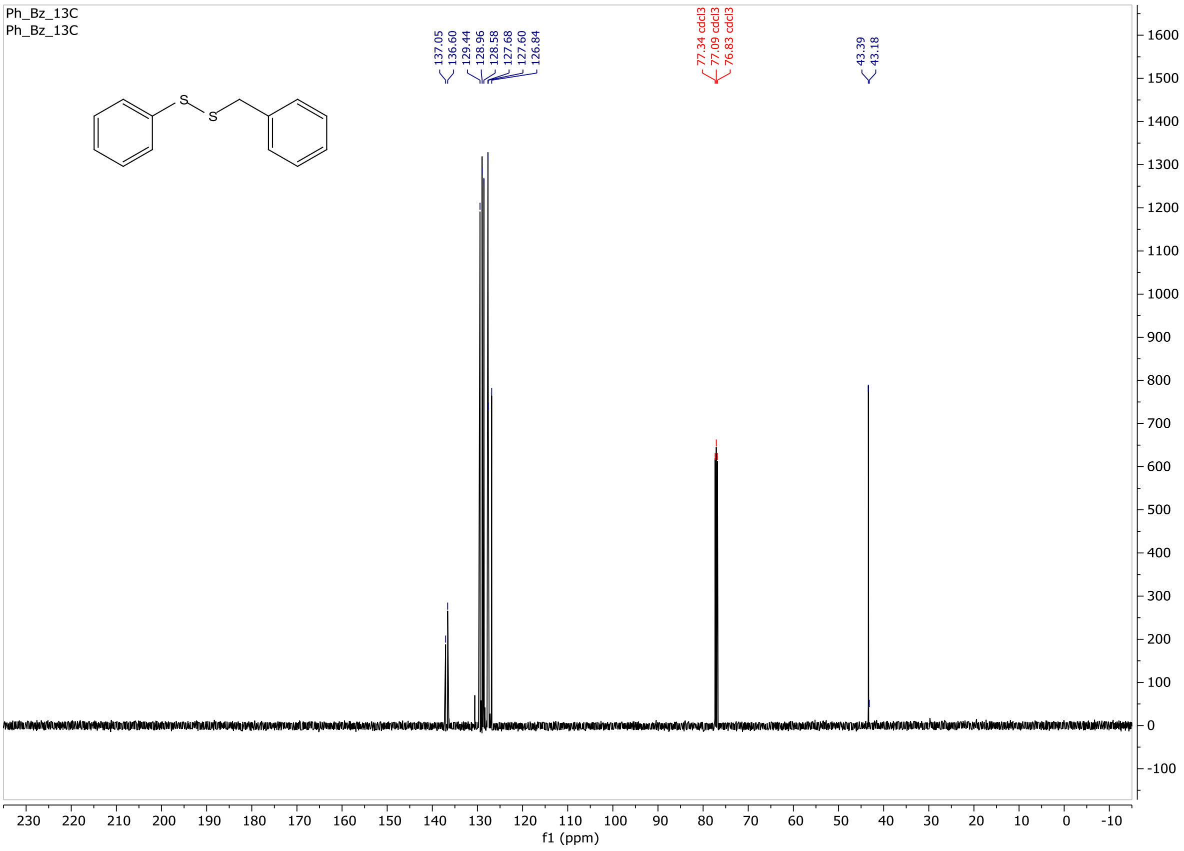

Figure S58. ^{13}C NMR spectrum of 3ac

1-(4-methoxybenzyl)-2-phenyldisulfane 3ad

Chromatography CH_2Cl_2, R_f =0,41, yellowish oil, yield 96%,

^{1}H NMR ^{1}H NMR (500 MHz, cdcl$_3$) δ 7.47 (d, *J* = 7.8 Hz, 2H), 7.30 (t, *J* = 7.6 Hz, 2H), 7.20 (d, *J* = 8.2 Hz, 3H), 6.82 (d, *J* = 8.4 Hz, 2H), 3.92 (s, 2H), 3.79 (s, 3H).

^{13}C NMR ^{13}C NMR (126 MHz, cdcl$_3$) δ 159.08, 137.16, 130.57, 128.91, 128.49, 127.57, 126.72, 113.96, 77.30, 77.05, 76.79, 55.28, 42.82.

HRMS (ESI): *m*/*z* [M + Na]$^+$ calcd for $C_{14}H_{14}NaOS_2$: 285.0378; found: 285.0379

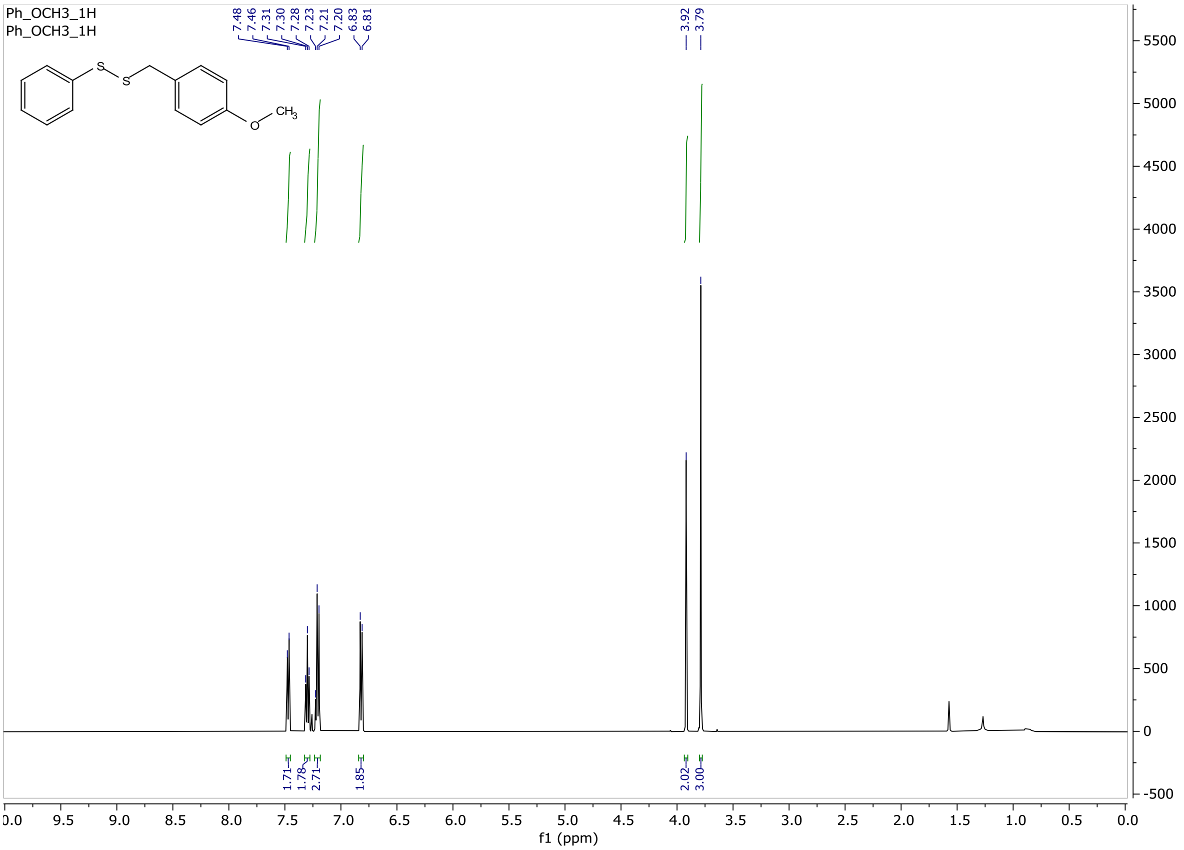

Figure S59. ^{1}H NMR spectrum of 3ad

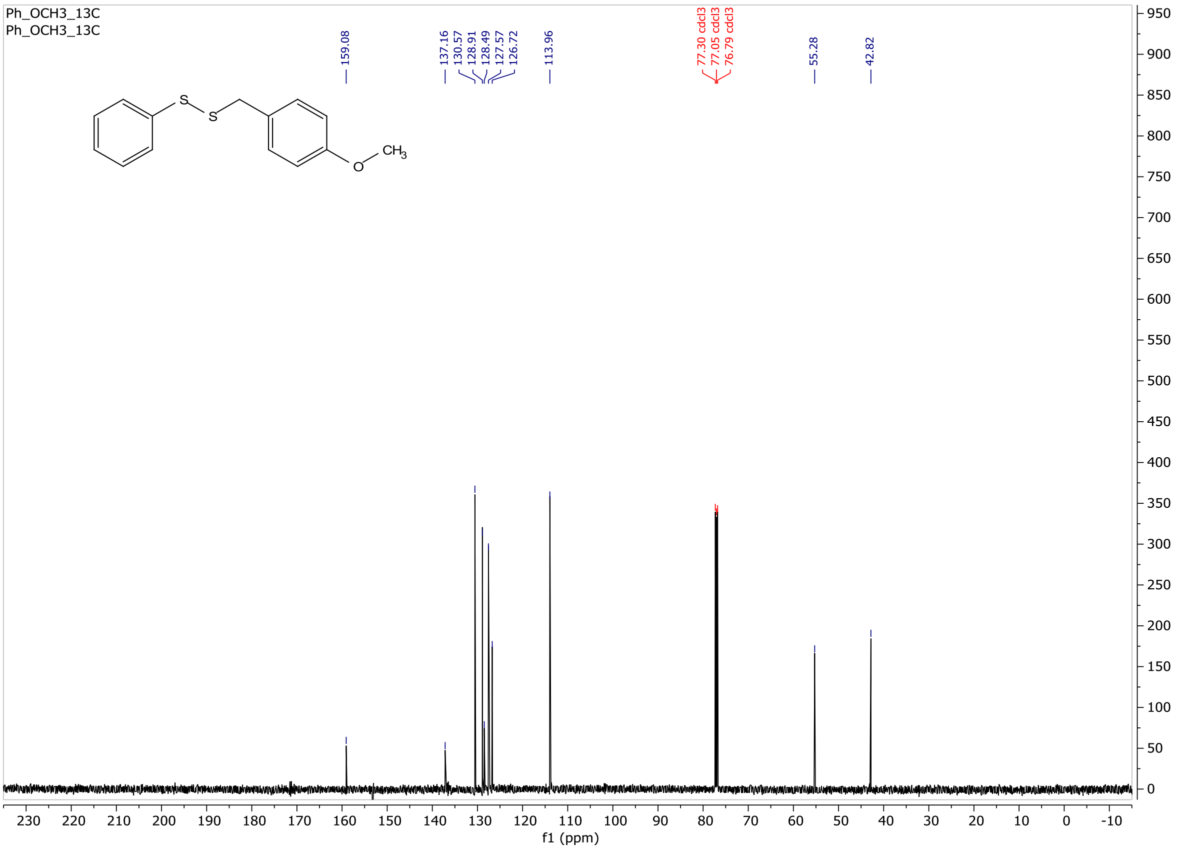

Figure S60. ^{13}**C NMR spectrum of 3ad**

1-(4-nitrobenzyl)-2-phenyldisulfane 3ae

Chromatography CH_2Cl_2, R_f =0,40, yellowish oil, yield 93%,

^{1}H NMR ^{1}H NMR (500 MHz, cdcl$_3$) δ 8.08 (d, *J* = 8.3 Hz, 2H), 7.39 (d, *J* = 8.4 Hz, 4H), 7.25 (t, *J* = 7.2 Hz, 2H), 7.20 (d, *J* = 7.3 Hz, 1H), 3.97 (s, 2H).

^{13}C NMR . ^{13}C NMR (126 MHz, cdcl$_3$) δ 144.33, 136.18, 130.23, 130.09, 128.96, 127.96, 127.19, 123.83, 123.61, 77.29, 77.04, 76.78, 42.21.

HRMS (ESI): *m*/*z* $[M + Na]^+$ calcd for $C_{13}H_{11}NNaO_2S_2$: 300.0123; found: 300.0126

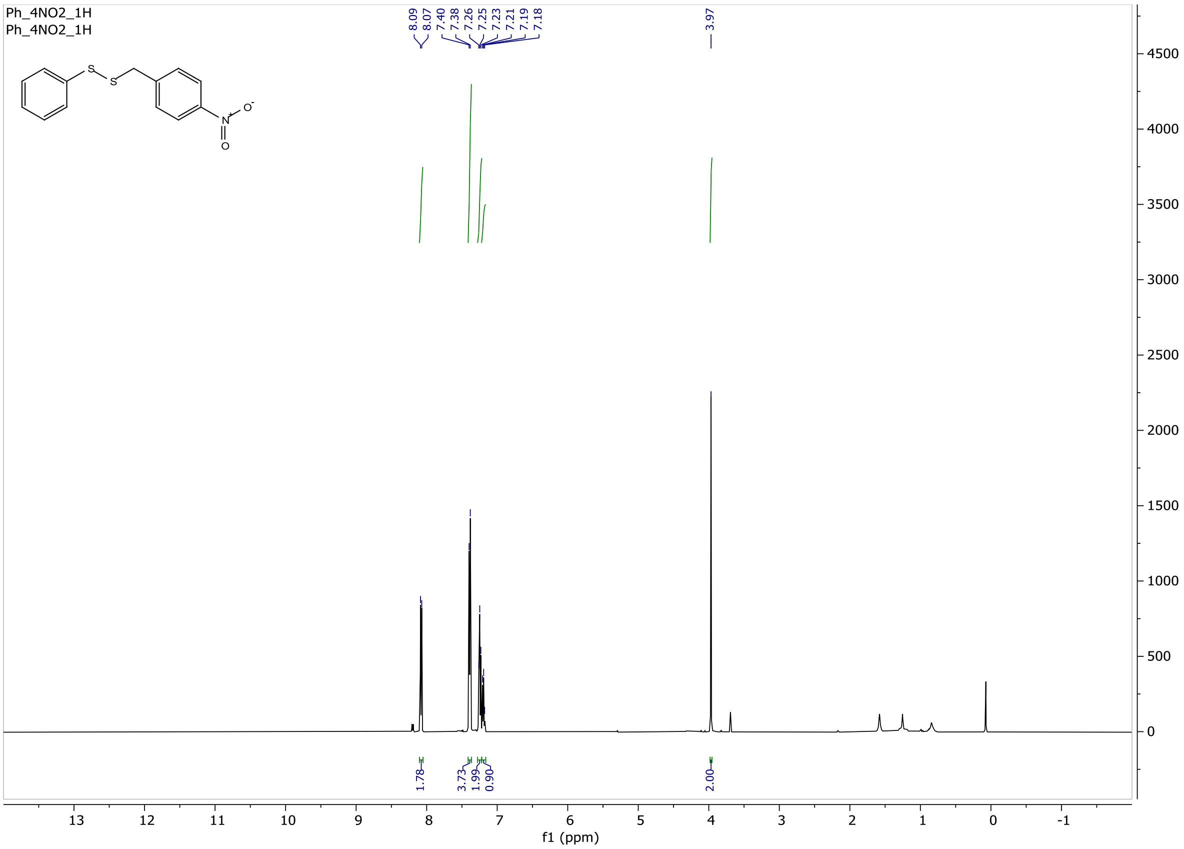

Figure S61. ^{1}H NMR spectrum of 3ae

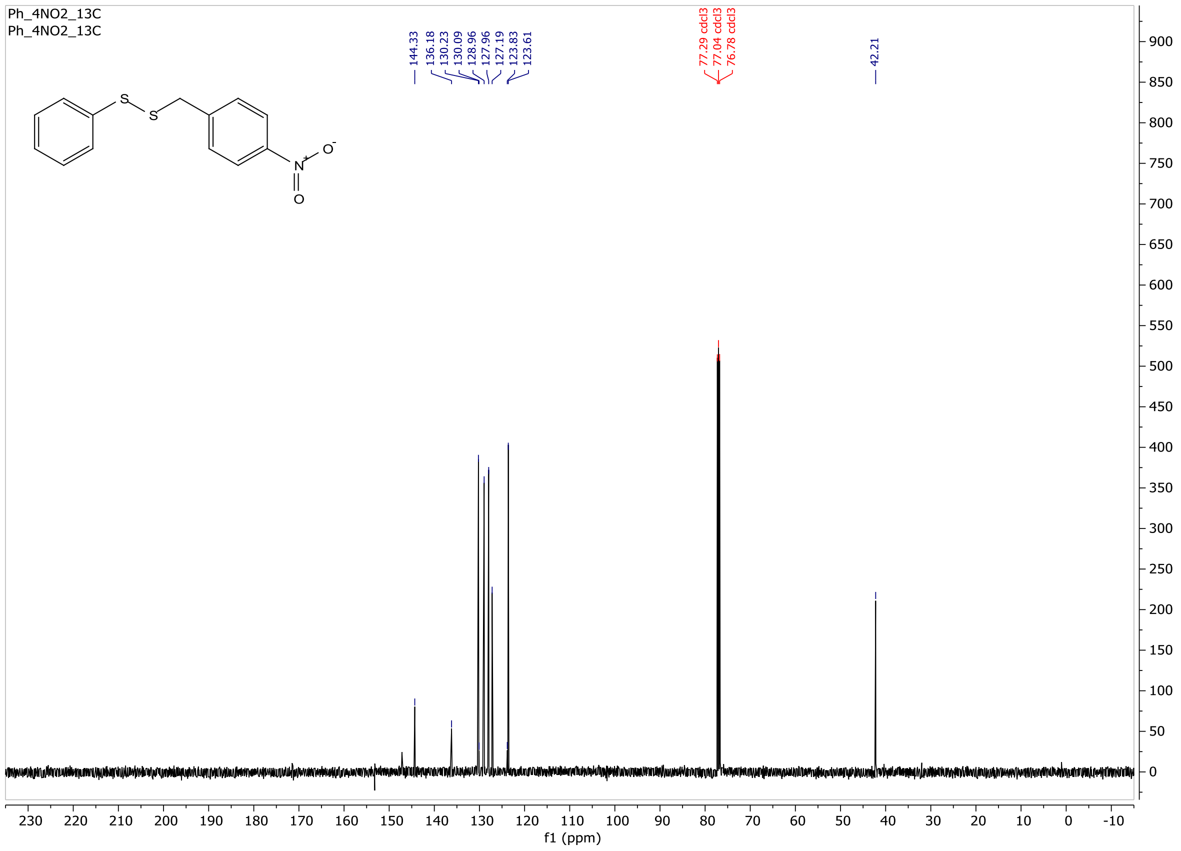

Figure S62. ^{13}C NMR spectrum of 3ae

methyl 4-(phenyldisulfanyl)benzoate 3af

Chromatography CH_2Cl_2, R_f =0,35, yellowish oil, yield 93%,

^{1}H NMR ^{1}H NMR (500 MHz, cdcl$_3$) δ 7.97 (d, *J* = 8.3 Hz, 2H), 7.56 (d, *J* = 8.3 Hz, 2H), 7.47 (d, *J* = 7.6 Hz, 2H), 7.30 (t, *J* = 7.5 Hz, 2H), 7.24 (t, *J* = 7.6 Hz, 1H), 3.90 (s, 3H).

^{13}C NMR ^{13}C NMR (126 MHz, cdcl$_3$) δ 166.55, 143.13, 136.13, 130.17, 129.21, 128.46, 127.57, 127.50, 125.88, 77.29, 77.03, 76.78, 52.18.

HRMS (ESI): *m*/*z* [M + H]$^+$ calcd for $C_{14}H_{13}O_2S_2$: 277.0351; found: 277.0349

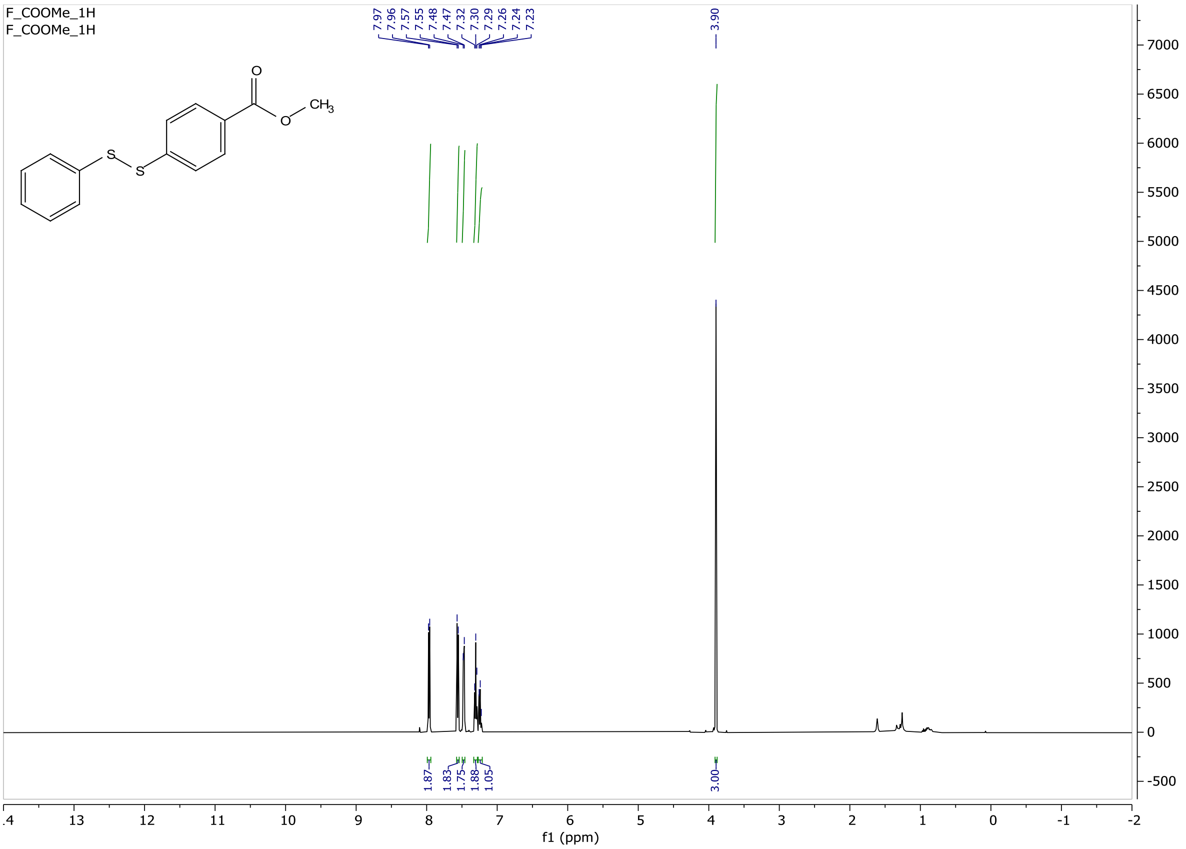

Figure S63. ^{1}H NMR spectrum of 3af

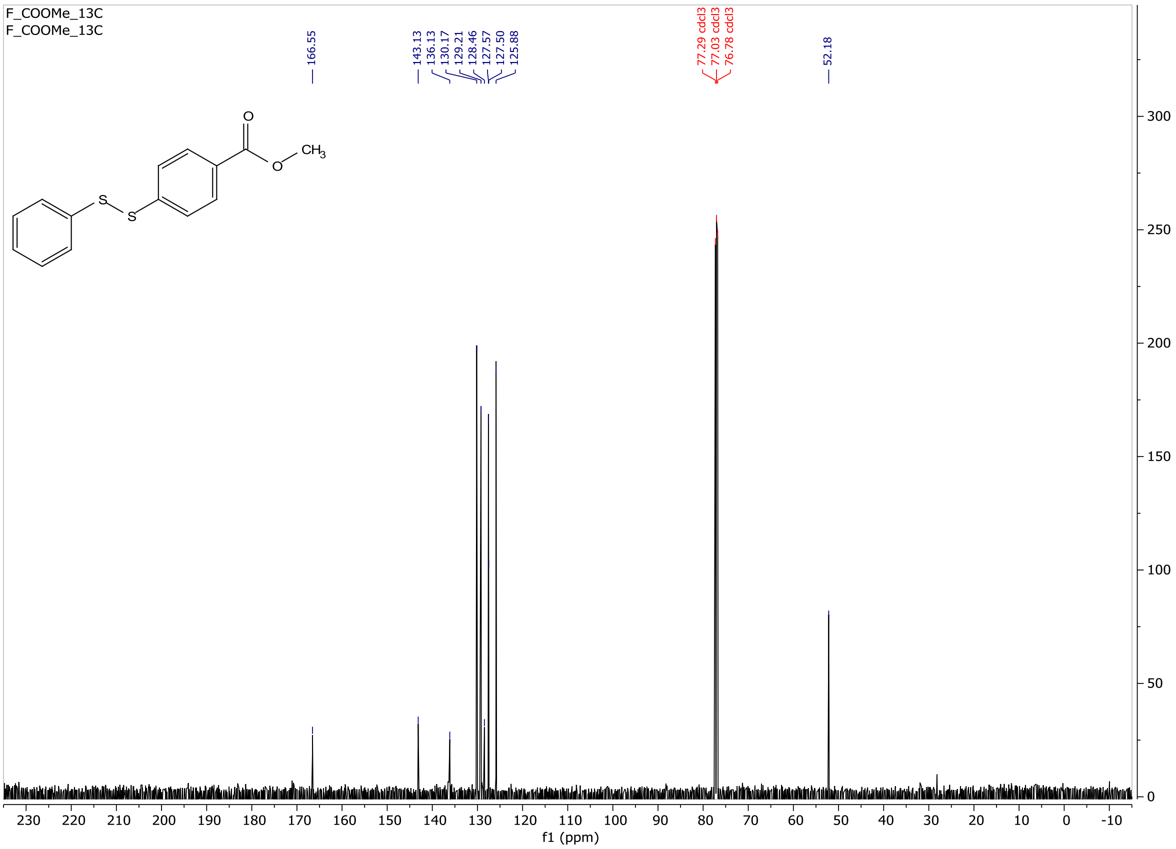

Figure S64. ^{13}C NMR spectrum of 3af
